# Barriers and facilitators to implementation, uptake and sustainability of community-based health insurance schemes in low- and middle-income countries: a systematic review

**DOI:** 10.1186/s12939-018-0721-4

**Published:** 2018-01-29

**Authors:** Racha Fadlallah, Fadi El-Jardali, Nour Hemadi, Rami Z. Morsi, Clara Abou Abou Samra, Ali Ahmad, Khurram Arif, Lama Hishi, Gladys Honein-AbouHaidar, Elie A. Akl

**Affiliations:** 1 Department of Health Management and Policy, Faculty of Health Sciences, American University of Beirut, Beirut, Lebanon; 2 Center for Systematic Review in Health Policy and Systems Research (SPARK), American University of Beirut, Beirut, Lebanon; 3 Department of Clinical Epidemiology and Biostatistics, McMaster University, Hamilton, ON Canada; 4 Faculty of Medicine, American University of Beirut, Beirut, Lebanon; 5 Hariri School of Nursing, American University of Beirut, Beirut, Lebanon; 6 Department of Internal Medicine, American University of Beirut, Beirut, Lebanon

**Keywords:** Community health insurance, Community-based health insurance scheme, Implementation, Barriers and facilitators, Universal health coverage, Low- and middle-income countries

## Abstract

**Background:**

Community-based health insurance (CBHI) has evolved as an alternative health financing mechanism to out of pocket payments in low- and middle-income countries (LMICs), particularly in areas where government or employer-based health insurance is minimal. This systematic review aimed to assess the barriers and facilitators to implementation, uptake and sustainability of CHBI schemes in LMICs.

**Methods:**

We searched six electronic databases and grey literature. We included both quantitative and qualitative studies written in English language and published after year 1992. Two reviewers worked in duplicate and independently to complete study selection, data abstraction, and assessment of methodological features. We synthesized the findings based on thematic analysis and categorized according to the ecological model into individual, interpersonal, community and systems levels.

**Results:**

Of 15,510 citations, 51 met the eligibility criteria. Individual factors included awareness and understanding of the concept of CBHI, trust in scheme and scheme managers, perceived service quality, and demographic characteristics, which influenced enrollment and sustainability. Interpersonal factors such as household dynamics, other family members enrolled in the scheme, and social solidarity influenced enrollment and renewal of membership. Community-level factors such as culture and community involvement in scheme development influenced enrollment and sustainability of scheme. Systems-level factors encompassed governance, financial and delivery arrangement. Government involvement, accountability of scheme management, and strong policymaker-implementer relation facilitated implementation and sustainability of scheme. Packages that covered outpatient and inpatient care and those tailored to community needs contributed to increased enrollment. Amount and timing of premium collection was reported to negatively influence enrollment while factors reported as threats to sustainability included facility bankruptcy, operating on small budgets, rising healthcare costs, small risk pool, irregular contributions, and overutilization of services. At the delivery level, accessibility of facilities, facility environment, and health personnel influenced enrollment, service utilization and dropout rates.

**Conclusion:**

There are a multitude of interrelated factors at the individual, interpersonal, community and systems levels that drive the implementation, uptake and sustainability of CBHI schemes. We discuss the implications of the findings at the policy and research level.

**Trial registration:**

The review protocol is registered in PROSPERO International prospective register of systematic reviews (ID = CRD42015019812).

**Electronic supplementary material:**

The online version of this article (10.1186/s12939-018-0721-4) contains supplementary material, which is available to authorized users.

## Introduction

In the past few years, there have been increased movements by governments in low and middle-income countries (LMICs) to achieve universal health coverage (UHC) [1, 2]. Under UHC, all people who need health services can receive them without undue financial hardship [3]. UHC is a critical component of sustainable development and poverty reduction, and a key element of any effort to reduce social inequalities and enhance access to care [4].

Many high-income countries that are either progressing towards or have achieved UHC have relied heavily on government or employer-based health insurance or a mix of both [5]. However, in many LMIC, financing UHC has been difficult to achieve due to limited economic resources, modest economic growth, constraints on the public sector and weak institutional capacity of government [6, 7].

Community-based health insurance (CBHI) has evolved as an alternative health financing mechanism to out of pocket payment in LMICs, particularly in areas where government or employer-based health insurance is minimal [7–10]. CBHI operates by pooling risks and resources at the community level. In such schemes, individuals or households in a community voluntarily pay a predetermined amount of money in return for a benefit package consisting of health services [11, 12].

CBHI aims to facilitate access to healthcare and increase financial protection against the cost of illness, particularly for underprivileged population [13]. For instance, CBHI schemes have been implemented in low-income countries to insure rural population and informal workers that have been excluded from regular insurance schemes [14, 15]. Evidence from systematic reviews indicate that CBHI schemes provide financial protection by reducing out-of-pocket expenditures and that such schemes improve resource mobilization and cost-recovery [12, 13].

While CBHI schemes may hold strong potential to improve financial protection and enhance utilization among their enrolled populations, there is huge variation in the effects and coverage achieved [13, 16]. This means that CBHI schemes are more likely to succeed under certain contexts and conditions [12]. Thus, simply replicating an intervention from one setting to another is likely to fail without taking into consideration the factors critical to its implementation and sustainability [17]. This, in turn, highlights a need to understand the contexts and conditions critical to the success of CBHI schemes.

Existing systematic reviews on implementation of CBHI schemes have focused on specific regions (i.e. South Asia) [18] or on a subset of outcomes, primarily uptake of or willingness to pay for CBHI schemes [19]. This systematic review adds to the extant reviews the following: given that our search includes studies published in all LMIC countries, we provide a much more global perspective than the South Asian alone. In addition, we identified all factors influencing implementation, enrollment, and sustainability of implemented CBHI schemes (and not proposed schemes), using an ecological perspective that takes into account the individual, interpersonal, community and systems level perspective. Findings from this systematic review can help inform the decisions of policymakers and stakeholders considering to implement CBHI within their own context.

## Methods

### Protocol and registration

We registered the review protocol in PROSPERO International prospective register of systematic reviews (ID = CRD42015019812).

### Eligibility criteria

- Study design: All studies that were eligible were peer reviewed publications or grey literature, published in English language and after year 1992. We included randomized trials, non-randomized studies (e.g., prospective studies, retrospective studies, before and after studies and cross-sectional studies), qualitative studies, process evaluation studies, policy analysis studies, and case studies.. We excluded editorials, commentaries, proposals, conferences, and systematic reviews. We also excluded policy analysis papers and case studies that lacked a clear methodology section.
- Setting: low- and middle- income countries (as defined by the World Bank). The World Bank defines low- income economies as those with a Gross National Income (GNI) per capita of $1025, (U.S. dollars) and middle-income economies as those with GNI per capita between $1026 and $4035 [20].
- Interventions: community-based health insurance (CBHI) schemes. We excluded disease-specific schemes, vouchers, conditional cash transfer, social or national health insurance schemes or the extension of the latter two to the informal sector. We also excluded studies that looked at integration as opposed to implementation of specific programs. In addition, we excluded studies that focused on proposed CBHI schemes (i.e., the scheme was not implemented in an actual setting).
- Outcome**:** barriers and facilitators to the implementation uptake and sustainability of CBHI schemes. We also included studies that described the process of implementation or assessed strategies to promote the implementation of CBHI schemes. Whenever available, we reported on interventions to overcome identified barriers. We excluded studies that assessed the impact of schemes on health and financial outcomes without considering factors contributing to the success or failure thereof. We also excluded studies that focused on payment methods or utilization of healthcare services in general without any linkage to CBHI schemes.

### Search strategy

We searched the following electronic databases between December 2014 and January 2015: PubMed, MEDLINE, EMBASE, WHO Global Health Library, and Health Systems Evidence. We developed and validated the search strategy with the help of an information specialist. The strategies combined three different concepts: ‘health insurance scheme’, ‘barriers and facilitators’ and ‘low- and middle-income countries’. Additional file 1 provides the free text terms and MeSH (Medical Subject Headings) terms used to search the different electronic databases. We restricted searches to English language and from 1992 forward. We chose this start date because the concept of ‘health benefit packages’ took centre-stage in the debate when the 1993 World Development Report raised the question on how governments, especially in LMIC, should spend their limited health budgets [21].

We complemented the electronic database searches with a variety of approaches to identify additional literature, including grey literature. We manually searched Google Scholar and the websites of relevant institutions like the World Health Organization (WHO) and the World Bank. We also screened the reference lists of included studies and relevant systematic reviews. In addition, we contacted the authors of conference proceedings that are of potential relevance.

### Study selection

Prior to the selection process, and in order to enhance its reliability, all the reviewers participated in a calibration exercise using a randomly chosen sample of 150 citations. The selection process consisted of two stages, title and abstract screening and full text screening. Teams of two reviewers (RF, NH, RM, and CA) worked in duplicate and independently to screen the titles and abstracts of identified citations for potential eligibility. They obtained the full texts of citations judged as potentially eligible by at least one of the reviewers. Then, the teams of two reviewers screened the full texts independently and in duplicate. At this stage, the reviewers compared results and resolved disagreements by discussion or with the help of a third reviewer (FJ or EAA) if disagreement could not be resolved. They used standardized and pilot-tested screening forms. They documented the reason for study exclusion.

### Data abstraction

We conducted calibration exercises on a randomly chosen sample to ensure adequate agreement. Teams of two reviewers (RF, NH, RM, CA and LH) abstracted data from eligible studies in duplicate and independently. They resolved disagreement by discussion or with the help of a third reviewer (if they could not reach an agreement).

They used a standardized data abstraction form to collect information on the following variables: study information (authors, year of publication, and study design), objective, methods (sample size and methods, timeframe, data collection, data analysis), population (sample population, setting), description of scheme (type of scheme, content of services covered, enrollment rate, unit of enrollment, source of fund, premium, cost-sharing, role of government, provider-payment method), socio-demographic factors, and reported barriers and facilitators.

### Quality assessment

Two reviewers (RF, LH) assessed the quality of included studies in duplicate and independently. They resolved disagreement by discussion or with the help of a third reviewer.

We used Cochrane risk of bias tool to assess the risk of bias in randomized studies; a modified version of the Cochrane risk of bias tool, adapted from Alkhaled et al. (2014), to assess the risk of bias in non-randomized quantitative studies [22]; the Critical Appraisal Skills Program (CASP) tool to assess the quality of qualitative studies; and a tool adopted from Niezen and Mathijssen (2014) to assess the methodological quality of mixed-methods studies that did not analyze quantitative and qualitative data separately [23]. We did not exclude any study based on the results of the quality assessment. In this review, quality of primary studies is not as critical because we judged that every study may offer valuable insights on the various factors influencing CBHI [24, 25].

### Data analysis and synthesis

Given the heterogeneity in study design, settings, and outcome measures, we did not conduct meta-analysis. Instead, we synthesized the findings narratively, making use of both thematic [26] and framework analysis [27]. We used a slightly modified version of the Ecological Model framework to categorize emerging themes into the individual, interpersonal, community, and systems level [28].

Data coding involved three phases: deduction (coding data and labeling each section), induction (screening data for new concepts or codes to emerge), and verification (verifying all coded data) [27]. We reviewed the literature on CBHI schemes to generate an initial list of coding themes corresponding to each level of the ecological model (See Additional file 2). Then, the reviewers screened the “result” section of each included study and coded the findings under one of the predefined themes, while also allowing for new themes to emerge inductively. We iteratively updated the coding themes as we proceeded with data analysis [29]. Throughout this process, team members with subject expertise were consulted to validate coding decisions and discuss emerging themes. We revisited and considered data in the context of any newly emergent theme. All studies were coded at least twice, once with the initial pre-defined list, and once with the finalized list of coding themes [30]. We narratively present the main barriers to implementation, uptake, or sustainability of CHBI schemes and strategies that facilitated them, organized according to the Ecological Model framework into individual, interpersonal, community, and systems level.

## Results

### Study selection

**Fig. 1:**
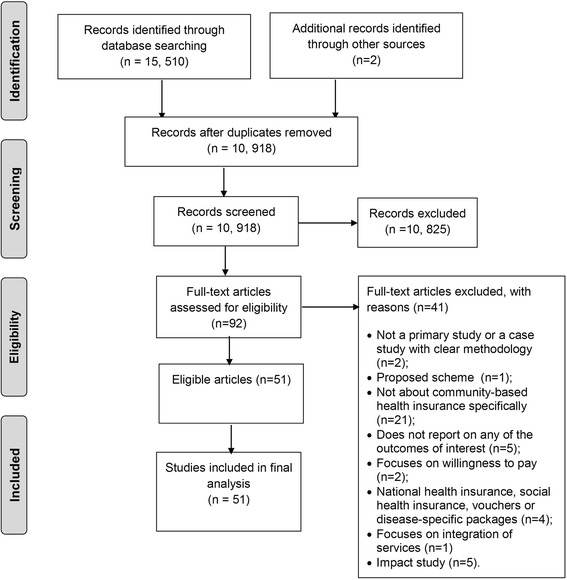
Study flowchart

Figure 1 shows the flow chart summarizing the process of study selection. Of the 15,510 citations identified, 44 articles reporting on 51 studies met the eligibility criteria (one report included three different surveys of CBHI schemes in Ghana [31], while a second report included five studies conducted in different countries [32–36]. Additional file 3 provides a list of the excluded studies with reasons for exclusion.

### Characteristics of included studies

Additional file 4 provides an overview of the characteristics of the 51 included studies. The studies were published between 1997 and 2014 (inclusive) and were conducted in 22 countries across three continents. The study design varied across studies: cross-sectional studies (*n* = 22); randomized controlled trials (*n* = 1); qualitative studies (*n* = 8), case studies (*n* = 6) and mixed methods studies (*n* = 14). The mixed methods studies included a mix of surveys, interviews, focus groups and/or documentary analysis, of which eight did not differentiate between the quantitative and qualitative data.

### Quality appraisal

We judged the studies reporting qualitative data to have met most of the CASP tool checklist for methodological quality. However, all studies failed to establish sufficient relationship between researcher and participants.

We judged the risk of bias in the RCT as ‘unclear’ due to lack of adeuqate information provided by the authors [37]. Of the studies reporting quantitative data, we judged five to be at ‘low’ or ‘unclear’ risk for all criteria assessed [11, 33, 38–40] and one to be at ‘high’ or ‘unclear’ risk for all criteria assessed [41]. The risk of bias varied across criteria for the remaining studies.

We could not find appropriate quality appraisal tools to assess the quality of the six case studies given their descriptive nature (Additional file 5).

### Reported barriers and facilitators

We narratively present the findings according to the following levels of the Ecological Model:

1. Individual
2. Interpersonal
3. Community
4. Systems: governance arrangement
5. Systems: financial arrangement
6. Systems: delivery arrangement

**Fig. 2:**
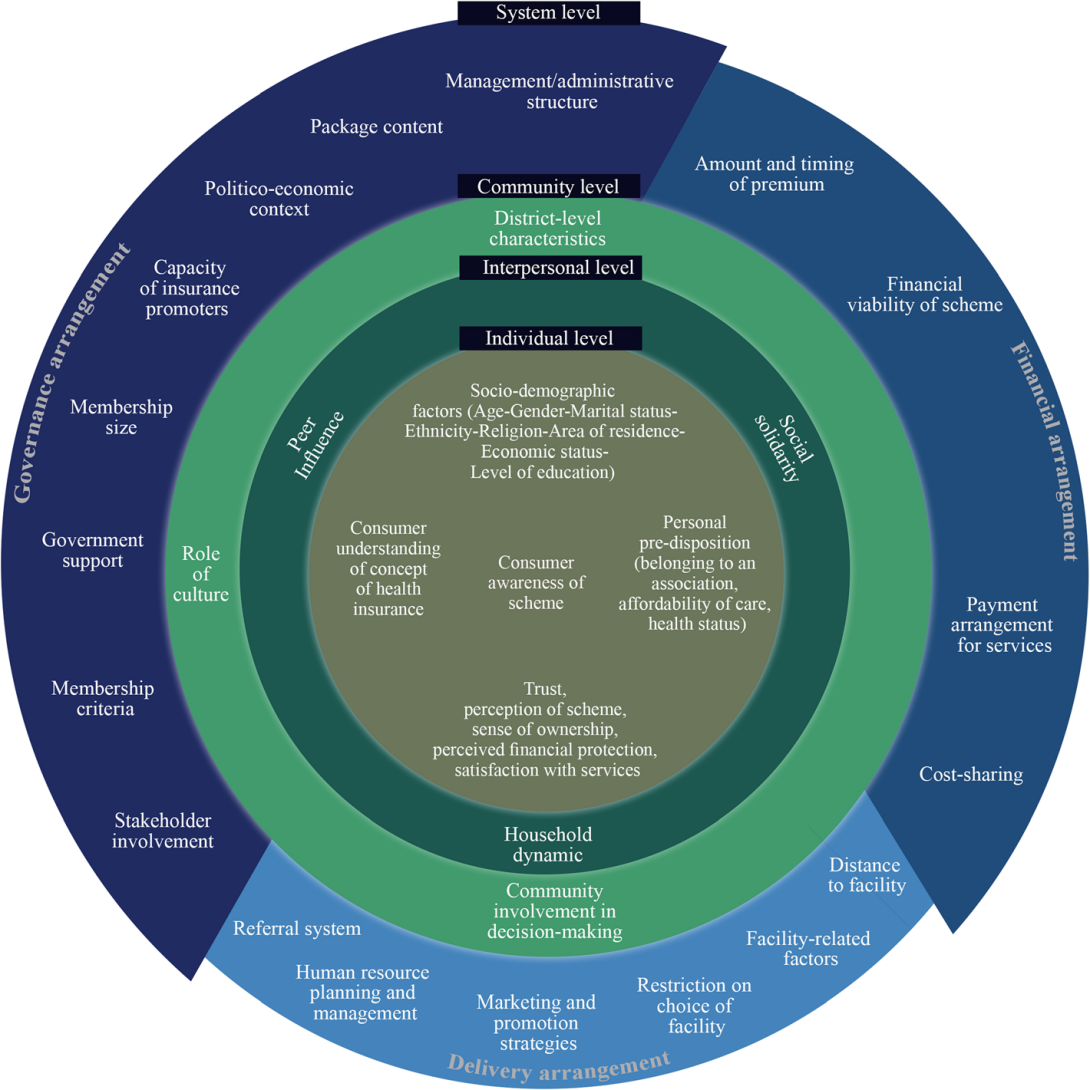
A conceptual framework of factors influencing implementation, uptake, and sustainability of community-based health insurance schemes

Under each level and within each theme, we specified whether the factor influenced implementation, uptake or sustainability of CBHI. For the purpose of this review, we conceptualized ‘implementation’ as operation of a scheme, ‘uptake’ as enrollment into a scheme, and ‘sustainability’ as renewal or drop out of a scheme or in terms of viability of the scheme. Findings are also summarized in a conceptual framework (Fig. 2).

#### 1. Individual level

Themes included consumer awareness, consumer understanding of the concept of health insurance, attitude factors, personal predispositions, and socio-demographic characteristics (see Table 1 and Additional file 6).

**Table 1:** Summary of key findings under individual level

Individual-level factors	Number and type of studies ^a^	Reported as barrier	Reported as facilitator	Related to ^b^		
				Implementation	Uptake	Sustainability
Consumer awareness and understanding of scheme						
Consumer awareness of scheme	N = 6 Cross-sectional (3); Mixed (3)	N = 2 [42, 44]	*N* = 4 [36, 41, 43, 45]		X	
Consumer understanding of concept of health insurance	N = 15 Qualitative (2); Cross-sectional (5); Mixed (5); Case studies (3)	*N* = 10 [31, 38, 46–48, 53–57]	N = 5 [41, 49–52]		X	X
Attitude factors						
Consumer trust in insurer	N = 12 Qualitative (1); Cross-sectional (6); Mixed (4); Case studies (1)	N = 6 [42, 46, 52, 53, 55, 58]	*N* = 6 [31, 33, 45, 58–60]		X	X
Sense of ownership of scheme	N = 5 Cross-sectional (3); Mixed (2)	N = 1 [53]	N = 4 [33, 47, 58, 61]		X	X
Perceived financial risk protection	N = 4 Qualitative (2); Mixed (2)	–	N = 4 [47, 56, 62, 63]		X	X
Perceived quality of care	N = 10 Cross sectional (5); Case study (1); Mixed (4)	N = 6 [42–44, 53, 65, 73]	N = 4 [45, 52, 58, 64]		X	X
Satisfaction with services	N = 5 Cross-sectional (5)	N = 3 [38, 52, 65]	N = 2 [36, 66]			X
Personal pre-disposition						
Previous experience with local groups	N = 5 Cross-sectional (2); Mixed (3)	N = 3 [33, 43, 47]	N = 2 [32, 67]		X	
Affordability of care	N = 4 Cross-sectional (2); Qualitative (1); Case study (1)	N = 2 [48, 54]	N = 2 [33, 52]		X	X
Health status	*N* = 18 RCT (1); Cross-sectional (12); Mixed (5)	N = 4 [11, 38, 56, 65]	N = 15 [32, 34–37, 39, 40, 45, 56, 58, 66–70]		X	X
Socio-demographic factors						
Age (middle to old age)	N = 9 Cross-sectional (6); Mixed (3)	–	N = 9 [34, 35, 39, 40, 43, 58, 65, 67, 71]		X	
Gender (female)	N = 4 Cross-sectional (3); Mixed (1)	–	N = 4 [39, 40, 45, 71]		X	
Being married	N = 4 Cross-sectional (2); Mixed (2)	–	N = 4 [35, 39, 45, 68]		X	
Being employed	N = 4 Cross-sectional (3); Mixed (1)	–	N = 4 [35, 36, 45, 66]		X	
Ethnicity (minority)	N = 3 Cross-sectional (1); Mixed (1); Case study (1)	N = 3 [45, 49, 64]	–		X	
Migration status	N = 1 Cross-sectional (1)	N = 1 [39]	–		X	
Religious affiliation (Christian)	N = 4 Cross-sectional (2); Mixed (2)	N = 2 [68, 70]	N = 2 [32, 65]		X	
Occupational setting (rural)	N = 2 Cross-sectional (1); Mixed (1)	N = 1 [65]	N = 1 [43]		X	
Per capita expenditure (higher level)	N = 4 Cross-sectional (3); Mixed (1)	N = 1 [65]	N = 3 [58, 64, 67]		X	
Economic status (higher level)	N = 14 Cross-sectional (9); Mixed (5)	N = 2 [66, 68]	N = 12 [11, 32–35, 39, 45, 56, 58, 64, 67, 70]		X	
Education (higher level)	N = 10 Cross-sectional (7); Mixed (3)	N = 3 [35, 36, 66]	N = 7 [40, 43, 45, 64, 65, 67, 68]		X	

^a^ Some of the studies included both barriers and facilitators

^b^ X symbol denotes whether the factor relates to implementation, uptake or sustainability

##### Consumer awareness of scheme (*n* = 6)

Six studies conducted in Burkina Faso, Cameroon, India, La PDR, and Thailand found that consumer awareness of scheme existence was a significant determinant of scheme uptake [36, 41–45]. Individuals living in rural areas [44], and those of low level of education [41] reported the lack of awareness as a barrier. Initiatives to overcome this lack of awareness included regular house visits, awareness campaigns, mass media, and sensitization by scheme staff, scheme members and local churches [41–43, 45].

##### Consumer understanding of the concept of health insurance (*n* = 15)

Consumer understanding of the concept of health insurance was reported to influence uptake and sustainability of a CHBI scheme.

Studies conducted in Afghanistan, Cameroon, China, Ghana, Guatemala, India, Kenya, Nigeria, the Philippines, Tanzania and Uganda found that understanding of the concept and principles of health insurance by household members played an important role in their decisions to enroll in CBHI schemes [31, 38, 41, 46–52].

As for sustainability, members who did not understand the concept of risk pooling (i.e., premiums would not be paid back if they do not utilize the service) and the purpose of co-payment dropped out, leading to the failure of many schemes [53–57].

##### Attitude factors (*n* = 24)

Emerging themes under this category included consumer trust in scheme insurer, sense of ownership of scheme, perceived financial risk protection, perceived quality of care, and consumer satisfaction with services provided by scheme. All factors influenced both uptake and sustainability, except for consumer satisfaction which influenced sustainability only. The majority of findings were from cross-sectional studies.

In six studies, household members did not enroll because they did not trust the insurer [42, 46, 52, 53, 55, 58] while in three studies, household members were more likely to enroll if the organization was financially trustworthy, honest, and transparent [31, 33, 59]. Compared to ex-members, current members were significantly more likely to place higher trust in scheme (*p* < 0.001) [45, 60] and scheme management (OR = 4.01) [58].

Households who had a sense of ownership of scheme were more likely to enroll [33, 47, 53, 58] and perceive the scheme as sustainable without it being forced on them [61]. Perceived financial risk protection associated with enrolling in a CBHI scheme also played an important role in individuals’ decisions to enroll or adhere to the scheme [47, 56, 62, 63]. Perceived quality of care was another widely reported factor affecting decisions to enroll [31, 42–45, 53, 58, 64] or drop out of a CBHI scheme [52, 65].

Consumer satisfaction with services provided by scheme positively influenced decisions to renew membership in a scheme [36, 38, 58, 66] while poor satisfaction with services motivated decisions to discontinue membership [65]. Reasons affecting enrollee satisfaction included staff skills, reimbursement rate, membership fee, and drug quality [52, 65].

##### Personal pre-disposition (*n* = 24)

Personal pre-dispositions such as previous experience with local groups, perceived affordability of care and health status influenced enrollment and sustainability of CBHI schemes.

Positive experience with other community associations was associated with increased enrollment in a CBHI scheme [32, 67], while negative experience contributed to low enrollment in a CBHI scheme [33, 43, 47]*.*

Four studies conducted in Nigeria, the Philippine, Rwanda, and Uganda cited perceived affordability of care as an important motive for enrollment or willingness to renew enrollment [33, 48, 52, 54].

Eighteen studies found that health status was associated with enrollment and renewal of enrollment in a CBHI scheme, pointing to adverse selection [11, 32, 34–40, 45, 56, 58, 65–70]. Presence of chronic illness or higher frequency of illness episodes within the past one to three months were significantly associated with enrollment [34, 36, 39, 40, 45, 58, 69, 70] while being healthy significantly decreased the probability of renewing membership [65]. For instance, in Senegal, member households were twice as likely to have had an illness, accident or injury (OR = 2), and were nearly twice as likely to have a disability, than ex-member households (OR = 1.74) [58], whereas in Burkina Faso, lower number of illness episodes in the past 3 months increased the probability that a household did not renew its membership in a CBHI scheme (OR = 0.87) [65]. Adverse selection mainly came from partially enrolled households [40] and from provision of premium subsidies to sick people, leading to insured groups having significantly higher percentage of sick individuals [11].

##### Socio-demographic factors (*n* = 20)

Age, gender, marital status, ethnicity, religion, area of residence, economic status, and level of education were found to be associated with enrollment in a CBHI scheme.

Quantitative studies suggested a positive correlation between older age (i.e., age 36 and above, on average) [34, 35, 39, 40, 43, 58, 65, 67, 71], being a female [39, 40, 45, 71], married [35, 39, 45, 68], employed [35, 36, 45, 66] and enrollment in a CBHI scheme. Conversely, individuals belonging to an ethnic minority [45, 49, 64] or migrating [39] were less likely to enroll in a CBHI scheme.

The results were mixed for religious affiliation [32, 65, 68, 70], occupational setting [43, 65], education [35, 36, 40, 43, 45, 64–68], per capita expenditure [58, 64, 65, 67], and economic status [11, 32–35, 39, 45, 56, 58, 64, 66–68, 70] and enrollment into a CBHI scheme.

#### 2. Interpersonal level

Emerging themes under this category included household dynamics, relative relations, and social solidarity (see Table 2 and Additional file 6).

**Table 2:** Summary of key findings under interpersonal level

Interpersonal-level factors	Number and type of studies ^a^	Reported as barriers	Reported as facilitators	Related to ^b^		
				Implementation	Uptake	Sustainability
Household size	N = 10 Qualitative (1); Cross-sectional (6); Mixed (2); Case study (1)	N = 6 [35, 54, 62, 64, 65, 67]	*N* = 4 [33, 40, 45, 70]		X	X
Household head characteristic	N = 6 RCT (1); Cross-sectional (5)	N = 3 [53, 64, 65]	N = 3 [33, 37, 72]		X	X
Peer influence	N = 4 Qualitative (1); Cross-sectional (1); Mixed (2)	–	N = 4 [36, 43, 45, 58]		X	X
Social solidarity	N = 8 Cross-sectional (3); Mixed (4); Case study (1)	N = 1 [58]	N = 7 [33, 41, 56, 63, 67, 68, 73]		X	

^a^ Some of the studies included both barriers and facilitators

^b^ X symbol denotes whether the factor relates to implementation, uptake or sustainability

##### Household dynamics (*n* = 13)

Findings from quantitative data indicate that household dynamics influenced decisions to enroll or renew enrollment in a CBHI scheme.

Six studies found that larger households were less likely to enroll in CBHI schemes [35, 54, 62, 64, 67] or drop out of the scheme [65] due to difficulties in meeting the subscription fees, while four studies found that individuals with a large family were more likely to be enrolled in a CBHI scheme [33, 40, 45, 70]. One of the studies attributed the latter to the possibility of signing up in a CBHI plan as a family of up to seven members for the same annual premium [33].

Six studies found that characteristics of a household head influenced enrollment [33, 37, 53, 64, 65, 72]. An educated household head was associated with increased enrollment [37, 65, 72] while having a young household head was associated with decreased membership overall [53, 64]. In three studies, male-headed households were more likely to enroll [33, 53, 65], while in one study, female-headed households were more likely to remain members of the scheme [72].

##### Peer influence (*n* = 4)

Persuasion by family, friends, or relatives was associated with enrollment [43] and sustainability [36]. Also, CBHI scheme members were more likely to have more close relatives and friends in the scheme (*p* < 0.001) [45] and to have heard of the scheme from a family member or friend compared to another source [58].

##### Social solidarity (*n* = 8)

Eight studies found that when community members felt a sense of solidarity, they were more likely to join the scheme [33, 41, 56, 58, 63, 67, 68, 73]. Merging individual associations, allowing payments in installments, taking local initiatives to help poor members, and promoting regularity of contributions helped ensure a higher value of solidarity, and thus more participation in the scheme [33, 41, 68, 73].

#### 3. Community level (*n* = 13)

Community-level factors included culture, community involvement in scheme implementation and management, and characteristics of CBHI districts (see Table 3 and Additional file 6).

**Table 3:** Summary of key findings under community level

Community-level factors	Number and type of studies ^a^	Reported as barriers	Reported as facilitators	Related to ^b^		
				Implementation	Uptake	Sustainability
Role of culture	N = 2 Cross-sectional (1); Qualitative (1)	N = 2 [53, 54]	–		X	
Community involvement	N = 11 Qualitative (5); Mixed (5); Case study (1)	*N* = 5 [47, 52, 54, 57, 68]	N = 6 [43, 48, 61, 62, 67, 74]	X	X	X
District-level characteristics	N = 1 Mixed (1)	–	N = 1 [45]			X

^a^ Some of the studies included both barriers and facilitators

^b^ X symbol denotes whether the factor relates to implementation, uptake or sustainability

##### Role of culture (*n* = 2)

Two studies conducted in Kenya and Uganda reported that cultural norms such as beliefs that enrollment invites illness, preference for unconventional medicine, and reliance on other means of financial transactions besides money hindered uptake of a CBHI scheme [53, 54].

##### Community involvement (*n* = 11)

Community involvement was reported to influence implementation, uptake and sustainability of CHBI schemes.

Five studies found that high community involvement in scheme development, implementation and promotion strategy was an enabler to enrollment in CBHI [43, 48, 62, 67, 74]. Furthermore, involvement of community heads and religious leaders helped tailoring services to needs, decreased complaints and eased implementation of CBHI schemes. Conversely, four studies found that low community participation resulted in decreased support for the scheme and a consequent decrease in enrollment [47, 52, 54, 68].

Two studies highlighted the crucial impact of community members’ involvement in planning and decision making on sustainability of scheme [57, 61]. It is believed that low community participation in decision-making resulted in a decrease in value placed on scheme and consequently, scheme membership.

##### Characteristics of CBHI scheme districts (n = 1)

One mixed-method study conducted in Lao People’s Democratic Republic examined the characteristics of districts with CBHI implementation and found that compared to non-CBHI districts, CBHI districts had a higher population density, lower poverty rates, higher literacy rates, and a higher proportion of the population working in the non-agricultural sector [45].

#### 4. Systems level: Governance arrangement

Emerging themes under this category included: stakeholder involvement; political economy context; government support; management/administrative structure; capacity of insurance promoters; package content; and membership criteria (see Table 4 and Additional file 6).

**Table 4:** Summary of key findings under governance arrangement level of health system

Systems-level factors: Governance arrangement	Number and type of studies ^a^	Reported as barriers	Reported as facilitators	Related to ^b^		
				Implementation	Uptake	Sustainability
Stakeholder involvement	N = 3 Qualitative (2); Case study (1)	N = 3 [46, 48, 62]	–	X		
Political economy context	N = 6 Qualitative (1); Mixed (3); Case studies (2)	N = 5 [31, 55, 59, 63, 75]	N = 1 [58]	X	X	X
Government support	N = 7 Qualitative (4); Mixed (2); Case study (1)	N = 2 [47, 75]	N = 5 [40, 48, 55, 61, 74]		X	X
Management and administrative structure	N = 12 Qualitative (3); Cross-sectional (2); Mixed (4); Case studies (3)	N = 8 [32, 44, 46, 48, 49, 62, 73, 83]	N = 5 [33, 41, 48, 55, 58]	X		X
Capacity of insurer promoters	N = 3 Mixed (2); Case study (1)	N = 2 [47, 48]	N = 1 [61]	X	X	X
Package content	N = 9 Qualitative (2); Cross-sectional (4); Mixed (2); Case study (1)	N = 5 [4, 48, 53–55, 61]	N = 5 [4, 33, 54, 56, 66]		X	
Membership size	*N* = 3 Qualitative (2); Crosss-sectional (1)	N = 2 [57, 61]	N = 1 [36]			X
Membership criteria	N = 10 Qualitative (4); Cross-sectional (3); Mixed (3)	N = 7 [40, 47, 52, 54, 61, 62, 76]	N = 5 [33, 61, 73, 76, 84]		X	X

^a^ Some of the studies included both barriers and facilitators

^b^ X symbol denotes whether the factor relates to implementation, uptake or sustainability

##### Stakeholder involvement (*n* = 3)

The involvement of health professionals and managers in scheme design was reported to influence the implementation process. In Tanzania, the introduction of CBHI scheme policy at central level with little input from district managers resulted in managers perceiving the implementation process as imposed and rushed with little time to prepare. Consequently, this undermined the attainment of scheme objectives [46]. In Guinea-Conakry, poor involvement of health professionals in scheme design contributed to low support for scheme implementation [62]. In Guatemala, the slow and problematic development of the scheme was influenced by conflict over health care provision by church-affiliated institutions [48].

##### Politico- economical context (*n* = 6)

Six studies showed that the political and economic context had some effect on uptake, implementation and sustainability of CBHI. In Senegal, members believed CBHI schemes were managed in a democratic manner, hence was correlated with increased enrollment [67]. In Ghana, Tanzania, and Zaire, the socio-economic turmoil had a negative effect on enrollment and funding of CBHI schemes [31, 55, 59]. In Nigeria, removal of the governor as a result of political tensions resulted in decreased state interest and support for the CBHI scheme [63]. In Uganda, CBHI scheme was perceived by district health officers and senior staff of the Ministry of Health as a controversial and politically sensitive issue, where user fees have been abolished in the public sector following a decision by the president [75].

##### Government support (*n* = 7)

Seven studies reported that government support, as in funding, legislative or technical, could have a positive influence on uptake [40, 47, 74] and in sustaining CBHI schemes [48, 55, 61, 75].

Three studies examined the role of government in influencing the uptake of a CBHI scheme. In China, local government paid full premium to those identified as poor in order to avoid their exclusion from the scheme [40]. In Rwanda, government support (through issuance of officially stamped scheme membership card in return for paying annual premium) was suggested by household members as the only way to enroll in the scheme [74]. In Uganda, the lack of a clear national policy and implementation guideline for the CHBI scheme resulted in low enrollment in the scheme [47].

Four studies examined the role of government in sustaining CBHI schemes. In Tanzania and Uganda, financial support from government was reported to have a positive influence on sustaining the CBHI scheme and sufficiently meeting the health needs of the communities [55, 61, 75]. In Guatemala and the Philippines, the establishment of an “umbrella organization” that can provide support in scheme design and training as well as involve government, non-government and academia in the development process was suggested by households as critical to promote sustainability of scheme [48].

##### Management/administrative structure (*n* = 12)

The management/administrative structure of CHBI schemes was reported to influence implementation and sustainability of CBHI schemes.

Four studies conducted in Cameroon, Ghana, Philippine, and Rwanda described establishing a robust administrative body in the initial phases of developing a CBHI scheme as essential to preventing unintended external interferences in the system and enabling a smooth implementation process [41, 48, 73, 74].

The structure of administrative body such as qualifications of scheme directors/managers (incorruptible, transparent, honest, and fair) [73, 74], well-built financial system [32, 44, 46, 57] and presence of women in scheme leadership [49] were reported to promote scheme sustainability and equity in scheme management. In Tanzania, embedding the management of scheme fund into the existing district health management arrangements controlled by government made it possible to jointly attain sustainability and assure “public accountability” [55]**.** While lack of financial accountability of managers decreased members’ trust in scheme and promoted decisions to discontinue membership [47, 58, 74].

##### Capacity of insurance promoters (*n* = 3)

Three studies reported that the capacity of insurance promoters influenced implementation, uptake and sustainability of a CBHI scheme.

In Guatemala, poor stakeholders’ capacity in making decisions regarding a viable CBHI contributed to the slow and problematic development of the scheme [48]. In Uganda, limited expertise within the ministry of health and among donors in setting up CBHI schemes was reported to lead to low uptake of the scheme [47] while good leadership that can support schemes to start income generation activities and attract more members was reported to promote sustainability of scheme [61].

##### Package content (*n* = 9)

The benefit packages covered by CBHI schemes were reported to influence uptake. Benefit packages that are tailored to the needs of a community [33, 48, 51, 53, 56, 66], are non-discriminatory [61], and cover outpatient services [4] increased enrollment in a CBHI scheme. On the other hand, packages with limited disease coverage contributed to low uptake [4, 54, 61].

##### Membership size (n = 3)

Three studies conducted in Uganda and Thailand reported that scheme sustainability depended on the size of its membership, with low enrollment and high dropout rate negatively affecting sustainability [36, 57, 61].

##### Membership criteria (*n* = 10)

Membership criteria was reported to influence uptake and sustainability of a CBHI scheme.

Five studies conducted in Nigeria, Thailand and Uganda reported that stringent membership criteria (e.g. only allowing families of 5 to enroll or requiring 60% of a community to enroll before providing services or insuring the whole household) limited some communities or community members from subscribing or renewing their subscriptions [52, 54, 61, 62, 66]. The 60% group membership requirement was specifically perceived by managers and community members as a serious barrier to overall scheme sustainability [61]. In contrast, four studies conducted in Burkina Faso, China, Ghana, and Senegal reported that compulsory or ‘household’ enrollment decreased adverse selection due to lower probability of having only sick individuals enrolled in the scheme [40, 68, 73, 76]. In Rwanda, the possibility of signing up in a CBHI plan as a family of up to seven members for the same annual premium served as an incentive for larger households to enroll [33].

#### 5. Systems level: Financial arrangement

Emerging themes under this category included: amount and timing of premium; cost-sharing; payment arrangement for services; and financial viability of scheme (see Table 5 and Additional file 6).

**Table 5:** Summary of key findings under financial arrangement level of health system

Systems-level factors: Financial arrangement	Number and type of studies ^a^	Reported as barriers	Reported as facilitators	Related to ^b^		
				Implementation	Uptake	Sustainability
Amount and timing of premium	N = 19 Qualitative (4); Cross-sectional (4); Mixed (8); Case studies (3)	N = 19 [38, 39, 42, 45–50, 52, 53, 55, 56, 59–62, 65, 76]	N = 4 [47, 50, 56, 76]		X	X
Cost-sharing	N = 10 Quantitative (1); Qualitative (2); Cross-sectional (6); Mixed (1)	N = 9 [4, 33, 36, 38, 39, 44, 56, 75, 77]	N = 1 [57]		X	X
Payment arrangement for services	N = 6 Cross-sectional (1); Qualitative (1); Mixed (3); Case study (1)	N = 4 [36, 38, 48, 78]	N = 2 [55, 75]	X		
Financial viability of scheme	N = 9 Qualitative (2); Cross-sectional (2); Mixed (4); Case study (1)	N = 7 [36, 38, 53, 61, 68, 73, 78]	N = 2 [55, 57]			X

^a^ Some of the studies included both barriers and facilitators

^b^ X symbol denotes whether the factor relates to implementation, uptake or sustainability

##### Amount and timing of premium (*n* = 19)

Amount and timing of premium collection was found to influence uptake and sustainability of a CBHI scheme.

Fourteen studies reported that high premium rates negatively influenced enrollment [38, 39, 42, 45, 46, 53, 55, 60, 62, 65, 76], and led to inequity in enrollment among the poor and most vulnerable in society [47, 52, 61]. Furthermore, applying uniform enrollment policies for all enrollees resulted in lower enrollment amongst the most vulnerable populations [49], whereas setting affordable contribution rates adjusted at reasonable intervals facilitated enrollment [48, 56].

Seven studies reported that the method and timing of premium collection influenced enrollment and dropout [47, 50, 55, 56, 59, 60, 76]. Specifically, modalities that require premium to be paid all at once for the entire household and individual-based premiums, were associated with low enrollment [55, 56, 59, 76] and high dropout [50]. Factors that facilitated enrollment included allowing members to make contributions in installments, linking premium payment to agricultural produce [47, 56], and establishing mutual cells for beneficiaries to encourage each other or act as pressure groups for group leaders to pay premiums [50, 76].

##### Cost-sharing (*n* = 10)

Cost-containment measures were reported to influence uptake and sustainability of CBHI schemes.

High co-payment rates were reported by household members to hinder individuals from joining health insurance schemes and contribute to insurance dropout [39, 44, 56, 75]. Similarly, ceilings and deductibles for reimbursement of inpatient services served as obstacles for poor families’ access to health care [4]. In India, out of pocket expenditure was mainly attributed to transport, medicine and pre-diagnostic investigations, highlighting the need for the scheme to improve strategic purchasing [77]. In Rwanda, out-of-pocket spending per episode of illness was influenced negatively if patients lived in the health center’s vicinity and if they owned cattle [33].

Nonetheless, the introduction of cost-containment measures was highlighted as necessary to reduce escalating cost of medical claims and decrease overutilization of services, which in turn could pose threats to the sustainability of CBHI schemes [36, 38, 57].

##### Payment arrangements for services (*n* = 6)

Provider payment method was reported to influence implementation of a CBHI scheme. In Tanzania, public and private health providers viewed the capitation payment associated with CBHI scheme as a potentially appealing alternative to collecting user fees, often at times when people were unable to pay [55]. Similarly, in the Philippines, the capitation agreement for hospital-based services was highlighted as one of the factors contributing to the success of the scheme [48]. However, in Burkina Faso, providers perceived the insufficient levels of capitation payments, the infrequent payment schedule, and the lack of a mechanism for reimbursing service fees (as opposed to only drugs) as significant sources of dissatisfaction and loss of motivation [78]. Similarly, delays in processing provider claims in Ghana [38], and insufficient reimbursement of expenses in Thailand [36] negatively influenced service delivery. In Uganda, the abolition of user fees in public sector gave rise to the practice of “under-the-table” payments, potentially impeding improvements in service delivery [75].

##### Financial viability of scheme (*n* = 9)

Financial viability of scheme was a critical issue highlighted in nine studies [36, 38, 53, 57, 61, 66, 68, 73, 78]. Factors reported as threat to financial viability and long-term sustainability included facility bankruptcy [78], operating on small budgets, small risk pool [57, 61], future rises in health care costs [73], irregularly of contributions [68], decreased contribution of informal sector [38], overutilization of services, and heavy reliance on external funding and donor subsidies to fund the running costs of a scheme [52, 57, 61]. In contrast, additional monies and local purchasing power for health were reported to potentially enhance sustainability [55].

#### 6. Systems level: Delivery arrangement

Emerging themes under this category included: human resource planning, human resource management; facility-related factors; accessibility of facilities; and marketing and promotion strategies (see Table 6 and Additional file 6).

**Table 6:** Summary of key findings under delivery arrangement level of health system

Systems-level factors: Delivery arrangement	Number and type of studies ^a^	Reported as barriers	Reported as facilitators	Related to ^b^		
				Implementation	Uptake	Sustainability
Human resource planning and management						
Human resource planning	N = 5 Qualitative (2); Mixed (2); Case studies (1)	N = 4 [46, 52, 63, 79]	N = 2 [52, 77]	X	X	X
Human resource management	*N* = 7 Qualitative (3); Cross-sectional (1); Mixed (2); Case studies (1)	N = 5 [45, 46, 52, 74, 78]	N = 2 [48, 62]	X	X	
Health facility-related factor						
Facility environment	N = 6 Qualitative (3); Cross-sectional (1); Mixed (2)	N = 4 [52, 54, 56, 74]	*N* = 2 [63, 77]		X	X
Supplies and materials	*N* = 11 Qualitative (4); Cross-sectional (2); Mixed (5)	N = 11 [42, 45, 46, 52, 54, 56, 62, 63, 74, 75, 78]	–	X	X	
Patient waiting time	N = 3 Qualitative (1); Mixed (2)	N = 3 [45, 52, 56]	–		X	X
Interpersonal skills	N = 7 Qualitative (3); Cross-sectional (2); Mixed (2)	N = 7 [45, 46, 52, 56, 61, 65, 74]	–		X	X
Accessibility of health facility						
Distance to facility	N = 17 Qualitative (9); Cross-sectional (2); Mixed (4); Case studies (2)	N = 11 [4, 31, 33, 38, 45, 46, 52, 53, 57, 77, 79]	N = 7 [32, 33, 35, 52, 58, 65, 66]		X	
Choice of facility	N = 3 Qualitative (3)	N = 2 [46, 76]	N = 1 [57]		X	X
Referral systems	N = 5 Qualitative (1); Cross-sectional (2); Case studies (2)	N = 3 [31, 36, 46]	N = 2 [48, 59]		X	X
Marketing and promotion strategies						
Adequacy of campaigns	N = 11 Qualitative (2); Cross-sectional (5); Mixed (3); Case study (1)	N = 8 [41, 43, 44, 54, 56, 58, 75, 77]	N = 3 [48, 57, 62]	X	X	X
Marketing technique	N = 4 Qualitative (1); Cross-sectional (1); Mixed (1); case study (1)	N = 2 [57, 79]	N = 2 [33, 43]		X	

^a^ Some of the studies included both barriers and facilitators

^b^ X symbol denotes whether the factor relates to implementation, uptake or sustainability

##### Human resource planning (*n* = 5)

Absence of health personnel at health care facility was reported to constrain scheme implementation [63], negatively influence enrollment [46, 79], and hinder willingness to renew enrollment [52]. Conversely, the availability of health care providers at health facility was reported to increase utilization [52] and enrollee satisfaction with CBHI schemes [77].

##### Human resource management (*n* = 7)

Management of health personnel was highlighted as another factor influencing uptake and implementation of a CBHI scheme.

In Lao People’s Democratic Republic, Nigeria, and Rwanda, provider incompetence created mistrust among beneficiaries and hindered enrollment in CBHI schemes [45, 52, 74].

In Tanzania, insufficient supervision by district managers raised community members’ concerns about improper provision of services by staff, including absenteeism during working hours [46]. In the Philippines, strong commitment of health workers contributed to proper implementation of the scheme [48]. In Burkina Faso, Guatemala and Zaire, the establishment of an incentive system for health workers was critical to enhance their commitment and support for CBHI [48, 62, 78].

##### Health facility-related factors (*n* = 14)

Facility environment, supplies and material, patient waiting time, and interpersonal skills were found to influence implementation, uptake and sustainability of a CBHI scheme.

In Kenya and Tanzania, corruption and conflict of interest at health facility affected decisions to enroll and contributed to insurance dropout [56]. In Nigeria, Rwanda and Uganda members and non-members of CBHI schemes complained about the inconvenient facility environment including lack of cleanliness and electricity, which affected enrollment decisions [52, 54, 74]. On the other hand, cleanliness and availability of good quality treatment enhanced enrollment in a CBHI scheme in Nigeria [63] and beneficiary satisfaction in India [77].

Lack of drugs and other essential medical supplies was highlighted by service managers and providers to impede their ability to fulfill their professional roles and responsibilities [52, 75, 78]. Furthermore, inadequate ward facilities, laboratory and diagnostic equipment, and essential drugs was reported by household members to contribute to perceived low quality of services and low enrollment in scheme [42, 45, 46, 52, 54, 56, 62, 75].

Patient waiting time at health facility was reported by household members to hinder individuals from joining the scheme [56] and contribute to insurance dropout [45, 52, 56].

Lack of interpersonal skills, in terms of poor hospitality and rude staff behavior, was also highlighted by household members to affect enrollment decisions [45, 46, 52, 56, 61, 74] as well as decisions to discontinue scheme membership [65]. Discrimination against scheme members was another raised concern which hindered people from joining health insurance schemes and contributed to insurance dropout [45, 56, 61].

##### Accessibility of health facility (*n* = 21)

Accessibility of health care facility, in terms of travel distance to facility (*n* = 17), choice of facility (*n* = 3), and referral systems (*n* = 5) was found to influence uptake and sustainability of the scheme. The majority of findings were from cross-sectional studies.

Seventeen studies, mainly cross-sectional, found that distance to health facility influenced enrollment in a CBHI scheme [4, 32, 33, 35, 38, 44–46, 52, 53, 57, 58, 66, 73, 77, 79] as well as drop out of the scheme [65]. For instance, in Senegal, members were more than twice as closer to health service providers (OR = 2.25) and three times more likely to report that health care access is an advantage of membership (OR = 3.05) [58]. Similarly, in Burkina Faso, shorter distance to health facility contributed to lower dropouts (OR = 0.36; *p* = 0.05) [65].

Two studies reported that restrictions on choice of health facility negatively influenced enrollment [46, 76]. A third study highlighted expanding the pool of affiliated providers so that members can obtain outpatient care at clinics closer to their homes, as a good model for sustainability of the scheme [57].

Five studies reported that referral systems influenced the uptake and sustainability of CBHI schemes [31, 36, 59]. In Tanzania, lack of referral systems was reported by district and community respondent groups to contribute to low perceived quality of care and consequently, low enrollment in the scheme [46]. In Ghana and Thailand, the absence of referral systems between the different levels of care was reported to influence the sustainability of the scheme [31, 36], while in Zaire and the Philippines the presence of a strong referral process helped offset inappropriate hospital utilization and contributed to the sustainability of the schemes [48, 59].

##### Marketing and promotion strategies (*n* = 13)

Marketing and promotion strategies were reported to influence implementation and uptake of a CBHI scheme.

Provision of limited information on the availability of scheme and poor sensitization on the core principles of CBHI negatively affected consumer awareness of scheme [41, 43, 54, 56], enrollment rates [43, 57, 58, 79], utilization [77] and satisfaction with services provided by the scheme [44]. In Uganda, the absence of a national conference to promote CBHI contributed to the low level of knowledge of the scheme by MOH staff, district managers, and health professionals [75]. In contrast, intensive awareness and information campaigns at community level resulted in increased enrollment [33, 48] and adherence to scheme [62]. Furthermore, ensuring proper rural-urban coverage of campaigns was highlighted by community members to play an important role in increasing awareness about the scheme [56]. The intensity of exposure to campaign channels (e.g. radio, television, interpersonal) was also found to influence enrollment [33, 43, 57, 79]. Specifically, respondents with access to radio or to two or more campaign channels were significantly more likely to enroll in the scheme [33, 43].

## Discussion

We identified 51 studies reporting on a range of barriers and facilitators to the implementation, uptake and sustainability of CBHI schemes across 22 countries. Many of the studies failed to meet methodological safeguards for protecting from bias, thus the findings should be interpreted with caution. Given the heterogeneity in quantitative study design and outcome measures, we could not conduct meta-analyses. Thus, we synthesized the findings narratively, and categorized according to the ecological model.

Although CBHI schemes have evolved rapidly in LMIC countries, many of these continue to be challenged by low uptake, coverage and sustainability. As evident from the findings of this review, there are a multitude of interrelated factors at the individual, interpersonal, community and systems level that drive the implementation and sustainability of CBHI schemes. These should be properly addressed in scheme design and implementation and harmonized across different levels of the ecological model to ensure proper attainment of scheme objectives and promote effective and equitable health systems. An overview of the factors influencing implementation, uptake and sustainability of CBHI schemes is presented in Fig. 2.

Two previously published systematic reviews focused on factors influencing CBHI enrollment: Bhageerathy et al. looked at the enrollment process, CBHI models, and health care seeking behavior in South Asia [18], while Adebayo et al. focused on a subset of outcomes, specifically uptake of or willingness to pay for CBHI schemes in LMICs [19]. Our systematic review provides a much more global perspective than the South Asian alone as well as attempts to identify all factors influencing implementation, enrollment, and sustainability of *already* implemented CBHI schemes. Furthermore, we provide a conceptual framework of factors critical to the implementation, uptake and sustainability of CBHI schemes.

All three reviews pointed to the importance of involving the community in scheme development and implementation to increase enrollment and sustainability of schemes. In addition, they indicated that engaging the community in decision-making about the types of services, payment approach and service delivery increased satisfaction with services as these were tailored to the community needs. Our findings were also consistent with those by Adebayo et al. in terms of the negative influence of poor perceived quality of care, lack of trust, and lack of financial resources on CBHI uptake. However, unlike that review, we found a consistently negative correlation between long distance to health facility and enrollment or renewal of scheme membership (as reported in 17 studies). This could reflect the method used by Adebayo et al., whereby ‘willingness to pay’ was taken as a proxy indicator to enrollment. One critical area not covered by the findings of the two previous reviews was the role of government in CBHI schemes. Our review highlighted the important role of government in establishing the necessary legislative, technical and regulative support to ensure sustainability of CBHI schemes. Further, having a transparent, incorruptible, and honest governance were perceived as essential for trusting the scheme.

### Implications for policy and practice

Policymakers and stakeholders interested in implementing CBHI schemes should first assess the specific characteristics and preferences of the community, including the approach to solidarity in the target population [48]. This should be coupled with awareness and information campaigns on insurance concepts in general, and CBHI schemes in particular, to inform individuals about the scheme and promote its uptake. Policymakers and stakeholders could also consider creating opportunities for active participation of community members to enhance trust, accountability, and enrollment in scheme.

Implementation of CBHI schemes should go hand in hand with ensuring the necessary institutional and regulatory environment to steer health care providers’ behaviors. It is important for policymakers and stakeholders to consider how the current payment methods of CBHI schemes influence provider performance, and how changes in the methods could improve performance and support for the scheme [80]. Further, strengthening policymaker-implementer relations and promoting a common language across stakeholders could help minimize conflicts and facilitate the implementation process.

Policymakers and stakeholders should also invest in efforts to address potential inequities that may arise with CBHI schemes, specifically in terms of enrollment and access to services. Possible policy options include: exempting the poor and most vulnerable populations from premium payment; providing premium subsidies; differentiating contributions according to socio-economic groups; adjusting contribution rates to reflect changes in benefits, health costs and inflations; and making the timing and modalities of premium collection flexible and tailored to the context. Furthermore, addressing geographical coverage of health facilities in scheme design and implementation is critical given its central role in determining people’s access to care.

To enhance sustainability of CBHI schemes, it would be important to balance strategies promoting enrollment and access, with strategies that could help minimize adverse selection and moral hazards typically associated with CBHI schemes. Policy options include using ‘household’ as the unit of enrollment, defining a minimum percentage of individuals that would be required before providing insurance, imposing a waiting period before services could be utilized, or establishing strong referral systems across the different levels of care. Whatever mechanism is selected, it is important to ensure that it is flexible, adapted to reality, and clearly defined in order to avoid deterring individuals from enrolling.

Finally, if CBHI schemes are to contribute to UHC, it would be critical to involve the government to provide the necessary legislative, technical, financial, and regulative support to implement CBHI schemes. Establishing a policy framework could help legitimize the CBHI scheme and position it within the context of national health financing systems. Consideration could also be given to establishing an “umbrella organization” that would provide support in design, training and information services as well as involve government, non-government and academia, as an integral part of the development and implementation process [48]. This is especially relevant in light of a resurgence in discussions about universal health coverage as a key component of health-related Sustainable Development Goals [81].

### Strengths and limitations

Strengths of our methodology include pre-publishing a protocol, using rigorous and transparent process, and following standard methods for reporting systematic reviews [82]. In addition, we conducted a comprehensive search of the published and grey literature to avoid potential publication bias. Furthermore, the inclusion of all types of study design allowed for a more comprehensive understanding of the issue at hand [21].

This review has several limitations. First, we acknowledge that there may be some areas of overlap in the categorization of themes according to the ecological model. Moreover, despite our attempt to report the findings by implementation, uptake, and sustainability, it is important to note that their interrelatedness brought up a few challenges. For instance, uptake was also reported to influence sustainability of the scheme in few studies. Also, and in few cases, the distinction between implementation and sustainability of scheme was not very clear. However, we attempted to minimize this through continuous input from team members with subject expertise on coding decisions and characterization of emerging themes. Second, our findings may be more generalizable to low-income countries, which were the focus of 35 (out of 51) studies. Third, we only included studies conducted in English, thus we may have missed out on relevant studies published in other languages. Also, despite our attempt to search the grey literature, we may have still missed potentially relevant studies published in other donor and governmental websites beyond the ones searched for this review. A final limitation is that the review does not incorporate studies that could have been published after the date of our search. However, it is unlikely that such studies would change the findings in a significant way.

## Conclusion

There are a multitude of interrelated factors at the individual, interpersonal, community and systems levels that drive the implementation and sustainability of CBHI schemes. These should be properly addressed in scheme design and implementation and harmonized across the different levels to ensure attainment of scheme objectives. Future research efforts should be directed towards conducting well-designed primary studies with particular attention to recruitment strategy, use of validated tools, and control for potential confounding variables. Furthermore, more research is needed on how CBHI schemes could complement the broader health financing system to progress to UHC.

## Additional files

Additional file 1: Search strategy (PDF 163 kb)

Additional file 2: List of coding themes corresponding to each level of the ecological model (PDF 195 kb)

Additional file 3: List of the excluded studies with reasons for exclusion. (PDF 381 kb)

Additional file 4: Overview of the characteristics of each included study (PDF 1046 kb)

Additional file 5: Quality appraisal of included studies (PDF 697 kb)

Additional file 6: Detailed findings of studies at the individual, interpersonal, community and systems level (PDF 635 kb)

## Abbreviations

CBHI: Community-based health insurance
LMIC: Low- and middle-income countries
UHC: Universal health coverage
