# Supplementary material for: Barriers and facilitators to implementation, uptake and sustainability of community-based health insurance schemes in low- and middle-income countries: a systematic review
Source: Int J Equity Health. 2018 Jan 29;17:13. doi: 10.1186/s12939-018-0721-4 (PMC5789675; doi:10.1186/s12939-018-0721-4)
Supplement: Supplementary file 1 — Search strategy (PDF 163 kb) [file 12939_2018_721_MOESM1_ESM.pdf]

Supplementary file 1: Search Strategy

**Database**	**Number of hits**
Medline	4007
EMBASE	5480
PubMed	3636
Global Health Library	2387
**Total**	**15, 510**

Database: EMBASE <1980 to 2014 Week 48>

Search Strategy: 5480

----

1 exp developing country/ (76655)

2 (Afghanistan or Albania or Algeria or Angola or Antigua or Barbuda or Argentina or Armenia or Armenian or Aruba or Azerbaijan or Bahrain or Bangladesh or Barbados or Benin or Byelarus or Byelorussian or Belarus or Belorussian or Belorussia or Belize or Bhutan or Bolivia or Bosnia or Herzegovina or Hercegovina or Botswana or Brasil or Brazil or Bulgaria or Burkina Faso or Burkina Fasso or Upper Volta or Burundi or Urundi or Cambodia or Khmer Republic or Kampuchea or Cameroon or Cameroons or Cameron or Camerons or Cape Verde or Central African Republic or Chad or Chile or China or Colombia or Comoros or Comoro Islands or Comores or Mayotte or Congo or Zaire or Costa Rica or Cote d'Ivoire or Ivory Coast or Croatia or Cuba or Cyprus or Czechoslovakia or Czech Republic or Slovakia or Slovak Republic or Djibouti or French Somaliland or Dominica or Dominican Republic or East Timor or East Timur or Timor Leste or Ecuador or Egypt or United Arab Republic or El Salvador or Eritrea or Estonia or Ethiopia or Fiji or Gabon or Gabonese Republic or Gambia or Gaza or Georgia Republic or Georgian Republic or Ghana or Gold Coast or Greece or Grenada or Guatemala or Guinea or Guam or Guiana or Guyana or Haiti or Honduras or Hungary or India or Maldives or Indonesia or Iran or Iraq or Isle of Man or Jamaica or Jordan or Kazakhstan or Kazakh or Kenya or Kiribati or Korea or Kosovo or Kyrgyzstan or Kirghizia or Kyrgyz Republic or Kirghiz or Kirgizstan or Lao PDR or Laos or Latvia or Lebanon or Lesotho or Basutoland or Liberia or Libya or Lithuania or Macedonia or Madagascar or Malagasy Republic or Malaysia or Malaya or Malay or Sabah or Sarawak or Malawi or Nyasaland or Mali or Malta or Marshall Islands or Mauritania or Mauritius or Agalega Islands or Mexico or Micronesia or Middle East or Moldova or Moldovia or Moldovian or Mongolia or Montenegro or Morocco or Ifni or Mozambique or Myanmar or Myanma or Burma or Namibia or Nepal or Netherlands Antilles or New Caledonia or Nicaragua or Niger or Nigeria or Northern Mariana Islands or Oman or Muscat or Pakistan or Palau or Palestine or Panama or Paraguay or Peru or Philippines or Philipines or Phillipines or Phillippines or Poland or Portugal or Puerto Rico or Romania or Rumania or Roumania or Russia or Russian or Rwanda or Ruanda or Saint Kitts or St Kitts or Nevis or Saint Lucia or St Lucia or Saint Vincent or St Vincent or Grenadines or Samoa or Samoan Islands or Navigator Island or Navigator Islands or Sao Tome or Saudi Arabia or Senegal or Serbia or Montenegro or Seychelles or Sierra Leone or Slovenia or Sri Lanka or Ceylon or Solomon Islands or Somalia or South Africa or Sudan or Suriname or Surinam or Swaziland or Syria or Tajikistan or Tadzhikistan or Tadjikistan or Tadzhik or Tanzania or Thailand or Togo or Togolese Republic or Tonga or Trinidad or Tobago or Tunisia or Turkey or Turkmenistan or Turkmen or Uganda or Ukraine or Uruguay or USSR or Soviet Union or Union of Soviet Socialist Republics or Uzbekistan or Uzbek or Vanuatu or New Hebrides or Venezuela or

Vietnam or Viet Nam or West Bank or Yemen or Yugoslavia or Zambia or Zimbabwe or Rhodesia).ti,ab. (897117)

3 lowest income group/ (15527)

4 (low* adj (gdp or gnp or gross domestic or gross national)).ti,ab. (194)

5 (low adj3 middle adj3 countr*).ti,ab. (4423)

6 (lmic or lmics or third world or lami countr*).ti,ab. (3852)

7 ((developing or less* developed or least developed or under developed or underdeveloped or middle income or low* income or underserved or under served or deprived or poor* or transition*) adj2 (countr* or nation? or population? or world or econom*)).ti,ab. (76227)

8 or/1-7 (988918)

9 (micro adj insurance).ti,ab,sh. (9)

10 (Communit* adj3 insurance*).ti,ab,sh. (1299)

11 ((health or insurance*) adj3 (benefit* or package* or scheme* or coverage*)).ti,ab,sh. (32385)

12 (((comprehensive* or community) adj health) and (insurance* or benefit* or scheme* or package* or coverage*)).ti,ab,sh. (3944)

13 ((consumer adj3 participat*) and (insurance* or ((health adj3 benefit*) or scheme* or package*))).ti,ab,sh. (23)

14 ((prepaid or (pre adj paid)) adj2 health adj2 (plan* or coverage* or insurance* or package* or scheme*)).ti,ab,sh. (315)

15 (universal adj3 coverage*).ti,ab,sh. (1803)

16 insurance benefits/ or insurance coverage/ or insurance, health/ or prepaid health plans/ or single-payer system/ (126355)

17 or/9-16 (153241)

18 (implement* or facilitat* or barrier* or challeng* or weakness* or obstacle* or hurdle* or limitation* or enabl* or resist* or constraint* or promot* or applica* or integrat* or utiliz* or utilis* or success* or lesson* or experience* or scale-up or roll-out).ti,ab,sh. (5758550)

19 8 and 17 and 18 (5995)

20 limit 19 to (english language and yr="1992 -Current") (5480)

****

Database: Ovid MEDLINE(R) without Revisions <1996 to January Week 1 2015>

Search Strategy: 4007

----

1 Developing Countries/ (34085)

2 Medically Underserved Area/ (4330)

3 (low* adj (gdp or gnp or gross domestic or gross national)).ti,ab. (128)

4 (low adj3 middle adj3 countr*).ti,ab. (2930)

5 (lmic or lmics or third world or lami countr*).ti,ab. (1380)

6 exp africa/ or exp central america/ or exp latin america/ or exp south america/ or exp asia/ or exp eastern europe/ (627186)

7 (Afghanistan or Albania or Algeria or Angola or Antigua or Barbuda or Argentina or Armenia or Armenian or Aruba or Azerbaijan or Bahrain or Bangladesh or Barbados or Benin or Byelarus or Byelorussian or Belarus or Belorussian or Belorussia or Belize or Bhutan or Bolivia or Bosnia or Herzegovina or Hercegovina or Botswana or Brasil or Brazil or Bulgaria or Burkina Faso or Burkina Fasso or Upper Volta or Burundi or Urundi or Cambodia or Khmer Republic or Kampuchea or Cameroon or Cameroons or Cameron or Camerons or Cape Verde or Central African Republic or Chad or Chile or China or Colombia or Comoros or Comoro Islands or Comores or Mayotte or Congo or Zaire or Costa Rica or Cote d'Ivoire or Ivory Coast or Croatia or Cuba or Cyprus or Czechoslovakia or Czech Republic or Slovakia or Slovak Republic or Djibouti or French Somaliland or Dominica or Dominican Republic or East Timor or East Timur or Timor Leste or Ecuador or Egypt or United Arab Republic or El Salvador or Eritrea or Estonia or Ethiopia or Fiji or Gabon or Gabonese Republic or Gambia or Gaza or Georgia Republic or Georgian Republic or Ghana or Gold Coast or Greece or Grenada or Guatemala or Guinea or Guam or Guiana or Guyana or Haiti or Honduras or Hungary or India or Maldives or Indonesia or Iran or Iraq or Isle of Man or Jamaica or Jordan or Kazakhstan or Kazakh or Kenya or Kiribati or Korea or Kosovo or Kyrgyzstan or Kirghizia or Kyrgyz Republic or Kirghiz or Kirgizstan or Lao PDR or Laos or Latvia or Lebanon or Lesotho or Basutoland or Liberia or Libya or Lithuania or Macedonia or Madagascar or Malagasy Republic or Malaysia or Malaya or Malay or Sabah or Sarawak or Malawi or Nyasaland or Mali or Malta or Marshall Islands or Mauritania or Mauritius or Agalega Islands or Mexico or Micronesia or Middle East or Moldova or Moldovia or Moldovian or Mongolia or Montenegro or Morocco or Ifni or Mozambique or Myanmar or Myanma or Burma or Namibia or Nepal or Netherlands Antilles or New Caledonia or Nicaragua or Niger or Nigeria or Northern Mariana Islands or Oman or Muscat or Pakistan or Palau or Palestine or Panama or Paraguay or Peru or Philippines or Philipines or Phillipines or Phillippines or Poland or Portugal or Puerto Rico or Romania or Rumania or Roumania or Russia or Russian or Rwanda or Ruanda or Saint Kitts or St Kitts or Nevis or Saint Lucia or St Lucia or Saint Vincent or St Vincent or Grenadines or Samoa or Samoan Islands or Navigator Island or Navigator Islands or Sao Tome

or Saudi Arabia or Senegal or Serbia or Montenegro or Seychelles or Sierra Leone or Slovenia or Sri Lanka or Ceylon or Solomon Islands or Somalia or South Africa or Sudan or Suriname or Surinam or Swaziland or Syria or Tajikistan or Tadzhikistan or Tadjikistan or Tadzhik or Tanzania or Thailand or Togo or Togolese Republic or Tonga or Trinidad or Tobago or Tunisia or Turkey or Turkmenistan or Turkmen or Uganda or Ukraine or Uruguay or USSR or Soviet Union or Union of Soviet Socialist Republics or Uzbekistan or Uzbek or Vanuatu or New Hebrides or Venezuela or Vietnam or Viet Nam or West Bank or Yemen or Yugoslavia or Zambia or Zimbabwe or Rhodesia).hw,ti,ab. (680493)

8 ((developing or least developed or least-developed or less* developed or under developed or under-developed or underdeveloped or middle income or middle-income or low* income or underserved or under served or deprived or poor* or transition*) adj2 (countr* or nation? or population? or world or econom*)).ti,ab. (44780)

9 or/1-8 (847372)

10 (micro adj insurance).ti,ab,sh. (7)

11 (Communit* adj3 insurance*).ti,ab,sh. (970)

12 Consumer Participation/ec, ut [Economics, Utilization] (296)

13 Comprehensive Health Care/ec, mt, og, ut [Economics, Methods, Organization & Administration, Utilization] (831)

14 exp Community Networks/ec, ut [Economics, Utilization] (607)

15 (insurance* or (health adj3 (benefit* or scheme* or package* or coverage*))).ti,ab,sh. (75167)

16 12 or 13 or 14 (1729)

17 15 and 16 (213)

18 ((health or insurance*) adj3 (benefit* or package* or scheme* or coverage*)).ti,ab,sh. (20141)

19 (((comprehensive* or community) adj2 health) and (insurance* or benefit* or scheme* or package* or coverage*)).ti,ab,sh. (2468)

20 ((consumer adj3 participat*) and (insurance* or ((health adj3 benefit*) or scheme* or package*))).ti,ab,sh. (16)

21 ((Health adj3 insurance*) not (database* or claim*)).ti,ab,sh. (13604)

22 insurance benefits/ or insurance coverage/ or universal coverage/ or insurance, health/ or insurance, hospitalization/ or prepaid health plans/ or single-payer system/ or state medicine/ or Insurance, Health, Reimbursement/ (58446)

23 ((prepaid or (pre adj paid)) adj2 health adj2 (plan* or coverage* or insurance* or package* or scheme*)).ti,ab,sh. (102)

24 Universal Coverage/ (2052)

25 (universal adj3 coverage*).ti,ab,sh. (1128)

26 24 or 25 (2662)

27 Health Services Accessibility/ (43345)

28 15 and 27 (6243)

29 10 or 11 or 17 or 18 or 19 or 20 or 21 or 22 or 23 or 26 or 28 (85358)

30 Health Plan Implementation/ (3577)

31 (implement* or facilitat* or barrier* or challeng* or weakness* or obstacle* or hurdle* or limitation* or enabl* or resist* or constraint* or promot* or applica* or integrat* or utiliz* or utilis* or success* or lesson* or experienc* or scale-up or roll-out).ti,ab,sh. (3190866)

32 30 or 31 (3191676)

33 9 and 29 and 32 (4569)

34 limit 33 to (english language and yr="1992 -Current") (4029)

35 34 not (animals/ not (animals/ and humans/)) (4007)

36 (universal adj3 coverage*).ti,ab,sh. (1128)

37 24 or 25 (2662)

38 Health Services Accessibility/ (43345)

39 15 and 27 (6243)

40 10 or 11 or 17 or 18 or 19 or 20 or 21 or 22 or 23 or 26 or 28 (85358)

41 Health Plan Implementation/ (3577)

42 (implement* or facilitat* or barrier* or challeng* or opportunit* or strength* or weakness* or obstacle* or hurdle* or limitation* or enabl* or resist* or constraint* or promot* or applicat* or integrat* or utiliz* or utilis* or harmonization* or consideration* or adopt* or success* or failure* or accept* or feasib* or enhanc* or lesson* or experienc* or (scal* adj up) or (roll* adj out) or sustainab* or uptak* or up-tak*).ti,ab,sh. (4166261)

43 30 or 31 (3191676)

44 9 and 29 and 32 (4569)

45 limit 33 to (english language and yr="1992 -Current") (4029)

46 34 not (animals/ not (animals/ and humans/)) (4007)

Database: PubMed < December Week 3 2014>

Search Strategy: 3636

Search	Query	Items found
#25	Search #5 AND #19 AND #22 Filters: Publication date from 1992/01/01 to 2014/12/31; Humans	3636
#24	Search #5 AND #19 AND #22 Filters: Publication date from 1992/01/01 to 2014/12/31	4567
#22	Search #20 OR #21	4947162
#21	Search "Health Plan Implementation"[Mesh]	3927
#20	Search ((((implement*[tiab] OR facilitat*[tiab] OR barrier*[tiab] OR challeng*[tiab] OR weakness*[tiab] OR obstacle*[tiab] OR hurdle*[tiab] OR limitation*[tiab] OR enabl*[tiab] OR resist*[tiab] OR constraint*[tiab] OR promot*[tiab] OR applica*[tiab] OR integrat*[tiab] OR utiliz*[tiab] OR utilis*[tiab] OR success*[tiab] OR lesson*[tiab] OR experienc*[tiab] OR scale-up[tiab] OR scaling-up[tiab] OR roll-out[tiab] OR rolling-out[tiab] OR rollout*[tiab])))	4946223
#19	Search (#7 OR #8 OR #9 OR #11 OR #12 OR #13 OR #14 OR #15 OR #16 OR #17 OR #18)	87180
#18	Search (health benefit*[tiab] OR health package*[tiab] OR health scheme*[tiab] OR health coverage*[tiab] OR insurance benefit*[tiab] OR insurance package*[tiab] OR insurance scheme*[tiab] OR insurance coverage*[tiab]))	19742
#17	Search ((comprehensive insurance*[tiab] OR comprehensive benefit*[tiab] OR comprehensive scheme*[tiab] OR comprehensive package*[tiab] OR comprehensive coverage*[tiab] OR community insurance*[tiab] OR community benefit*[tiab] OR community scheme*[tiab] OR community package*[tiab] OR community coverage*[tiab]))	3488
#16	Search ((Consumer participation insurance*[tiab] OR consumer participation benefit*[tiab] OR consumer participation scheme*[tiab] OR consumer participation package*[tiab]))	986
#15	Search ((micro-insurance*[tiab] OR microinsurance*[tiab] OR community insurance*[tiab]))	21
#14	Search ((Pre-paid health plan*[tiab] OR prepaid health plan*[tiab] OR pre-paid health coverage*[tiab] OR prepaid health coverage*[tiab] OR pre-paid health insurance*[tiab] OR prepaid health insurance*[tiab] OR pre-paid health package*[tiab] OR prepaid health package*[tiab] OR pre-paid health scheme*[tiab] OR prepaid health scheme*[tiab]))	245
#13	Search ((((("Insurance Benefits"[Mesh]) OR "Insurance Coverage"[Mesh])) OR Insurance Benefit*[Title/Abstract] OR Insurance Coverage*[Title/Abstract]))	17217
#12	Search universal coverage[MeSH Terms]	2065

Search	Query	Items found
#11	Search **(((((Hospitalization insurance*[Title/Abstract] OR Not-For-Profit Insurance*[Title/Abstract] OR Prepaid Health Plan*[Title/Abstract] OR Single-Payer System*[Title/Abstract] OR Universal Coverage*[Title/Abstract]))))**	1366
#10	Search **(((((Hospitalization insurance*[Title/Abstract] OR Not-For-Profit Insurance*[Title/Abstract] OR Prepaid Health Plan*[Title/Abstract] OR Single-Payer System*[Title/Abstract] OR Universal Coverage*[Title/Abstract] OR Community Health Planning*[Title/Abstract] OR State Health Plan*[Title/Abstract]))))**	1526
#9	Search **health insurance reimburse*[tiab]**	77
#8	Search **health insurance reimbursement[MeSH Terms]**	38658
#7	Search **((((((((((("Insurance, Hospitalization"[Mesh])) OR "Not-For-Profit Insurance Plans"[Mesh]) OR "Prepaid Health Plans"[Mesh]) OR "Single-Payer System"[Mesh]) OR "Insurance, Major Medical"[Mesh]) OR "Universal Coverage"[Mesh])))**	19403
#5	Search **#1 OR #2 OR #3 OR #4**	1522348
#4	Search **(((("Developing Countries"[Mesh]) OR "Medically Underserved Area"[Mesh])))**	67606
#3	Search **((developing[tiab] OR less developed*[tiab] OR under developed*[tiab] OR underdeveloped[tiab] OR least developed*[tiab] OR middle income*[tiab] OR low income*[tiab] OR under served*[tiab] OR underserved[tiab] OR deprived*[tiab] OR poor*[tiab] OR transition*[tiab] OR third world*[tiab]) AND (countr*[tiab] OR nation*[tiab] OR population*[tiab] OR world*[tiab] OR econom*[tiab])))**	241381
#1	Search **(((Afghanistan[tiab] OR Albania[tiab] OR Algeria[tiab] OR Angola[tiab] OR Antigua[tiab] OR Barbuda[tiab] OR Argentina[tiab] OR Armenia[tiab] OR Armenian[tiab] OR Aruba[tiab] OR Azerbaijan[tiab] OR Bahrain[tiab] OR Bangladesh[tiab] OR Barbados[tiab] OR Benin[tiab] OR Byelarus[tiab] OR Byelorussian[tiab] OR Belarus[tiab] OR Belorussian[tiab] OR BelORussia[tiab] OR Belize[tiab] OR Bhutan[tiab] OR Bolivia[tiab] OR Bosnia[tiab] OR Herzegovina[tiab] OR Hercegovina[tiab] OR Botswana[tiab] OR Brasil[tiab] OR Brazil[tiab] OR Bulgaria[tiab] OR Burkina Faso[tiab] OR Burkina Fasso[tiab] OR Upper Volta OR Burundi[tiab] OR Urundi[tiab] OR Cambodia[tiab] OR Khmer Republic[tiab] OR Kampuchea[tiab] OR Cameroon[tiab] OR Cameroons[tiab] OR Cameron[tiab] OR Camerons[tiab] OR Cape Verde[tiab] OR Central African Republic[tiab] OR Chad[tiab] OR Chile[tiab] OR China[tiab] OR Colombia[tiab] OR Comoros[tiab] OR Comoro Islands[tiab] OR Comores[tiab] OR Mayotte[tiab] OR Congo[tiab] OR Zaire[tiab] OR Costa Rica[tiab] OR Cote d'Ivoire[tiab] OR Ivory Coast[tiab] OR Croatia[tiab] OR Cuba[tiab] OR Cyprus[tiab] OR Czechoslovakia[tiab] OR Czech Republic[tiab] OR Slovakia[tiab] OR Slovak Republic[tiab] OR Djibouti[tiab] OR French Somaliland[tiab] OR Dominica[tiab] OR Dominican Republic[tiab] OR East Timor[tiab] OR East Timur[tiab] OR Timor Leste[tiab] OR Ecuador[tiab] OR Egypt[tiab] OR United Arab Republic[tiab] OR El Salvador[tiab] OR Eritrea[tiab] OR Estonia[tiab] OR Ethiopia[tiab] OR Fiji[tiab] OR**	781567

Search	Query	Items found
	**Gabon[tiab] OR Gabonese Republic[tiab] OR Gambia[tiab] OR Gaza[tiab] OR Georgia Republic[tiab] OR Georgian Republic[tiab] OR Ghana[tiab] OR Gold Coast[tiab] OR Greece[tiab] OR Grenada[tiab] OR Guatemala[tiab] OR Guinea[tiab] OR Guam[tiab] OR Guiana[tiab] OR Guyana[tiab] OR Haiti[tiab] OR Honduras[tiab] OR Hungary[tiab] OR India[tiab] OR Maldives[tiab] OR Indonesia[tiab] OR Iran[tiab] OR Iraq[tiab] OR Isle of Man[tiab] OR Jamaica[tiab] OR Jordan[tiab] OR Kazakhstan[tiab] OR Kazakh[tiab] OR Kenya[tiab] OR Kiribati[tiab] OR Korea[tiab] OR Kosovo[tiab] OR Kyrgyzstan[tiab] OR Kirghizia[tiab] OR Kyrgyz Republic[tiab] OR Kirghiz[tiab] OR Kirgizstan[tiab] OR Lao PDR[tiab] OR Laos[tiab] OR Latvia[tiab] OR Lebanon[tiab] OR Lesotho[tiab] OR Basutoland[tiab] OR Liberia[tiab] OR Libya[tiab] OR Lithuania[tiab] OR Macedonia [tiab] OR Madagascar[tiab] OR Malagasy Republic[tiab] OR Malaysia[tiab] OR Malaya[tiab] OR Malay[tiab] OR Sabah[tiab] OR Sarawak[tiab] OR Malawi[tiab] OR Nyasaland[tiab] OR Mali[tiab] OR Malta[tiab] OR Marshall Islands[tiab] OR Mauritania[tiab] OR Mauritius[tiab] OR Agalega Islands[tiab] OR Mexico[tiab] OR Micronesia[tiab] OR Middle East[tiab] OR Moldova[tiab] OR Moldovia[tiab] OR Moldovian[tiab] OR Mongolia[tiab] OR Montenegro[tiab] OR Morocco[tiab] OR Ifni [tiab] OR Mozambique[tiab] OR Myanmar[tiab] OR Myanma[tiab] OR Burma[tiab] OR Namibia[tiab] OR Nepal [tiab] OR Netherlands Antilles[tiab] OR New Caledonia[tiab] OR Nicaragua[tiab] OR Niger[tiab] OR Nigeria[tiab] OR Northern Mariana Islands[tiab] OR Oman[tiab] OR Muscat[tiab] OR Pakistan[tiab] OR Palau[tiab] OR Palestine[tiab] OR Panama[tiab] OR Paraguay[tiab] OR Peru[tiab] OR Philippines[tiab] OR Philipines[tiab] OR Phillipines[tiab] OR Phillippines[tiab] OR Poland[tiab] OR Portugal[tiab] OR Puerto Rico[tiab] OR Romania[tiab] OR Rumania[tiab] OR Roumania[tiab] OR Russia[tiab] OR Russian[tiab] OR Rwanda[tiab] OR Ruanda[tiab] OR Saint Kitts[tiab] OR St Kitts[tiab] OR Nevis[tiab] OR Saint Lucia[tiab] OR St Lucia OR Saint Vincent OR St Vincent OR Grenadines OR Samoa OR Samoan Islands OR Navigator Island OR Navigator Islands[tiab] OR Sao Tome[tiab] OR Saudi Arabia[tiab] OR Senegal[tiab] OR Serbia[tiab] OR Montenegro[tiab] OR Seychelles[tiab] OR Sierra Leone[tiab] OR Slovenia[tiab] OR Sri Lanka[tiab] OR Ceylon[tiab] OR Solomon Islands[tiab] OR Somalia[tiab] OR South Africa[tiab] OR Sudan[tiab] OR Suriname[tiab] OR Surinam[tiab] OR Swaziland[tiab] OR Syria[tiab] OR Tajikistan[tiab] OR Tadzhikistan[tiab] OR Tadjikistan[tiab] OR Tadzhik[tiab] OR Tanzania[tiab] OR Thailand[tiab] OR Togo[tiab] OR Togolese Republic[tiab] OR Tonga[tiab] OR Trinidad[tiab] OR Tobago[tiab] OR Tunisia[tiab] OR Turkey[tiab] OR Turkmenistan[tiab] OR Turkmen[tiab] OR Uganda[tiab] OR Ukraine[tiab] OR Uruguay[tiab] OR USSR[tiab] OR Soviet Union[tiab] OR Union of Soviet Socialist Republics[tiab] OR Uzbekistan[tiab] OR Uzbek[tiab] OR Vanuatu[tiab] OR New Hebrides[tiab] OR Venezuela[tiab] OR Vietnam[tiab] OR Viet Nam[tiab] OR West Bank[tiab] OR Yemen[tiab] OR Yugoslavia[tiab] OR Zambia[tiab] OR Zimbabwe[tiab] OR Rhodesia[tiab])))**	
#2	Search **(((((((("Africa"[Mesh]) OR "Central America"[Mesh]) OR "Latin America"[Mesh]) OR "South America"[Mesh]) OR "Asia"[Mesh])) OR "Europe, Eastern"[Mesh])))**	940370

Database: Global Health Library < Week 1 2015>
Search Strategy: 2387

(("Comprehensive Health Insurance" OR "Universal Coverage" OR "Insurance Coverage" OR "Insurance, Hospitalization" OR "Single-Payer System" OR "Prepaid Health Plans" OR "Not-For-Profit Insurance Plans" OR "Insurance, Health") AND ("Developing Countries" OR "Developed Countries" OR "Medically Underserved Area" OR "Africa" OR "Asia" OR "South America" OR "Latin America" OR "Central America" OR "Europe, Eastern")) limited to english
OR

((Comprehensive health insurance* OR universal coverage* OR insurance coverage* OR insurance, hospitalization* OR single-payer system* OR prepaid health plan* OR not-for-profit insurance plan* OR insurance, health) AND (implement* OR facilitat* OR barrier* OR challeng* OR obstacle* OR hurdle* OR limitation* OR enabl* OR resist* OR constraint* OR promot* OR applica* OR integrat* OR utiliz* OR utilis* OR success* OR failure* OR accept* OR lesson* OR experienc* OR scale-up OR roll-out OR sustainab*) AND (developed OR lmic OR lmics OR third world OR lami countr* OR developing OR less* developed OR least developed OR under developed OR underdeveloped OR middle income OR low* income OR underserved OR under-served OR deprived OR poor* OR transition*)) limted to english
