# Supplementary material for: Barriers and facilitators to implementation, uptake and sustainability of community-based health insurance schemes in low- and middle-income countries: a systematic review
Source: Int J Equity Health. 2018 Jan 29;17:13. doi: 10.1186/s12939-018-0721-4 (PMC5789675; doi:10.1186/s12939-018-0721-4)
Supplement: Supplementary file 2 — List of coding themes corresponding to each level of the ecological model (PDF 195 kb) [file 12939_2018_721_MOESM2_ESM.pdf]

Supplementary file 2: List of coding themes corresponding to each level of the ecological model

**Individual level**	**Interpersonal level**	**Community level**	**Financial Arrangement**	**Governance arrangement**	**Delivery Arrangement**
Consumer awareness of existence of scheme/package	Relatives in the scheme	Culture	Amount of premium	Re-imbursement policies	Quality of care
Willingness to enroll	Household size	District-level factors	Pre-payment	Payment arrangement for health care personnel/facilities/centers/clinics	PHC network
Trust in health insurer	Household head characteristics	Community involvement	Method of premium collection	Political economy context (public-private sector)	Availability of health personnel
Socio-demographic factors<br>• Age<br>• Gender,<br>• Nationality,<br>• Economic status<br>• Occupation/employment<br>• Health status<br>• Geographic location/Residency<br>• Education<br>• Household Size<br>• Religion	Social solidarity		Timing of premium collection	Consumer & stakeholder involvement	Management of health personnel

• Ethnicity<br>• Marital status<br>• Migration<br>• Per capita expenditure<br>• Retirement					
Risky health behavior (smoking, alcoholism)			Cost sharing	Policymaker-implementer relationship	Competency of health personnel
Sense of ownership of program			Premium subsidy	Communication between stakeholders	Training of health personnel
Satisfaction of enrollees			Financial support	Stakeholder understanding and involvement	Provider-patient interaction
Being insured with another insurance scheme			Revenue collection	Degree of integrated PHC services	Quality of scheme
Affordability of care			Transport cost	Sustenance of scheme	Facility environment
Consumer understanding of the concept of HBP				Involvement of private sector	Waiting hours
Perceptions or knowledge of scheme management				Ownership of scheme	Commitment of health personnel to package implementation
Closer proximity to nearest health center				Clearly defined roles	Commitment of health care providers

				Government role and support	Referral system
				Management/administrative structure	Working condition
				Common language across all stakeholders	Number of health care network, facilities, centers, clinics
				Legal framework and policy	Distance to health care network, facilities, centers, clinics
				Macro-economic performance	Patient access to specific health facilities, centers, clinics
				Good leadership	Drug supply
				Competition with other insurance	Information system
				Political commitment	Availability of equipment, machinery, etc.
				Policy translation	Incentive mechanisms
				Support for local management team	Equity consideration

				Local health authorities' position on the package	Awareness of health personnel of the package
				Accountability mechanisms	Contracting of service delivery
				Financial management	Geographical coverage of PHC
				Sustainability of funding	Type of facility
				Source of funding	Condition of the facility
				Adverse selection	Place of awareness campaign
				Resource allocation	Availability of protocols
				HBP responds to consumers' needs and preferences	Unit of enrolment
				Content of health benefit package	Adequacy of PHC tests
				Membership criteria	Essential service and catastrophic care
				Marketing and promotion strategies	Prevention services
				Enrolment strategies	
