# Supplementary material for: Barriers and facilitators to implementation, uptake and sustainability of community-based health insurance schemes in low- and middle-income countries: a systematic review
Source: Int J Equity Health. 2018 Jan 29;17:13. doi: 10.1186/s12939-018-0721-4 (PMC5789675; doi:10.1186/s12939-018-0721-4)
Supplement: Supplementary file 3 — List of the excluded studies with reasons for exclusion. (PDF 381 kb) [file 12939_2018_721_MOESM3_ESM.pdf]

Supplementary file 3: List of the excluded studies with reasons for exclusion

Coded reason for exclusion	Reason for exclusion	Number of studies
1	Not in English	**0**
2	Not in low- and middle-income country	**0**
3	Not a primary study or a case study with clear methodology	**2**
4	Proposed scheme	**1**
5	Not about community-based health insurance specifically (e.g. essential benefit package at primary care)	**21**
6	Does not report on any of the outcomes of interest	**5**
7	Focuses on willingness to pay	**2**
8	National health insurance, social health insurance, vouchers or disease-specific packages	**4**
9	Focuses on integration of services	**1**
10	Impact study	**5**

Excluded studies

Author's last name	Year of publication	Coded reason for exclusion	Additional comment
Aggarwal (Aggarwal, 2010)	2010	10	Impact study
Ameli (Tarrier, Taylor, & Gooding, 2008)	2008	5	Not about community-based health insurance specifically (essential package at primary health care)
Arhin (Arhin, 1994)	1994	6	A national health card insurance scheme
Bascolo (Bascolo & Yavich, 2009)	2009	5	Not about community-based health insurance specifically
Baleba (Baleba, Gankpe, Mesenge, Aoun, & Rapp, 2013)	2013	6	Not outcome of interest
Blaakman (Blaakman, Salehi, & Boitard, 2014)	2014	5	Not about community-based health insurance specifically (essential package at primary health care)
Borghi (Borghi et al., 2013)	2013	9	Integration as opposed to implementation
Baza (Baza, 1993)	1993	6	Not outcome of interest
Chen (Chen, Yang, Li, & Li, 2012)	2012	8	insurance coverage, mainly against major<br><br>illnesses treated at inpatient settings
(Dong, Gbangou, De Allegri, Pokhrel, & Sauerborn, 2008)	2008	7	willingness to pay
(De Allegri, Sauerborn,	2009	3	Literature review (not primary study)

Kouyaté, & Flessa, 2009)			
Ensor (Ensor et al., 2002)	2002	5	essential package at primary health care
Govender (Govender et al., 2013)	2013	5	Not about community-based health insurance specifically
Heunis (Heunis, van Rensburg, & Claassens, 2006)	2006	5	essential package at primary health care
Hansen(Gotzsche & Johansen, 2008)	2008	5	essential package at primary health care
Jowett(Jowett, Peters, Reynolds, & Wilson-Barnett, 2001)	2011	5	Not about CBHI specifically
Lin (Lin, Lin, Yen, Loh, & Chwo, 2009)	2009	8	National health insurance
Liu (Liu et al., 2012)	2012	6	Not outcome of interest
Lahariya (Lahariya, Mishra, Nandan, Gautam, & Gupta, 2011)	2011	8	Cash transfer
Molina (Molina & Palazuelos, 2014)	2014	8	Social health insurance
Marnani (Marnani, Teymourzadeh, Bahadori, Ravangard, & Pour)	2012	5	This study focused on health insurance (which could be any type of health insurance) not community-based health insurance specifically
Mugo (Mugo & Wang'ombe, 2009)	2009	5	Not about community-based health insurance (essential package at primary health care)

Mueller (Mueller, Lungu, Acharya, & Palmer, 2011)	2011	5	Not about community-based health insurance (essential package at primary health care)
Nguyen(Nguyen & Knowles, 2010)	2010	5	Not about community-based health insurance
Nandi (Nandi et al., 2012)	2012	5	Not about community-based health insurance
(Onwujekwe, Onoka, et al., 2010)	2010	4	Proposed scheme
(Onwujekwe, Okereke, et al., 2010)	2010	7	Willingness to pay
Petit (Petit, Sondorp, Mayhew, Roura, & Roberts, 2013)	2013	5	Not about community-based health insurance (essential package at primary health care)
Perez (Perez et al., 2013)	2013	5	Not about community-based health insurance (Primary health care interventions)
Rechel (Rechel & Khodjamurodov, 2010)	2010	5	Not about community-based health insurance (essential package at primary health care)
Reddy and Mary	2010	3	Case study with no methods section
Robyn (Robyn et al., 2014)	2012	10	Impact study
Soors (Soors, Devadasan, Durairaj, & Criel, 2010)	2010	5	Not about community-based health insurance (focuses on primary health care)
Tejativaddhana(Tejativaddhana, Briggs, Fraser, Minichiello, & Cruickshank, 2013)	2013	5	Not about community-based health insurance (focuses on primary health care)

Wang (Wang, Gu, & Dupre, 2008)	2008	5	Not about community-based health insurance
Yi(Yi, Zhang, Singer, Rozelle, & Atlas, 2009)	2009	5	Not about community-based health insurance
Yu (Yu et al., 2010)	2010	10	Impact study (health service utilization)
Zhang (Zhang et al., 2010)	2010	5	Not about community-based health insurance
Zhu (Zhu, Dib, Zhang, Tang, & Liu, 2008)	2008	10	Impact study
Zhou (Zhou et al., 2013)	2013	6	Not outcome of interest
Zhou (Zhou et al., 2014)	2014	10	Impact study

References of excluded studies

Aggarwal, A. (2010). Impact evaluation of India's 'Yeshasvini' community-based health insurance programme. *Health Economics, 19 Suppl*, 5-35. doi:http://dx.doi.org/10.1002/hec.1605

Arhin, D. C. (1994). The health card insurance scheme in Burundi: a social asset or a non-viable venture? *Soc Sci Med, 39*(6), 861-870.

Baleba, A. N., Gankpe, F., Mesenge, C., Aoun, O., & Rapp, C. (2013). Cameroonian communal health insurances and managerial performance, a clashing couple. *Tropical Medicine and International Health, 18*, 203. doi:http://dx.doi.org/10.1111/tmi.12163

Bascolo, E., & Yavich, N. (2009). Governance and the effectiveness of the Buenos Aires public health insurance implementation process. *Journal of Ambulatory Care Management, 32*(2), 91-102. doi:http://dx.doi.org/10.1097/JAC.0b013e31819941bb

Baza, A., Hakizimana, A., Hanson, K., Kwizera, F., Van Der Geest, Sjaak (1993). Health insurance and the bamako initiative in Burundi: Value for money?

Blaakman, A. P., Salehi, A. S., & Boitard, R. (2014). A cost and technical efficiency analysis of two alternative models for implementing the basic package of health services in Afghanistan. *Global Public Health, 9*(SUPPL.1), S110-S123. doi:http://dx.doi.org/10.1080/17441692.2013.829862

Borghi, J., Maluka, S., Kuwawenaruwa, A., Makawia, S., Tantau, J., Mtei, G., . . . Macha, J. (2013). Promoting universal financial protection: a case study of new management of community health insurance in Tanzania. *Health Research Policy & Systems, 11*, 21. doi:http://dx.doi.org/10.1186/1478-4505-11-21

Chen, C., Yang, Z., Li, Z., & Li, L. (2012). Accuracy of several cervical screening strategies for early detection of cervical cancer: A meta-analysis. *International Journal of Gynecological Cancer, 22*(6), 908-921.

De Allegri, M., Sauerborn, R., Kouyaté, B., & Flessa, S. (2009). Community health insurance in sub-Saharan Africa: what operational difficulties hamper its successful development? *Tropical Medicine & International Health, 14*(5), 586-596.

Dong, H., Gbangou, A., De Allegri, M., Pokhrel, S., & Sauerborn, R. (2008). The differences in characteristics between health-care users and non-users: implication for introducing community-based health insurance in Burkina Faso. *The European Journal of Health Economics, 9*(1), 41-50.

Ensor, T., Dave-Sen, P., Ali, L., Hossain, A., Begum, S. A., & Moral, H. (2002). Do essential service packages benefit the poor? Preliminary evidence from Bangladesh. *Health Policy Plan, 17*(3), 247-256.

Gotzsche, P. C., & Johansen, H. K. (2008). House dust mite control measures for asthma: Systematic review. *Allergy: European Journal of Allergy and Clinical Immunology, 63*(6), 646-659.

Govender, V., Chersich, M. F., Harris, B., Alaba, O., Ataguba, J. E., Nxumalo, N., & Goudge, J. (2013). Moving towards universal coverage in South Africa? Lessons from a voluntary government insurance scheme. *Glob Health Action, 6*, 19253. doi:http://dx.doi.org/10.3402/gha.v6i0.19253

Heunis, J. C., van Rensburg, H. C., & Claassens, D. L. (2006). Assessment of the implementation of the primary health care package at selected sites in South Africa. *Curationis, 29*(4), 37-46.

Jowett, S., Peters, M., Reynolds, H., & Wilson-Barnett, J. (2001). The UKCC's Scope of Professional Practice--some implications for health care delivery. *J Nurs Manag, 9*(2), 93-100.

Lahariya, C., Mishra, A., Nandan, D., Gautam, P., & Gupta, S. (2011). Additional cash incentive within a conditional cash transfer scheme: a 'controlled before and during' design evaluation study from India. *Indian J Public Health, 55*(2), 115-120. doi:10.4103/0019-557x.85245

Lin, J. D., Lin, Y. W., Yen, C. F., Loh, C. H., & Chwo, M. J. (2009). Received, understanding and satisfaction of National Health Insurance premium subsidy scheme by families of children with disabilities: a census study in Taipei City. *Research in Developmental Disabilities, 30*(2), 275-283. doi:http://dx.doi.org/10.1016/j.ridd.2008.04.004

Liu, X., Tang, S., Yu, B., Phuong, N. K., Yan, F., Thien, D. D., & Tolhurst, R. (2012). Can rural health insurance improve equity in health care utilization? A comparison between China and Vietnam.

International Journal for Equity in Health, 11, 10. doi:http://dx.doi.org/10.1186/1475-9276-11-10
Marnani, A. B., Teymourzadeh, E., Bahadori, M., Ravangard, R., & Pour, J. S. Challenges of a Large Health Insurance Organization in Iran: A Qualitative Study.
Molina, R. L., & Palazuelos, D. (2014). Navigating and circumventing a fragmented health system: the patient's pathway in the Sierra Madre Region of Chiapas, Mexico. *Medical Anthropology Quarterly, 28*(1), 23-43. doi:http://dx.doi.org/10.1111/maq.12071
Mueller, D. H., Lungu, D., Acharya, A., & Palmer, N. (2011). Constraints to implementing the Essential Health Package in Malawi. *PLoS ONE, 6*(6), e20741. doi:10.1371/journal.pone.0020741
Mugo, M. G., & Wang'ombe, J. K. (2009). Costing resource gaps for the delivery of minimum essential health services package in the Coast Province, Kenya. *East Afr J Public Health, 6*(3), 326-331.
Nandi, S., Kanungo, K., Khan, M. H., Soibam, H., Mishra, T., & Garg, S. (2012). A study of Rashtriya Swasthya Bima Yojana in Chhattisgarh, India. *BMC Proceedings, 6*.
Nguyen, H., & Knowles, J. (2010). Demand for voluntary health insurance in developing countries: the case of Vietnam's school-age children and adolescent student health insurance program. *Soc Sci Med, 71*(12), 2074-2082. doi:10.1016/j.socscimed.2010.09.033
Onwujekwe, O., Okereke, E., Onoka, C., Uzochukwu, B., Kirigia, J., & Petu, A. (2010). Willingness to pay for community-based health insurance in Nigeria: do economic status and place of residence matter? *Health Policy and Planning, 25*(2), 155-161.
Onwujekwe, O., Onoka, C., Uguru, N., Nnenna, T., Uzochukwu, B., Eze, S., . . . Petu, A. (2010). Preferences for benefit packages for community-based health insurance: an exploratory study in Nigeria. *BMC health services research, 10*(1), 162.
Perez, L. G., Sheridan, J. D., Nicholls, A. Y., Mues, K. E., Saleme, P. S., Resende, J. C., . . . Leon, J. S. (2013). Professional and community satisfaction with the Brazilian family health strategy. *Revista de Saude Publica, 47*(2), 403-413. doi:http://dx.doi.org/10.1590/S0034-8910.2013047003868
Petit, D., Sondorp, E., Mayhew, S., Roura, M., & Roberts, B. (2013). Implementing a Basic Package of Health Services in post-conflict Liberia: perceptions of key stakeholders. *Social Science & Medicine, 78*, 42-49. doi:http://dx.doi.org/10.1016/j.socscimed.2012.11.026
Rechel, B., & Khodjamurodov, G. (2010). International involvement and national health governance: the basic benefit package in Tajikistan. *Social Science & Medicine, 70*(12), 1928-1932. doi:http://dx.doi.org/10.1016/j.socscimed.2010.02.029
Robyn, P. J., Barnighausen, T., Souares, A., Traore, A., Bicaba, B., Sie, A., & Sauerborn, R. (2014). Provider payment methods and health worker motivation in community-based health insurance: A mixed-methods study. *Social Science and Medicine, 108*, 223-236. doi:http://dx.doi.org/10.1016/j.socscimed.2014.01.034
Soors, W., Devadasan, N., Durairaj, V., & Criel, B. (2010). Community health insurance and universal coverage: multiple paths, many rivers to cross. *World health report*.
Tarrier, N., Taylor, K., & Gooding, P. (2008). Cognitive-behavioral interventions to reduce suicide behavior: A systematic review and meta-analysis. *Behavior Modification, 32*(1), 77-108.
Tejativaddhana, P., Briggs, D., Fraser, J., Minichiello, V., & Cruickshank, M. (2013). Identifying challenges and barriers in the delivery of primary healthcare at the district level: a study in one Thai province. *International Journal of Health Planning & Management, 28*(1), 16-34. doi:http://dx.doi.org/10.1002/hpm.2118
Wang, H., Gu, D., & Dupre, M. E. (2008). Factors associated with enrollment, satisfaction, and sustainability of the New Cooperative Medical Scheme program in six study areas in rural Beijing. *Health Policy, 85*(1), 32-44. doi:10.1016/j.healthpol.2007.06.003
Yi, H., Zhang, L., Singer, K., Rozelle, S., & Atlas, S. (2009). Health insurance and catastrophic illness: a report on the New Cooperative Medical System in rural China. *Health Economics, 18 Suppl 2*, S119-127. doi:http://dx.doi.org/10.1002/hec.1510
Yu, B., Meng, Q., Collins, C., Tolhurst, R., Tang, S., Yan, F., . . . Liu, X. (2010). How does the New Cooperative Medical Scheme influence health service utilization? A study in two provinces in

rural China. *BMC Health Services Research*, *10*, 116. doi:http://dx.doi.org/10.1186/1472-6963-10-116

Zhang, L., Cheng, X., Liu, X., Zhu, K., Tang, S., Bogg, L., . . . Tolhurst, R. (2010). Balancing the funds in the New Cooperative Medical Scheme in rural China: determinants and influencing factors in two provinces. *International Journal of Health Planning & Management*, *25*(2), 96-118. doi:http://dx.doi.org/10.1002/hpm.988

Zhou, Z., Su, Y., Gao, J., Campbell, B., Zhu, Z., Xu, L., & Zhang, Y. (2013). Assessing equity of healthcare utilization in rural China: results from nationally representative surveys from 1993 to 2008. *International Journal for Equity in Health*, *12*, 34. doi:http://dx.doi.org/10.1186/1475-9276-12-34

Zhou, Z., Zhu, L., Zhou, Z., Li, Z., Gao, J., & Chen, G. (2014). The effects of China's urban basic medical insurance schemes on the equity of health service utilisation: evidence from Shaanxi Province. *International Journal for Equity in Health*, *13*, 23. doi:http://dx.doi.org/10.1186/1475-9276-13-23

Zhu, M., Dib, H. H., Zhang, X., Tang, S., & Liu, L. (2008). The influence of health insurance towards accessing essential medicines: the experience from Shenzhen labor health insurance. *Health Policy*, *88*(2-3), 371-380. doi:http://dx.doi.org/10.1016/j.healthpol.2008.04.008
