# Supplementary material for: Barriers and facilitators to implementation, uptake and sustainability of community-based health insurance schemes in low- and middle-income countries: a systematic review
Source: Int J Equity Health. 2018 Jan 29;17:13. doi: 10.1186/s12939-018-0721-4 (PMC5789675; doi:10.1186/s12939-018-0721-4)
Supplement: Supplementary file 4 — Overview of the characteristics of each included study (PDF 1046 kb) [file 12939_2018_721_MOESM4_ESM.pdf]

Author last name, Funding, Study Design	Objective	Methods	Population	Results: Description of package or scheme	Socio-demographic factors	Results: Barriers	Results: Facilitators
**Author & year**<br>Uzochukwu et al. 2009<br><br>**Funding**<br>DFID<br><br>**Study design**.<br>Mixed methods	This study explores the CBHI policy development and implementation process and the factors that have constrained or enhanced its implementations.	**Sampling size and method**<br>*In depth interview*<br>N= 1 senior politician, 8 state policy makers involved in the scheme, and 5 LGA officials. Also with 4 health workers per site in the focal health facilities. 2 members of the Community Health Management Organization (Managers of the scheme) were also interviewed in each site<br>*Focus group discussion*<br>N= In total, 8 FGDs were conducted in each catchment area, with 9-10 members in each FGD.<br>Purposive sampling<br>*Interviews*<br>All the 16 members of the community health committees in the 2 sites<br>*Household survey*<br>N= 1000 respondents (500 from each community)<br>simple random sampling	**Sample population**<br>Household, members and non-members of the CBHI, policymakers, health workers, managers of the scheme<br><br>**Setting**<br>Anambra State, Nigeria	**Type:** community based health insurance<br>**Content:** medical treatment is restricted to those obtainable at primary healthcare facilities<br>**population covered:** members and non-members<br>**Enrolment rate:** not reported (only rates in the case study were mentioned)<br>**Unit of Enrollment:** individual and households<br>**Source of fund:** beneficiaries, government, capitation fund<br>**Premium:** yes<br>**Cost-sharing:** flat rate<br>**Role of government:** financial, governance<br>**Provider-payment method:** capitation fund	There were no significant SES difference in registration, and willingness to renew registration, for the respondent as well as for other household members. In addition, the number of registered respondents indicating increase in facility utilization did not differ significantly across SES groups.	**Community involvement in decision making**<br>Community participation was very poor in community B. This was as a result of lack of proper mobilization of the community by the managers and health workers… some people who could have registered with the scheme did not do so because of lack of information.<br><br>**Management/administrative structure**<br>The attitude of the Coordinator disenchanted many members of the CHC, and the community at large, and may have contributed to the poor performance of the scheme in this community<br><br>**Attitude factors**<br>*Trust*<br>More people from this community did not register because of a lack of trust in those	**Community involvement in decision making**<br>-Whilst the town union and another member assisted in the renovation of the infrastructure, some individuals paid the premium for other members of the community, where about 77.9% (155/199) of those registered showed the willingness to register for other members.<br>-In addition, the Igwe played a prominent role in the management of the scheme… these actions seemed to have contributed to the success of the scheme in community A.<br><br>**Facility-related factors**<br>Availability of good quality treatment was the next most common reason for registering<br><br>**Attitude factors**<br>*Trust*<br>The majority of the participants trust the

		**Time frame**<br>December 2006 to February 2007<br><br>**Data collection**<br>Document review<br>In depth interviews<br>Focus group discussions<br>Household surveys (interviewer-administered questionnaire)<br><br>**Data analysis**<br>-Principal Components Analysis<br>-Stake Holder Analysis<br>-Forcefield analysis				managing the CBHI funds<br><br>**Political economy context**<br>The Governor was removed in March 2006 as a result of political tensions in the state, and the Commissioner left with him. Following his removal, state interest in and support for CBHI dwindled, and there has been no subsequent expansion of the scheme.<br><br>**Government support**<br>Legal backing of the CBHI policy would, at least, have given the policy leverage as to be continued by the next administration with the government being constitutionally committed to continuing it.<br>- It is also quite obvious that the implementation of CBHI policy was constrained by policy makers‟ seemingly weak understanding of how policy objectives and design could provoke opposition at the local	CBHI managers and the community members to manage CBHI

						level and, hence, derail implementation.<br><br>**Human resource planning and management**<br>Absence of doctors in the health centers constrained implementations	
**Author & year**<br>Panda et al, 2013<br><br>**Funding**<br>European Union's FP7 programme, project–Community-Based Health Insurance in India, HEALTH-F2-2009-223518<br><br>**Study design**<br>RCT	The purpose of this study is to examine and identify factors that drive enrolment in CBHI schemes launched in three rural locations (one in Pratapgarh, Uttar Pradesh, one in Kanpur Dehat, Uttar Pradesh and the third in Vaishali, Bihar) in India in 2010–11.	**Sampling size and method**<br>N= 1335 households or 7722 individuals<br>Cluster sampling<br><br>**Time frame**<br>March 2010<br><br>**Data collection**<br>-Baseline survey<br>- information on actual enrolments, premium payments and claims, maintained by MIA's management information system<br><br>**Data analysis**<br>Logit regression	**Sample population**<br>SHG-affiliated households<br><br>**Setting**<br>Rural Bihar and Uttar Pradesh, India	**Type:** community based health insurance (CBHI)<br>**Content:**<br>*Sanjivani:* hospitalization, maternity care<br>*Jeevan Sanjivani:* hospitalization, outpatient care<br>*Swastha Kamal:* outpatient care<br>**Population covered:** entire household<br>**Enrolment rate:**<br>*Sanjivani*: 40% (household), 23% (individual)<br>*Jeevan Sanjivani*: 29% (household), 15% (individual)<br>*Swastha Kamal*: 46% (household), 30% (individual)<br>**Unit of Enrollment:** household<br>**Source of fund:** premiums, and in *Sanjivani* some OOP	**Socio-demographic factors**<br><br>*Household size*<br>Belonging to a joint family or a larger household has no bearing on CBHI uptake.<br><br>*Economic status*<br>Belonging to an economically and socially disadvantaged community increased affiliation to the CBHI<br><br>*Education*<br>While there is some evidence that household heads with some primary education, as compared with those who are illiterate, are more likely to join a CBHI scheme, the effect is restricted to Vaishali.	**Household dynamics**<br>Intra-household pooling of income, which is an indicator of risk-pooling and can help smooth consumption in the event of a shock, exerts a negative and large effect on enrolment<br><br>Household financial liabilities may indicate household ability to access credit, which may have a negative effect on CBHI enrolment	**Health status**<br>Households with children seem to be more risk averse and/or expect a higher need for health care and subsequently more likely to want to join insurance

				**Premium:** yes<br>**Cost-sharing:** not reported<br>**Role of government:** not reported<br>**Provider-payment method:** not reported			
**Author & year**<br>Lammers et al, 2010<br><br>**Funding**<br>Not clearly reported<br><br>**Study design**.<br>Quantitative	This study assesses the extent of adverse selection together with an analysis of the determinants of the demand for voluntary health insurance.	**Sampling size and method**<br>N= 677 households, 2338 individuals (complete data on 1941)<br>Random selection<br><br>**Time frame**<br>2008<br><br>**Data collection**<br>Household survey<br><br>**Data analysis**<br>-logistic regression	**Sample population**<br>Small entrepreneur<br><br>**Setting**<br>Lagos, Nigeria	**Type:** voluntary micro health insurance scheme<br>**Content:** The insurance scheme covers, among other things, primary and outpatient care, consultation with specialists and HIV/AIDS treatment, care and support.<br>**population covered:** Informal sector workers<br>**Enrolment rate:** the actual enrolment rate among the full population cannot be calculated due to control problems with respect to scheme eligibility. (Referred to in the paper by 6%)<br>**Unit of Enrollment:** household<br>**Source of fund:** government or donors, beneficiaries<br>**Premium:** yes (90% subsidized by donors)<br>**Cost-sharing:** co-payment (13.5$)<br>**Role of government:**	**Socio-demographic factors**<br><br>*Religion*<br>Being Christian reduced the propensity to insure<br><br>.<br>*Economic status*<br>The propensity to be enrolled is seven times higher for persons from the highest quintile<br><br>**Personal pre-disposition**<br><br>*Health status*<br><br>Individuals who were actually in need of healthcare had a larger propensity to be insured		**Household dynamics**<br><br>*Household size*<br><br>Persons from larger families are more likely insured

				Financial<br>**Provider-payment method:** subsidies			
**Author & year**<br>Ito et al, 2010<br><br>**Funding**<br>Not reported<br><br>**Study design**.<br>Quantitative	In the present paper, we try to understand the mechanism behind low-income households' insurance take-up decisions based on recent empirical insurance literature and on behavioural literature.	**Sampling size and method**<br>N= 209 household<br>Random selection<br><br>**Time frame**<br>September 2008<br><br>**Data collection**<br>Questionnaire<br><br>**Data analysis**<br>-descriptive analysis<br>-Univariate regression	**Sample population**<br>Households<br><br>**Setting**<br>Rural Bangalore, Karnataka, India	**Type:** dairy cooperative-based insurance scheme<br>**Content:** It offers a low priced product covering over 1,600 defined surgical procedures to farmers and their family members. The beneficiaries can receive cashless treatment at a network of over 135 hospitals, both public and private, across Karnataka. Normal delivery is covered. Children born prematurely or with low birth weight who require special care during the first seven days after birth are covered. In addition, the policyholders can receive free outpatient consultation at all participating hospitals, discounted tariffs for investigations and inpatient treatment for non-covered hospitalization.	*Health status/ economic status*<br>Interestingly, we also find that households with sick household heads are *less* likely to purchase insurance. This might capture the fact that households with sick households have lower incomes and have difficulty in financing the insurance premium.<br>- Households with healthy head members are more likely to purchase the policies.<br><br>*Economic status*<br>The negative coefficient implies that credit constrained households are cash constrained in buying insurance, and/or that near-constrained households are forward looking enough to buy insurance.		**Health status**<br>Households with a higher ratio of sick members are more likely to purchase Insurance<br><br>**Household dynamics**<br>Households owning barns are also more likely to purchase policies, a fact that simply depicts the reality that Yeshasvini is a dairy cooperative-based insurance scheme.

				**Population covered:** Dairy cooperative farmers and poor people across the state of Karnataka. (Yeshasvini is open to all cooperative society members who have been in the cooperative society for at least six months. Ages of the insured range from 0 to 75 years.) **Enrolment rate:** not reported (low) **Unit of Enrollment:** household **Source of fund:** self-funded **Premium:** yes, Rs 120 (Approximately US$2.4) per year for an adult or a child. For families of five or more members, the premium is discounted by 15%. **Cost-sharing:** not reported **Role of government:** Governance **Provider-payment method:** not reported			

**Author & year**<br>Bhat et al, 2006<br><br>**Funding**<br>Insurance research center, IIM Ahmedabad<br><br>**Study design**.<br>Quantitative	The objective of this paper is to analyse factors determining the demand for private health insurance in a micro insurance scheme setting.	**Sampling size and method**<br>N= 301 household<br>Stratified random sampling<br><br>**Time frame**<br>Not reported<br><br>**Data collection**<br>Survey<br><br>**Data analysis**<br>econometric analysis<br>regression analysis<br>descriptive analysis	**Sample population**<br>Household<br><br>**Setting**<br>India	**Type:** micro insurance scheme<br>**Content:** hospitalization costs (up to the coverage amount), OPD (free), some medicines and diagnostic tests are excluded (some discounts on these services do exist), maternity coverage<br>**population covered:** lower and middle income groups<br>**Enrolment rate:** not reported<br>**Unit of Enrollment:** household<br>**Source of fund:** beneficiaries<br>**Premium:** yes, 90-2325<br>**Cost-sharing:** not reported<br>**Role of government:** not reported<br>**Provider-payment method:** not reported	*Household size*<br>On average we can see that insured households have bigger family size than non-insured households ones. Similarly, insured households have more children than non-insured households.<br><br>*Economic status*<br>The higher the income of the household, higher is the probability of buying health insurance of the household.<br><br>*Age*<br>In higher age groups people have more probability of purchasing health insurance while in lower age groups, age is not statistically significant.		**Consumer awareness of scheme**<br>Knowledge about insurance is very important and one of the important reasons for buying health insurance and in the results this factor came significant and positive which indicates that building more awareness about health insurance will influence the probability of buying health insurance<br><br>**Membership criteria**<br>Coverage of illnesses, which indicates that if the policy is better designed in terms of the illnesses which are covered, there is higher chance of people buying it
**Item**	**Objective**	**Methods**	**Population**	**Results: Description of package or scheme**	**Results: Socio-demographic factors**	**Results: Barriers**	**Results: Facilitators**
**Author & year**<br>Mladovsky et al, 2014<br><br>**Funding**<br>Stewart Halley Trust<br><br>**Study design**<br>Mixed methods multiple	This exploratory study proposes that an under-researched determinant of<br>CBHI enrolment is social capital.	**Sampling size and method**<br>*Qualitative*:<br>N= 109 individual; purposeful sampling<br><br>*Quantitative*:<br>N=720 individual;	**Sample population**<br>Household<br><br>**Setting**<br>Senegal	**Type:** Community-based health insurance<br>**Content:** Health posts, health hut, maternal and child health centre<br>**Population covered:** Informal sector<br>**Enrolment rate:** 4% or less	**Education**<br>Members were likely to be better educated, but the results were not statistically significant<br><br>**Per capita expenditure**<br>In Soppante and WAW, CBHI households had	**Household dynamics**<br>*Household size*<br>Several non-members said they had not enrolled in CBHI because they could not afford to pay the	**Personal pre-disposition**<br>Households enrolled in CBHI were significantly more likely to be members of other associations compared to non-CBHI households,

**Author & year**<br>Bending et al, 2011<br><br>**Funding**<br>The British Academy<br><br>**Study design**.<br>Quantitative	"We examine household's micro insurance participation, i.e. the usage of micro insurance, whereas the use of insurance, i.e. the actual provision, is determined by the demand and the supply of insurance. Thereby, we emphasize primarily life and health insurance and focus on voluntary insurance offers."	**Sampling size and method**<br>N= 330 household<br><br>Random selection.<br><br>**Time frame**<br>2008<br><br>**Data collection**<br>Questionnaire<br><br>**Data analysis**<br>-descriptive analysis<br>-econometric analysis<br>-regression	**Sample population**<br>Household<br><br>**Setting**<br>Sri Lanka	**Type:** health micro insurance<br>**Content:** there exists several micro-insurance products including death benefits provided for instance by the five MFIs surveyed, which can be interpreted as a term life insurance, or providing in addition to death, accident, hospitalization, health and other benefits.<br>**population covered:**<br>**Enrolment rate:** not reported<br>**Unit of Enrollment:**<br>**Source of fund:** not reported<br>**Premium:** yes<br><br>**Cost-sharing:** not reported<br>**Role of government:** Not reported<br>**Provider-payment method:** not reported	*Gender*<br>The results provide evidence that female-headed households are significantly more likely to be member in a MFI than male-headed households in Sri Lanka.<br><br>*Age*<br>Insurance buyers are significantly older than insurance non-buyers.<br><br>*Household size/marital status*<br>Insurance buyers live in larger households and among them are slightly more married household heads than among non-buyers<br>*Health status*<br>The ratio of ill household members is significantly higher among participants than non-participants.<br><br>*Economic status*<br>Households from the richest quintile are more likely to be<br>enrolled in a MFI compared to those from the poorest quintiles<br><br>*Education level*	**Household dynamics**<br>*Household size*<br>Several non-members said they had not enrolled in CBHI because they could not afford to pay the premium for their extended kin.<br><br>**Marketing and Promotion Strategies**<br>Diversified access to information was a determinant of enrolment, as all types of interviewees complained that information about the CBHI schemes was scarce.<br><br>**Health status**<br>In Soppante, members reported worse health for every indicator, possibly indicating adverse selection, although this was not statistically significant	**Management/Administrative Structure**<br><br>Accountability and being informed of mechanisms of controlling abuse/fraud are all correlated with remaining in the scheme.<br><br>**Personal pre-disposition**<br>Households enrolled in CBHI were significantly more likely to be members of other associations compared to non-CBHI households, controlling for age and gender only<br><br>**Social solidarity**<br>Members of CBHI were more likely than non-members to have bonding social capital, as measured by having privileged social relationships.<br>Believing that the community would cooperate in an emergency was significantly positively correlated with enrolment

					household heads with no formal, primary or secondary education are less likely to be enrolled in a MFI or participate in the microfinance market than higher educated heads		**Community involvement**<br>CBHI members were more than two times as likely to report having control over decisions made in the community or by their neighbours which affected their daily life compared to non-members<br><br>**Political economy context**<br>More than 60% of respondents reported voting in the last local elections. Qualitative results suggest that members believed CBHI schemes were managed in a democratic manner, perhaps helping to explain why voting was correlated with enrolment

Item	Objective	Methods	Population	Results: Description of package or scheme	Results: Socio-demographic factors	Results: Barriers	Results: Facilitators
case study design which included a household survey and semi-structured interviews		Disproportionate stratified sampling method<br>**Time frame**<br>March-August 2009<br>**Data collection**<br>-Questionnaire<br>-Semi structured interviews<br>**Data analysis**<br>-Descriptive and regression analysis<br>-Content analysis		**Unit of Enrollment:** household<br>**Source of fund:** beneficiaries<br>**Premium:** yes<br>**Cost-sharing:** not reported<br>**Role of government:** governance<br>**Provider-payment method:** not reported	significantly higher levels of expenditure than non-member households<br><br>**Economic status**<br>In Ndondol, CBHI member households were wealthier	premium for their extended kin.<br><br>**Marketing and Promotion Strategies**<br>Diversified access to information was a determinant of enrolment, as all types of interviewees complained that information about the CBHI schemes was scarce.<br><br>**Health status**<br>In Soppante, members reported worse health for every indicator, possibly indicating adverse selection, although this was not statistically significant	controlling for age and gender only<br><br>**Social solidarity**<br>Members of CBHI were more likely than non-members to have bonding social capital, as measured by having privileged social relationships.<br>Believing that the community would cooperate in an emergency was significantly positively correlated with enrolment<br><br>**Community involvement**<br>CBHI members were more than two times as likely to report having control over decisions made in the community or by their neighbours which affected their daily life compared to non-members<br><br>**Political economy context**<br>More than 60% of respondents reported

Item	Objective	Methods	Population	Results: Description of package or scheme	Results: Socio-demographic factors	Results: Barriers	Results: Facilitators
							voting in the last local elections. Qualitative results suggest that members believed CBHI schemes were managed in a democratic manner, perhaps helping to explain why voting was correlated with enrolment
**Author & year**<br>Mulupi et al, 2013<br><br>**Funding**<br>Not reported<br><br>**Study design**<br>Qualitative and quantitative	The study aimed to explore communities' understanding and perceptions of health insurance and their preferred designs for a NHIS.	**Sampling size and method**<br>*Quantitative*:<br>N=594 households, 2419 individuals<br>Random sampling<br>Cluster sampling<br><br>*Qualitative*:<br>N=16 focus group sessions based on quantitative sampling methods.<br><br>**Time frame**<br>Not reported<br><br>**Data collection**<br>Data were collected from 6 villages (3 from each district).<br>-Cross sectional household survey<br>-Focus groups	**Sample population**<br>Household<br><br>**Setting**<br>Nyeri and Kirinyaga districts, Central Kenya	**Type:** Community-based Health Insurance Schemes<br>**Content:** Inpatient care in selected public and faith-based health facilities<br>**population covered:** open to all, target the poor<br>**Enrolment rate:** 1.2% of Kenyan population<br>**Unit of Enrollment:** individual<br>**Source of fund:** beneficiaries' Contributions<br>**Premium:** yes<br>**Cost-sharing:** copayments<br>**Role of government:** not reported<br>**Provider-payment method:** not reported	**Economic status**<br>People of high socioeconomic status were more likely to join health insurance schemes compared to the rest of the population	**Health status**<br>Some did not see the reason of making contributions towards health insurance when they were in good health<br><br>**Consumer understanding of concept of health insurance**<br>Limited understanding of health insurance prevented people from becoming members. It was not always clear how health insurance schemes function<br><br>**Marketing and Promotion Strategies**<br>Participants expressed lack of awareness of health insurance and	**Health status**<br>For some, ill health was a motivation for belonging to a scheme<br><br>**Amount and timing of premium**<br>One of the Factors that made it easy for people to belong to health insurance schemes was affordable contribution rates. Another factor was favourable contribution mechanisms, where members were allowed to make their contributions in instalments or having them linked to agricultural produce<br><br>**Financial protection**<br>Health insurance schemes were perceived

Item	Objective	Methods	Population	Results: Description of package or scheme	Results: Socio-demographic factors	Results: Barriers	Results: Facilitators
		**Data analysis**<br>-Descriptive analysis<br>-Thematic analysis				attributed this to limited efforts to promote CBHIs.<br><br>**Facility related factors**<br>*Waiting hour*<br>Perceived poor service provision, exemplified by long waiting times, hindered people from joining health insurance schemes and/or contributed to drop out rates.<br><br>*Facility environment*<br>Poor service provision, exemplified by corruption and conflict of interest, hindered people from joining health insurance schemes and/or contributed to drop out rates.<br><br>*Supplies and materials*<br>Poor service provision, exemplified by lack of laboratory equipment and x-ray machines, inadequate ward facilities and poor diet, hindered people from joining health insurance schemes and/or	to have many advantages by both members and non-members, including offering financial protection to members<br><br>**Social solidarity**<br>A Perceived advantage of scheme was making members feel at ease when their relatives were in hospitals and building on solidarity to help other community members.

Item	Objective	Methods	Population	Results: Description of package or scheme	Results: Socio-demographic factors	Results: Barriers	Results: Facilitators
						contributed to drop out rates.<br><br>*Interpersonal skills*<br>Poor service provision, exemplified by discrimination of patients according to scheme membership or perceived socioeconomic status, hindered people from joining health insurance schemes and/or contributed to drop out rates.<br>Other complaints included poor hospitality, including rude hospital staff<br><br>**Package content**<br>Inadequate benefit packages hindered people from joining health insurance schemes and/or contributed to drop out rates<br><br>**Cost-sharing**<br>High co-payments hindered people from joining health insurance schemes and/or contributed to drop out rates.	

Item	Objective	Methods	Population	Results: Description of package or scheme	Results: Socio-demographic factors	Results: Barriers	Results: Facilitators
						**Amount and timing of premium**<br>While members reported that contribution rates were well linked to peak agricultural seasons, allowing many people to meet the deadlines, non-members reported that this was not the case for most CBHIs.<br><br>Not being able to pay in instalments was another reason given that made it difficult for people to join CBHIs	
**Author & year**<br>Ranson et al, 2006<br><br>**Funding**<br>Wellcome Trust (UK).<br><br>**Study design**<br><br>Quantitative and qualitative	This paper describes and analyses the experience of piloting a preferred provider system (PPS) for rural members of Vimo SEWA, a fixed-indemnity, community-based health insurance (CBHI) scheme run by the Self-Employed Women's Association (SEWA)	**Sampling size and method**<br>N= not reported<br>Of 23 subdistricts the 16 with the highest number of women, Vimo SEWA members were selected for sampling. Eight of these 16 were randomly selected for implementation of PPS.<br>*Qualitative*:<br>Purposive sampling<br><br>**Time frame**<br>-Household survey:	**Sample population**<br>Household, aagewans women<br><br>**Setting**<br>Rural Gujarat, India	**Type:** Voluntary trade union<br>**Content:** life, hospitalization and asset insurance as an integrated package.<br>**population covered:** poor, self-employed women workers aged between 18 and 55<br>**Enrolment rate:** not reported<br>**Unit of Enrollment:** household<br>**Source of fund:** beneficiaries<br>**Premium:** most members pay an annual		**Accessibility to facility**<br>*Geographical Coverage*<br>selected hospitals in some sub districts were too far away for members to access easily<br><br>**Consumer awareness of scheme**<br>Lack of awareness about the preferred provider system among members and lack of knowledge about the identity or location of PPS hospitals.<br><br>**Attitude factors**	

Item	Objective	Methods	Population	Results: Description of package or scheme	Results: Socio-demographic factors	Results: Barriers	Results: Facilitators
		1 April-31 December 2006).<br>-Questionnaire:<br>January and March 2006<br>-Interview:<br>December 2004 and between June and September 2005<br>-In depth *interviews*:<br>November 2005 and January 2006<br>-*FGD*:<br>December 2004<br><br>**Data collection**<br>FGD, in depth interviews, interviews, questionnaire, household survey<br><br>**Data analysis**<br>-Thematic analysis<br>-Descriptive analysis		premium. These 'annual members' remain eligible for hospitalization benefits until 70 years of age, provided that they remain insured every year after the age of 55.<br>**Cost-sharing:** OOP<br>**Role of government:** not reported<br>Governance<br>**Provider-payment method:**<br>Not reported- referred provider system (PPS) for rural members		*Perception of scheme*<br>The members' poor perception of a PPS facility, or lack of familiarity with it, were also reasons for members not using the PPS.<br><br>**Cost-sharing**<br>Even under the preferred provider system, user fees continue to pose a financial barrier to the insured—they have to mobilize funds to cover the costs of medicines, supplies, registration fee, etc. before receipt of cash payment from Vimo SEWA.<br>Although the first payment of benefits from Vimo SEWA may be made as early as 24-48 hours after admission, this delay may be enough to deter poor members from seeking hospitalization.<br><br>**Management/administrative structure**<br>A variety of administrative problems.<br>**Marketing and promotion strategies**	

Item	Objective	Methods	Population	Results: Description of package or scheme	Results: Socio-demographic factors	Results: Barriers	Results: Facilitators
						Lack of information on availability and location of services<br>**Accessibility of facility**<br>*Geographical coverage*<br>Physical barriers (distances, difficult terrain), lack of transportation	

Item	Objective	Methods	Population	Results: Description of package or scheme	Results: Socio-demographic factors	Results: Barriers	Results: Facilitators
**Author & year**<br>Kamau et al. 2014<br><br>**Funding**<br>Not reported<br><br>**Study design**<br>Quantitative (cross-sectional)	To assess Samburu CBHIS and give recommendations on how to rejuvenate the scheme.	**Sampling size and method**<br>286, random; systematic random sampling<br><br>**Time frame**<br>November 2005<br><br>**Data collection**<br>Structured questionnaire<br><br>**Data analysis**<br>Descriptive statistics	**Sample population**<br>Household<br><br>**Setting**<br>Samburu district in northern Kenya	**Type:** Community-based health care insurance schemes<br>**Content:**<br>Curative services include treatment of common endemic diseases while preventive services are mainly mother/child clinic services and health education and promotion.<br>Laboratory and in-patient services are available in one of the dispensaries.<br>In addition to the mentioned services, CBHIS members are		**Household dynamic**<br><br>The scheme had very few members from female- headed households as well as youth. Female-headed households and youth are more likely to be on the lower-income percentile in the study society due to its paternalistic orientation<br><br>**Role of culture**<br>The majority of residents in this area believe and practice herbal medicine; conventional medicine is	

Item	Objective	Methods	Population	Results: Description of package or scheme	Results: Socio-demographic factors	Results: Barriers	Results: Facilitators
				entitled to emergency ambulance and referral services.<br>**Population covered**: CBHIS members and non-members<br>**Enrolment rate:** 20%<br>**Unit of Enrollment:** household<br>**Source of fund:** NGO's subsidy, beneficiaries<br>**Premium:** USD 37.5<br>**Cost-sharing:** NR<br>**Role of government:** None<br>**Provider-payment method**<br>NR		seen as a last resort. The situation is made worse by the fact that money is not always the way of transacting herbal medicine since barter trade is still rampant in this society<br><br>**Membership criteria**<br>*Equity consideration*<br>16% of respondents indicated that there were some members of their family for whom it was more difficult to access health care than others especially the elderly and the expectant mothers, while children were perceived to easily get health care since they could be carried to a health facility, if necessary.<br><br>**Attitude Factors**<br>*Sense of ownership*<br>Only 10% had a sense of ownership of scheme<br><br>*Trust*	

Item	Objective	Methods	Population	Results: Description of package or scheme	Results: Socio-demographic factors	Results: Barriers	Results: Facilitators
						High level of mistrust of the CBHIS administrators by the respondents<br><br>**Financial Sustainability**<br>CBHIS members' contribution could only fund 10% of the total running costs while the rest came from donor's subsidies. This means that without the subsidies, the premiums would not be able to run the scheme sustainably<br><br>**Financial protection**<br>The community in the study area is grossly exposed to potential financial ruin as a result of out-of-pocket mode of payment for health care<br><br>**Package content**<br>The main disadvantage reported by current CBHIS members was lack of certain essential services at the clinic such as inpatient and X-ray components	

Item	Objective	Methods	Population	Results: Description of package or scheme	Results: Socio-demographic factors	Results: Barriers	Results: Facilitators
						**Attitude Factors**<br>Non-members did not see any incentive to subscribe, since they felt CBHIS members did not receive preferential treatment over non-members<br><br>**Consumer understanding of concept of health insurance**<br>The concept of fund pooling was found to be poorly understood by the community<br><br>**Amount and timing of premium**<br>Even for those community members who were well aware of the existence of the insurance scheme, they felt that the cost of being a member was way too high although there is an option of paying in instalments<br><br>**Accessibility to facility**	

Item	Objective	Methods	Population	Results: Description of package or scheme	Results: Socio-demographic factors	Results: Barriers	Results: Facilitators
						Geographical Coverage<br>Almost 30% of the respondents reported long distance to health facility as the reason for not seeking health care compared to the national average of 18%.<br><br>**Community involvement**<br>Lack of incorporation of community members in the management of the CBHIS	
**Author & year**<br>Rao et al. 2012<br><br>**Funding**<br>NR<br><br>**Study design**<br>Quantitative (cross-sectional survey)	The purpose of the evaluation of the Rajiv Aarogyasri community health insurance scheme (RAS) was to provide insights into the current performance of the scheme, to examine whether it is meeting the overall objectives and to suggest ways by which it may be further strengthened.	**Sampling size and method**<br>217; random sampling<br><br>**Time frame**<br>NR (documentary analysis is from April 1, 2007 to September 30, 2008)<br><br>**Data collection**<br>Documentary analysis, surveys, semi-structured interviews<br><br>**Data analysis**	**Sample population**<br><br>Beneficiaries and stake holders – state government, Aarogyasri Health Care Trust, Start Health Insurance Company<br><br>**Setting**<br>Andhra Pradesh, India	**Type:** Community health insurance scheme<br>**Content:** NR<br>**Population covered**: Families below poverty line (BPL)<br>**Enrolment rate:** NR<br>**Unit of Enrollment:** NR<br>**Source of fund:** NR<br>**Premium:** NR<br>**Cost-sharing:** Yes (3600 INR = USD 77.3)<br>**Role of government:** NR		**Accessibility to facility**<br>*Geographical Coverage*<br>With increasing distance to major cities, the utilization rate declined<br><br>**Marketing and promotion strategies**<br>*Adequacy of marketing campaign*<br>The beneficiary satisfaction survey elicited the lowest scores for information provided about the scheme<br><br>**Cost sharing**	**Facility-related factors**<br>*Facility environment*<br>The beneficiary satisfaction survey elicited the highest score for cleanliness<br><br>**Human resource planning and management**<br>The beneficiary satisfaction survey elicited the highest scores for doctors and nurses

Item	Objective	Methods	Population	Results: Description of package or scheme	Results: Socio-demographic factors	Results: Barriers	Results: Facilitators
		NR		**Provider-payment method**<br>NR		Nearly 60% beneficiaries incurred a median out-of pocket expenditure of INR 3600 (USD 77.3) with transport, medicine and pre-diagnostic investigations being the major reasons.	
**Author& year**<br>Shaw**,** 2002<br><br>**Funding:**<br>NR<br><br>**Study design**<br>Mixed (qualitative and quantitative)	Assess quantitative and qualitative impacts of the CHF	**Sampling size and method**<br>NR<br><br>**Time frame**<br>1999<br><br>**Data collection**<br>2 surveys (1 quantitative and 1 qualitative: focus group)<br><br>**Data analysis**<br>NR	**Sample population**<br><br>NR<br><br>**Setting**<br>Tanzania (Igunga and Singida Ruraldistricts)	**Type:** Community Health Fund (CHF)<br>**Content:** A range of preventive and curative services<br>**population covered:** household in rural areas<br>**Enrolment rate:** between 4.9% and 5.9%<br>**Unit of Enrollment:** household<br>**Source of fund:**<br>(i) collect prepayments from households on a voluntary basis, (ii) receive a matching grant from government equal to the prepayments<br>**Premium:** Yes (1,000 shillings)<br>**Cost-sharing:** Yes (NR)		**Attitude Factors**<br>*Perception of scheme*<br>Suspicions were strong that government and providers wouldn't perform their expected role<br><br>**Amount and timing of premium**<br>Between wards, variations in enrolment rates over time, have also been attributed to varying agricultural performance since pre-payment contributions were made once yearly at harvest time<br><br>User fees were probably not high enough to motivate people to save and risk pool; therefore	**Government support**<br>Government was able to draw on funds from a World Bank loan for cost-sharing<br><br>**Financial sustainability**<br>With additional monies and "buying power", CHF is associated with improved access and quality improvements of publicly operated facilities<br><br>**Management/administrative structure**<br>Management of the Fund was grafted onto the existing District Health Management Committee. Only in this way was it possible to jointly sustain management

Item	Objective	Methods	Population	Results: Description of package or scheme	Results: Socio-demographic factors	Results: Barriers	Results: Facilitators
				**Role of government:** Partial financing		prepayment was not sufficiently attractive<br><br>**Package content**<br>Entitlements in the CHF benefit package were limited to preventive and selected curative care services at health centers or out-patient departments at local hospitals<br><br>**Management/administrative structure**<br>Included community representation but real autonomy of management was not possible and CHF had to be heavily mapped onto existing district health management arrangements controlled by government.	functions of CHF and assure public accountability to the satisfaction of the Ministry of Health
**Author & year**<br>Mladovsky, 2014<br><br>**Funding:** Stewart Halley Trust	To explore why people drop-out of CBHI schemes<br>To explore the relationship between CBHI membership, active community participation and social capital (drop-	**Sampling size and method (9)**<br>382 households (227 members and 155 ex-members)	**Sample population:** Households (current members and ex-members)<br><br>**Setting**<br>Senegal	**Type:** CBHI<br>**Content:** NR<br>**population covered:** NR<br>**Enrolment rate:** 4%<br>**Unit of Enrolment:** **Household**	***Economic status***<br>The results indicate that although members of the CBHI scheme were wealthier and had higher expenditure levels than ex-members the	**Health status**<br>Member households were twice as likely to have had an illness, accident or injury OR=2<br>Members had nearly twice as likely to have a	**Accessibility of facility**<br>*Geographical Coverage*<br>Members were more than twice as likely to be situated closer to a health service provider

Item	Objective	Methods	Population	Results: Description of package or scheme	Results: Socio-demographic factors	Results: Barriers	Results: Facilitators
**Study design**<br>Quantitative	out and active community participation)	Disproportionate stratified random sampling<br><br>**Time frame:**<br>March to August 2009<br><br>**Data collection**<br>Survey<br><br>**Data analysis**<br>Logistic regression		**Source of fund:**<br>**Premium:** Present but amount NR<br>**Cost-sharing:** NR<br>**Role of government:**<br>NR	difference was not statistically significant.<br>*Per capita expenditure*<br>Members of the CBHI scheme had higher expenditure levels than ex-members but the difference was not statistically significant.<br>*Demographics, education, ethnicity, religion*<br>The odds ratios for the demographic, education, ethnicity and religion variables were also not significant, except for age	disability, than ex-member households, OR=1.74 pointing to adverse selection<br><br>**Trust in scheme**<br>The majority of people who dropped out of CBHI did not take up opportunities to actively participate, did not trust the scheme staff or leaders, felt they were not able to hold the CBHI scheme to account<br><br>**Social Solidarity**<br>The majority of people who dropped out of CBHI did not know many other members and did not believe that CBHI promotes solidarity	Members were three times more likely to report that health care access is an advantage of membership OR=3.05<br><br>**Attitude factors**<br>*Perceived quality of services*<br>Members had a much higher probability of reporting that the quality of health service providers was satisfactory<br><br>**Community involvement in decision-making**<br>Active community participation is negatively correlated with drop-out; the more active the mode of participation, the stronger was the correlation<br><br>**Attitude factors**<br>*Satisfaction with services*<br>Members being more likely than ex-members to rate the operation of

Item	Objective	Methods	Population	Results: Description of package or scheme	Results: Socio-demographic factors	Results: Barriers	Results: Facilitators
							the scheme as excellent or satisfactory<br><br>**Attitude factors**<br>*Trust*<br>Nearly 70% of scheme members reported that the scheme managers or leaders were trustworthy<br><br>*Sense of ownership of scheme*<br>Members being more likely than ex-members to think they could influence scheme operation<br><br>***Social Solidarity***<br>Members were more likely to have more solidarity than ex-members<br><br>**Relative relations**<br>Members being more likely than ex-members to have heard of the scheme from a family member or friend compared to another source.

Item	Objective	Methods	Population	Results: Description of package or scheme	Results: Socio-demographic factors	Results: Barriers	Results: Facilitators
**Author & year**<br>Atim et al. 2001<br><br>**Funding**<br>USAID<br><br>**Study design**<br>Case Study	This part of the report is based on an inventory of mutual health organizations (MHOs) in the country plus some illustrative case studies of typical MHO models in Ghana; This paper assembles and analyzes information concerning existing and prospective health financing innovations in the public, private, and community sectors	**Sampling size and method**<br>NR<br><br>**Time frame**<br>March to June 2001<br><br>**Data collection**<br><br>Survey<br><br>**Data analysis**<br>Descriptive statistics	**Sample population**<br>Household<br><br>**Setting**<br>Sagnerigu Health Sub district, in the Tamale district. Ghana	**Type:** Nkoranza Community Health Financing Scheme (provider initiated, covering hospitalizations and moving towards community co-ownership)<br>**Content:**<br>All services associated with hospitalization, including: Drugs; Consultations; X-Ray; Laboratory tests; Admission fees; Complicated delivery; Surgery; Referral; OPD cases involving snakebite.<br>**Population covered**<br>People of Nkoranza district<br>**Enrolment rate:** over 48,000 member<br>**Unit of Enrollment:**<br>Whole family registration.<br>3 months registration period<br>**Source of fund:**,<br>Membership contributions;		**Consumer understanding of concept of health insurance**<br>Failure of people to understand the concept of risk sharing, thus tending to withdraw from the scheme after a few years of not benefiting from the package<br><br>**Moral hazard**<br>Some moral hazards still bewilder the scheme, arousing serious concerns<br><br>**Attitude factors**<br>*Perception of scheme*<br>Perceptions of some members about the quality of care they receive as compared to others<br><br>**Management/administrative structure**<br>Inadequate autonomy hinders Scheme's ability to negotiate quality and	

Item	Objective	Methods	Population	Results: Description of package or scheme	Results: Socio-demographic factors	Results: Barriers	Results: Facilitators
				Christian Charity Non-Governmental Organization (first 3 years)<br>**Premium:**<br>¢12,000 per member per<br>year<br>**Cost-sharing:** NR<br>**Role of government:** Community initiated/managed and highly adapted to context and skills of illiterate and very poor rural community<br>**Provider-payment method:** Fee-for-Service basis		cost of care with provider.	
**Author & year**<br>Atim et al. 2001<br><br>**Funding**<br>USAID<br><br>**Study design**<br>Case Study	This part of the report is based on an inventory of mutual health organizations (MHOs) in the country plus some illustrative case studies of typical MHO models in Ghana; This paper assembles and analyzes information concerning existing and prospective health financing innovations in the public,	**Sampling size and method**<br>NR<br><br>**Time frame**<br>March to June 2001<br><br>**Data collection**<br><br>Survey<br><br>**Data analysis**	**Sample population**<br>Households<br><br>**Setting**<br>Ghana	**Type:** Dodowa Community Health Insurance Scheme (purely provider-initiated and managed)<br>**Content:**<br>Free OPD care;<br>Transport provided for acute emergencies;<br>Basic lab tests;<br>Antenatal care;<br>Delivery & postnatal care; Family Planning;		**Accessibility of facility**<br>*Geographical coverage*<br>Difficulty in access to remote areas due to lack of transport<br><br>**Financial sustainability**<br>Unforeseen financial implications which had not been budgeted for	

Item	Objective	Methods	Population	Results: Description of package or scheme	Results: Socio-demographic factors	Results: Barriers	Results: Facilitators
	private, and community sectors	Descriptive statistics		Child Welfare &Immunization; Referral<br>**Population covered**<br>People of Dangme West district<br>**Enrolment rate:** Total membership between 4,000 – 5,000<br>**Unit of Enrollment:**<br>Selective family registration attracts double premium payment<br>3 months registration period<br>**Source of fund:**, (mainly donor) financial investment<br>**Premium:**<br>¢12,000 for people aged 6– 69 years (non-exemptables)<br>¢6,000 for Children (0-5years) & the elderly 70+years<br>-¢24,000 for non-group Family members<br>**Cost-sharing:** NR<br>**Role of government:**<br>NR		**Human resource planning and management**<br>Non availability of permanent staff which made the health staff combine their normal district work with those of the scheme<br>Some of the above problems (and the low enrolment rates) point to possibly more fundamental issues<br><br>**Management/administrative structure**<br>Inexperience of the district medical scheme spearheading the project and lack of requisite managerial skills for running an insurance scheme<br><br>**Marketing and promotion strategies**<br>Possibly inappropriate social marketing and community mobilization technique	

Item	Objective	Methods	Population	Results: Description of package or scheme	Results: Socio-demographic factors	Results: Barriers	Results: Facilitators
				**Provider-payment method:** Fee-for-Service basis		**Stakeholder involvement** Inadequate levels of community involvement and ownership	
**Author & year** Atim et al. 2001 **Funding** USAID **Study design** Case Study	This part of the report is based on an inventory of mutual health organizations (MHOs) in the country plus some illustrative case studies of typical MHO models in Ghana; This paper assembles and analyzes information concerning existing and prospective health financing innovations in the public, private, and community sectors	**Sampling size and method** NR<br>**Time frame** March to June 2001<br>**Data collection** Survey<br>**Data analysis** Descriptive statistics	**Sample population** Household<br>**Setting** Sagnerigu Health Sub district, in the Tamale district. Ghana	**Type:** Tiyumtaaba Community Health Financing Scheme (purely community initiated/managed) **Content:** Inpatient bills; Drugs; Ambulance/transport; Delivery; Laboratory **Population covered** People of Sagnerigu Health Sub district, in the Tamale district. **Enrolment rate:** Currently 8 of the 22 communities in this sub district. **Unit of Enrollment:** Membership is compulsory for all community members, once a community decides to join. **Source of fund:**, -Cash payment by the Association directly to		**Accessibility of facility** *Geographic coverage* Transport and logistics for the imitators who are facilitating the expansion to cover other communities that have expressed interest to join. The cost of hiring a means of transport in times of emergency is very high and is a major drain on their resources in the fund<br>**Accessibility of facility** *Referral system* The lack of a nearby health facility to give first line treatment to members before transferring to hospital only when necessary is also a major concern and challenge	**Payment arrangement for services** Repayment is flexible and can be paid in instalments<br>**Membership criteria** Generally, adverse selection, which is considered a common risk, associated with most MHOs is very minimal with this scheme, due to the idea of compulsory membership by every member of the community<br>**Community Involvement** The entire village has a common understanding that people should pay when they can do so, under their own free will

Item	Objective	Methods	Population	Results: Description of package or scheme	Results: Socio-demographic factors	Results: Barriers	Results: Facilitators
				the health facility in case of admissions.<br>-Cash disbursement to family of an admitted member presenting prescriptions from an approved health facility.<br>-Cash advance to members in case of labor, snakebite, transport and other life threatening conditions.<br>**Premium:** ¢1000/month<br>**Cost-sharing:** NR<br>**Role of government:** Community initiated/managed and highly adapted to context and skills of illiterate and very poor rural community<br>**Provider-payment method:** Fee-for-Service basis		**Political economy context**<br>The falling value of the Cedi and the dollarization of the economy is a threat to the survival and growth of the fund<br><br>**Financial Sustainability**<br>Future rises in health care costs will make it difficult for the association to meet the needs of its members in situations where people will present ill at the same time, or in serious cases of illness such as conditions requiring surgery	since they all have no steady source of income. In this scheme, there is hardly any defaulting, for though people may not have been regular at meetings, they still send their contributions<br><br>**Attitude Factors**<br>*Trust*<br>A great deal of trust has been put in the treasurers and it is believed by the community members that "only the devil can influence the treasurer negatively"
**Author & year**<br>Basaza et al, 2010<br><br>**Funding**<br>DGIC Belgium and ITM Belgium for supporting this study.	The objective of the present study is to determine the level of knowledge and understanding of CBHI and the perception of its relevance among key	**Sampling size and method**<br>Of 64 senior MOH staff, 29 were interviewed. Purposeful sampling was used in selecting staff from among the five	**Sample population**<br>District Health Officers (DHOs) and senior staff of the Ministry of Health (MOH).	**Type:** Community health insurance. All the existing schemes were based on or linked to private not-for-profit sector health facilities<br>**Content:**		**Consumer understanding of concept of health insurance**<br>There is lack of understanding of the principles of insurance	**Attitude factors**<br>*Perception of scheme*<br>This study indicates that CBHI is perceived as being a relevant policy option for Uganda; more specifically it is seen as a

Item	Objective	Methods	Population	Results: Description of package or scheme	Results: Socio-demographic factors	Results: Barriers	Results: Facilitators
**Study design**<br>Qualitative	policy makers and district health service managers in Uganda.	major departments at the Ministry of Health headquarters<br>Simple random sampling of 43 DHOs from a sample frame of 73 districts without CBHI schemes was carried out. The nine DHOs in districts with existing schemes were later interviewed<br>**Time frame**<br>Second half of 2007<br>**Data collection**<br>Semi-structured interviews<br>**Data analysis**<br>The qualitative data were analyzed using the framework method, facilitated by EZ-Text software	**Setting**<br>Uganda	NR.<br>**Population covered:** Protection against catastrophic health expenditure for both formal and informal sectors.<br>**Enrolment rate:** NR<br>**Unit of Enrollment:** NR<br>**Source of fund:**, 58% coming from out-of-pocket expenditure, 22% from the government and the remaining 20% from donors<br>**Premium:** NR<br>**Cost-sharing:** NR<br>**Role of government:** CBHI started jointly by the Ministry of Health and donors, primarily the Department for International Development of UK (DFID) and United States Aid for International Development (USAID).		by staff of the MOH headquarters and DHO, such as the expectation of benefit even if not ill<br>**Cost-sharing**<br>Out-of-pocket expenditure remains an important feature of health care financing in Uganda despite blanket abolition of user fees in government facilities<br>**Marketing and promotion strategies**<br>There has never been any specific national conference, guidelines or deliberate attempt by the MOH to promote CBHI in public units. This may explain the low level of knowledge of CBHI<br>**Facility-related factors**<br>Interviewees think that basic medicines and other medical supplies are lacking in public facilities<br>**Attitude factors**	potential source of funds and as a means of raising the quality of care. Respondents may feel that the quality of care will be improved due to increased availability of health workers and medicines.

Item	Objective	Methods	Population	Results: Description of package or scheme	Results: Socio-demographic factors	Results: Barriers	Results: Facilitators
				**Provider-payment method:** Fee-for-Service basis		*Perception of scheme*<br>Interviewees think quality of care is poor in public facilities<br><br>**Payment arrangement for services**<br>There is a generalized practice of under-the-table payments. This may indicate that the policy of abolition of user fees may not have led to the desired improvements in health care delivery<br><br>**Accessibility of facility**<br>*Geographical coverage*<br>Most of the health services are located in urban areas and offer poor quality services, whereas the majority of CBHI members live in the rural areas<br><br>**Political economy context**<br>Community health insurance is sometimes a controversial and politically sensitive issue	

Item	Objective	Methods	Population	Results: Description of package or scheme	Results: Socio-demographic factors	Results: Barriers	Results: Facilitators
						in Uganda, where user fees have been abolished in the public sector following a decision by the president.<br><br>**Government support**<br>Absence of clear national guidelines on health care financing hindered scheme progress	
**Author & year**<br>Ron, 1999<br><br>**Funding**<br><br>**Study design**<br>Case study		**Sampling size and method**<br>NR<br><br>**Time frame**<br>March to June 2001<br><br>**Data collection**<br>Not reported<br><br>**Data analysis**<br>Descriptive	**Sample population**<br>Household<br><br>**Setting**<br>Guatemala and Philippine	**Type:** community health insurance scheme<br>Scheme of the Association por Salud de Barillas (ASSABA) in Guatemala<br>ORT health status plus scheme (OHPS) in Philippine<br>**Content:**<br>Catastrophic medical expenditures at tertiary level care<br>**Population covered**<br>low and unstable income families in rural areas<br>**Enrolment rate:** over 48,000 member		**Management/administrative structure**<br>The scheme in Guatemala was not sufficient established as an administrative body at the conceptual stage. By the time the necessary action was taken, local conflicts hindered progress<br><br>**Personal predisposition**<br>*Affordability of care*<br>Fluctuation in memberships may result from a change in household expenditure priorities in the holiday season	**Management/administrative structure**<br>The major success factor are probably the sound administrative structure provided by a cooperative and controls in the delivery system and in expenditures, through the salaried primary health care team, referral process, and the capitation agreement for hospital-based services<br><br>**Relative relations**<br>Membership registration increased after learning about the inpatient admission of an insured

Item	Objective	Methods	Population	Results: Description of package or scheme	Results: Socio-demographic factors	Results: Barriers	Results: Facilitators
				**Unit of Enrollment:** Whole family registration.<br>3 months registration period<br>**Source of fund:**, Membership contributions; Christian Charity Non-Governmental Organization (first 3 years)<br>**Premium:** ¢12,000 per member per year<br>**Cost-sharing:** NR<br>**Role of government:** Community initiated/managed and highly adapted to context and skills of illiterate and very poor rural community<br>**Provider-payment method:** Fee-for-Service basis		**Consumer understanding of concept of health insurance**<br>Fluctuation in memberships may result from the lack of familiarity with the insurance concept<br><br>**Amount and timing of premium**<br>The mechanism of subsidized contributions for specific very low or unstable income families over a limited period may not be the best way to sustain membership.<br><br>**Stakeholder involvement**<br>The slow and problematic development of the scheme in Guatemala was influenced by local factors including conflict over health care provisions by the church-affiliated institutions	resident of the community<br><br>**Marketing and promotion strategies**<br>Registration increased following competivie promotion campaigns at community level and spreading of word of positive experience<br><br>**Human resource planning and management**<br>The ORT MCC Project and its Cooperative Management team became increasingly interested in achieving success in the scheme and understood the basic underlying objectives of enabling access to health care in the target population<br><br>**Package Content**<br>Periodic attention should be given to adjusting and extending benefits to deal with the changing

Item	Objective	Methods	Population	Results: Description of package or scheme	Results: Socio-demographic factors	Results: Barriers	Results: Facilitators
							needs and preferences of the insured population<br><br>**Amount and timing of premium**<br>Contributions should also need to be adjusted at reasonable intervals to reflect changes in benefits changes in health costs and inflations<br><br>**Community involvement**<br>The cooperative framework with its built-in members' participation mechanisms, appears to be a major factor in finding the optimal administrative base for such voluntary scheme than a one-sided decision by a health care financing scheme without member or community participation
**Author & year**<br>Ouimet,2007	The objectives of this study are to evaluate if there is a gap between	**Sampling size and method**	**Sample population**<br>Household	**Type:** Community-based Health Insurance<br>**Content:**	*Education; Marital status; Economic status;*	**Community involvement**	**Package Content**<br>Registration is on family basis. This means that,

Item	Objective	Methods	Population	Results: Description of package or scheme	Results: Socio-demographic factors	Results: Barriers	Results: Facilitators
**Funding**<br>International Development Research Centre for its financial support of the primary study<br><br>**Study design**<br>Mixed methods	CBHI subscribers' values and those of their promoters, and to determine the characteristics of subscribers and their CBHI schemes	Qualitative: 12 subscribers, 24 leaders, 12 local policy-makers and 24 administrators, were selected by purposeful sampling. Quantitative: A random sample of 394 subscribers was selected from 46 community CBHIs to complete a survey<br><br>**Time frame**<br>2002<br><br>**Data collection**<br>Focus groups with subscribers (n=12 groups), as well as semi-directed interviews with leaders (n=24), local policy-makers (n=12) and administrators (n=24), selected by purposeful sampling. A random sample of 394 subscribers was also selected from 46 community CBHIs to complete a survey<br>**Data analysis**	**Setting**<br>Senegal	Both outpatient services offered at primary-care facilities (CSPS) and up to 15 days inpatient care at the district hospital (CMA) are covered. All essential medicines offered at public facilities are also included in the insurance package. Members are assigned to one public facility based on geographical location<br>**Population covered**<br>only about 3% of the total sample population<br>**Enrolment rate:** about 10%<br>**Unit of Enrollment:**<br>Household<br>**Source of fund:**,<br>Membership contributions;<br>Christian Charity Non-Governmental Organization (first 3 years)<br>**Premium:**<br>Membership fees for children under 15 years are 500 francs CFA	Respondents enrolled in CBHI had significantly more **schooling** and were more literate; they are also more likely to be **married**, and live in smaller, less **wealthy** households.<br>*Religion*<br>Muslims were also more likely to enroll.<br>*Age Gender*<br>Age and being female were not statistically significant	Implementation has been slow and laborious. More attention should be given to increasing member participation in the processes involved in implementing CBHI<br><br>**Financial sustainability**<br>Irregularity of contributions is seen as the greatest threat to sustainability<br><br>**Health status**<br>Individuals reporting a health problem over the 30 day period preceding the interview were more likely to be enrolled in CBHI<br><br>**Stakeholder involvement**<br><br>In Senegal, more attention should be given to reducing the gap between promoters' and subscribers' values. Reducing the gap between subscriber and	once a person decides to join, then all members of that family must register. This is to avoid the risk of adverse selection. A family card is issued to a family that has registered, with personal data of each member of the household provided on the form, as well as a photograph of each member<br><br>**Social solidarity**<br>Content analysis of the qualitative data shows that subscribers consider solidarity as the most important aspect of CBHI

Item	Objective	Methods	Population	Results: Description of package or scheme	Results: Socio-demographic factors	Results: Barriers	Results: Facilitators
		content analysis of the focus group and interview transcription; multilevel logistical regression modelling using Hierarchical Linear Model for factors associated with subscribers' answer		($USD 1), while adults (ages 15+) pay 1500 francs CFA ($USD 3)<br>**Cost-sharing:**<br>There is no co-payment, ceiling or limit on number of services rendered<br>**Role of government:**<br>**Provider-payment method:**<br>Primary and secondary-care facilities that operate within the CBHI implementation zone sign two-year contracts with the insurance scheme, and are paid by the CBHI on an annual capitation basis.		promoter expectations may help increase enrolment in, and performance of CBHI	
**Author & year**<br>Basaza, 2008<br>**Funding**<br><br>**Study design**<br>Qualitative	To investigate people's current perceptions of CBHI in both schemes and reasons for the low enrolment	**Sampling size and method**<br>30 initial focus group discussions and 18 in-depth interviews were held for both schemes Sub-pop 1–4 were randomly drawn from a list of existing community groups within a scheme,	**Sample population**<br>Members and non-members of CBHI schemes<br>**Setting**<br>Uganda	**Type:** Two Community Health Insurance: the Ishaka scheme, a typical example of a provider-based CBHI scheme and the Save for Health Uganda (SHU) scheme, a community run model<br>**Content:**		**Household dynamics**<br>*Household size*<br><br>One of the reasons most mentioned for not joining the schemes is being unable to pay contributions for their large families: "I want to join but paying for my 10 children is a problem	**Stakeholder involvement**<br>In the Save for Health Uganda (SHU) scheme, members vote, set their own rules as members and there are no complaints<br><br>**Package Content**

Item	Objective	Methods	Population	Results: Description of package or scheme	Results: Socio-demographic factors	Results: Barriers	Results: Facilitators
		whereas sub-pop 5 was chosen from a list of existing groups in one of the randomly drawn villages without plan members using the village register.<br>. Interviewees were randomly selected from a household list of women, widowers, orphans, the disabled and elderly in each sub-pop<br>**Time frame**<br>2005-2006<br>**Data collection**<br>Focus group discussions and in-depth interview<br>**Data analysis**<br>content analysis of the focus group and interview transcription; multilevel logistical regression modelling using Hierarchical Linear Model for factors associated with subscribers' answer		Most of the schemes provide in-patient and out-patient care including deliveries at the facility where the scheme is based or the facility contracted by the scheme to provide services.<br>**Population covered**<br>The Ishaka CBHI scheme consists of 15 groups, with a total membership of 950 people out of a population of 50,000 people within the catchment area.<br>Save for Health-Uganda (SHU) scheme acts as an umbrella group for CBHI sub-schemes in the area (currently 13). The total number of beneficiaries in scheme as of September 2006 was 3624 people, a rate of about 6% of the catchment population.<br>**Enrolment rate:**<br>The premium for 3 months is Ushs1 15,000 for a family of 4 and		**Consumer understanding of concept of health insurance**<br>A large section of the communities poorly understand the concept of pooling contributions. It is only those who are members that have a relatively better understanding of pooling. One of the reasons provided for not joining is that participants do not see how to benefit if they do not fall sick<br>**Marketing and promotion strategies**<br>Not adequate sensitization has been done and the content of the sensitization needs to be tailored to the core principles of CBHI<br>**Attitude factor**<br>*Affordability of care*	In the Save for Health Uganda (SHU) scheme treatment is provided for all diseases, which encourages enrolment

Item	Objective	Methods	Population	Results: Description of package or scheme	Results: Socio-demographic factors	Results: Barriers	Results: Facilitators
				Ushs 3700 for an additional person. (1 US$ 1 = Ushs 2,000). The contribution per individual member of a family in the SHU scheme amounts to on average Ushs 3800 as an initial payment, and about Ushs 800 per annum.<br>There is no rule on the level of the premium<br>**Unit of Enrollment:** Household<br>**Source of fund:**, Membership contributions;<br>Christian Charity Non-Governmental Organization (first 3 years)<br>**Premium:** Membership fees for children under 15 years are 500 francs CFA ($USD 1), while adults (ages 15+) pay 1500 francs CFA ($USD 3)..<br>**Cost-sharing:** There is no co-payment, ceiling or limit on		Inability to pay for membership was pointed out as the foremost reason for not joining the two schemes.<br><br>**Membership criteria**<br>One of the reasons for not joining the scheme is that people failed to raise the required number in the group/village<br><br>**Membership criteria**<br>The exclusion of treatment of chronic diseases in the Ishaka benefits package of the schemes comes out as a contributing cause to low enrolment. People with chronic diseases are the people in most need and should be reached by the plan as much as possible<br><br>**Stakeholder involvement**<br>In the Ishaka scheme, members were not involved in the decision-making process on the scheme. A majority of	

Item	Objective	Methods	Population	Results: Description of package or scheme	Results: Socio-demographic factors	Results: Barriers	Results: Facilitators
				number of services rendered<br>**Role of government:**<br>**Provider-payment method:** Primary and secondary-care facilities that operate within the CBHI implementation zone sign two-year contracts with the insurance scheme, and are paid by the CBHI on an annual capitation basis		the respondents had a feeling, that they are not involved, which discouraged enrolment<br><br>**Facility-related factors**<br>There is no modern equipment in Ishaka Hospital compared to private clinics. Absence of some prescribed medicines also come up as causes of low enrolment in the Ishaka scheme<br>Other quality issues raised were the hospital is dirty and long queue<br><br>**Accessibility of facility**<br>Long distance from the communities to provider health facilities is a factor in both schemes	
**Author & year**<br>Basaza, 2007<br><br>**Funding**<br>International Development Research Centre for its	To explore factors on both demand and supply side of health care delivery that can explain the low enrolment in scheme	**Sampling size and method**<br>62 individuals were recruited for Key Informant interviews (KI) at National and district and Exit	**Sample population**<br>Key informant interviews:<br>-Hospital and scheme level (Medical Directors, Superintendents, other	**Type:** Community-based Health Insurance<br>Ishaka: Provider driven<br>SHU: community-run<br>**Content:**<br>Ishaka:		**Membership criteria**<br>Difficulties for existing community groups to raise 60% of the membership or 100 families per village prior to enrolment	**Stakeholder involvement**<br>In SHU, decisions are taken jointly by the scheme members and by the hospital concerned

Item	Objective	Methods	Population	Results: Description of package or scheme	Results: Socio-demographic factors	Results: Barriers	Results: Facilitators
financial support of the primary study<br><br>**Study design**<br>Mixed methods		Interviews (EI) at hospital levels<br>Exit interviews were carried out with all the scheme members who visited the hospitals during the period of data collection. Selection of non-scheme members involved every second exit patient who qualified as a non-scheme member from within the catchment area the hospital<br><br>**Time frame**<br>November 2004–December 2005<br><br>**Data collection**<br>Review of the schemes' records, key informant interviews and exit polls with both insured and non-insured patients.<br>**Data analysis**<br>The framework method was used for the data analysis	managerial, scheme staff).<br>-District level (DDHS, Secretaries of Health).<br>-National level Health Planners, Development Partners, WHO country office, Religious Bureaus, UCBHFA staff, average duration in post 5 years<br>Exit interviews from both schemes:<br>Patients who are scheme members<br>Patients who are non-scheme members<br><br>**Setting**<br>Central and Southern Uganda	Inclusion: Inpatient and outpatient care<br>Exclusions: Chronic diseases, dental and optic care<br>Kiwoko: Inclusion: In-patient and outpatient care<br>Exclusion: Chronic condition<br>**Population covered**<br>**Enrolment rate:**<br>In 2005:<br>Ishaka: 2%<br>Kiwoko: 6%<br>**Unit of Enrollment:**<br>**Source of fund:**<br>Initially by UK bilateral aid agency to Uganda, but support stopped in 2002<br>**Premium:**<br>Ishaka**: $8**<br>SHU**: $2**<br>**Cost-sharing:**<br>Co-payment OPD (US$)<br>Ishaka: 0.5<br>SHU: Varies per sub-scheme<br>**Role of government:**<br>Initially developed by government, but today		**Consumer understanding of concept of health insurance**<br>Lack of information on and poor understanding of the notion of community health insurance<br><br>**Personal pre-disposition**<br>*Trust*<br>Lack of trust in local financial organizations after previous depressing experiences with similar institutions<br><br>**Amount and timing of premium**<br>Problems in the ability to pay the premium influenced enrolment<br>Both schemes do not have health care subsidies for the poorest sectors of the population<br><br>**Capacity of insurance promotors**	**Consumer awareness of scheme**<br>Interviewees got to know the scheme through sensitization by the scheme staff, scheme members and local churches<br><br>**Attitude factors**<br>*Sense of ownership of scheme*<br>A majority of the SHU scheme members interviewed were involved in the mobilization of scheme members<br><br>**Personal pre-disposition**<br>*Affordability of care*<br>Affordable health care paid for in a convenient way encouraged enrolment<br><br>**Attitude factors**<br>*Consumer perception of scheme*<br>The major reasons for joining both CBHI schemes were to make it

Item	Objective	Methods	Population	Results: Description of package or scheme	Results: Socio-demographic factors	Results: Barriers	Results: Facilitators
				the schemes receive no direct financing from governments or donors. The government is still involved in basic management training and program to raise community awareness<br>**Provider-payment method:**<br>NR		Limited expertise on CBHI within the Ministry of Health and amongst donors. There was little or no practical experience in setting up CBHI schemes affected low enrolment<br><br>**Stakeholder involvement** Nothing is done to ensure that fund managers account to scheme members<br><br>**Community involvement in decision-making**<br>Low level of community involvement in the management of hospital-based CBHI schemes<br><br>**Marketing and promotion strategies**<br>A lack of information by health professionals (health workers, district services managers and health planners) contribute to low enrolment	easy to access health care, receive subsidized and prompt treatment<br><br>**Amount and timing of premium**<br>Payment by installment was an important enabling factor to enrolment

Item	Objective	Methods	Population	Results: Description of package or scheme	Results: Socio-demographic factors	Results: Barriers	Results: Facilitators
						**Human resource planning and management**<br>Poor interest in and understanding of the notion of CBHI by health professionals (health workers, district services managers and health planners) affect low enrolment<br><br>**Government support**<br>The government is to provide policy, legislative, technical and regulative support and control. Additionally, it should be a financier of CBHI schemes especially for the indigent.	
**Author & year**<br>Criel, 2003<br><br>**Funding**<br>German bilateral co-operation GTZ (Gesellschaft für Technische Zusammenarbeit) and the Institute of Tropical	The research team formulated six hypotheses, which might explain why the insurance scheme failed to attract more subscribers	**Sampling size and method**<br>Three focus groups were organised for each sub-group. Twelve participants were selected per group<br>Sub-group 1: the 555 people who subscribed	**Sample population**. People selected from four different patient sub-groups. Updated household lists and the members' lists of the MHO were used as the basis for selection of both villages and respondents	**Type:** Maliando Mutual Health Organisation<br>**Content:** Benefit package included free access to all first line health care services (except for a small co-payment), free paediatric care, free emergency surgical care		**Amount and timing of premium**<br>There is a problem of affordability for many poor who cannot raise enough money to pay the subscription<br><br>**Household dynamic**<br>*Household size*	**Consumer understanding of concept of health insurance**<br>The great majority of research subjects, members and non-members alike, acquired a very accurate understanding of the

Item	Objective	Methods	Population	Results: Description of package or scheme	Results: Socio-demographic factors	Results: Barriers	Results: Facilitators
Medicine in Antwerp (Raamakkoord DGIS-ITG, Eigen Initiatief N° 9.630)<br><br>**Study design**<br>Qualitative study		during two consecutive years (1998 and 1999).<br>Sub-group 2: the 843 people who cancelled their subscription after the first year.<br>Sub-group 3: the 474 people who only subscribed during the second year.<br>Sub-group 4: people who did not subscribe at any time during the period 1998–1999.<br>Four additional focus group discussions—one for each sub-group—were organised to validate the findings of the first 12 focus groups<br><br>**Time frame**<br>March 2000.<br><br>**Data collection**<br>Focus group discussions<br>**Data analysis**<br>Content analysis of the focus group and interview transcription; Multilevel logistical regression modelling using Hierarchical	**Setting**<br>Guinea-Conakry (West Africa)	and free obstetric care at the district hospital. Also included were part of the cost of emergency transport to the hospital<br>**Population covered**<br>**Enrolment rate:** 6%<br>**Unit of Enrollment:** household<br>**Source of fund:** Membership contributions<br>**Premium:** Annual insurance fee of about US$2 per individual<br>**Cost-sharing:** Small co-payment (amount not specified)<br>**Role of government:** None<br>**Provider-payment method:** NR		All respondents who mention large families say that the household subscription is too large a burden for big families to bear<br><br>**Management/Administrative structure**<br>Participants mentioned the need for opportunities for appealing against a negative decision or event that are included in constitution of their associations.<br><br>**Membership criteria**<br><br>While all respondents seem to be aware that the household and not the individual is the subscription unit, this requirement is only perceived as a restriction of membership Not a single respondent referred to the link between mandatory subscription of the entire household and the need	concepts and principles underlying health insurance<br><br>**Community involvement in decision-making**<br>The intense period of preparation and the genuine sense of community participation that was incorporated from the start have substantially contributed to strong impression of trust and also helped develop confidence in the capacities of local managers<br><br>**Social solidarity**<br>The transfer of funds from those who have remained healthy to those who have used the service is recognized as the fundamental principle of the scheme<br><br>**Marketing and promotion strategies**<br>People reported adhering to the scheme because of

Item	Objective	Methods	Population	Results: Description of package or scheme	Results: Socio-demographic factors	Results: Barriers	Results: Facilitators
		Linear Model for factors associated with subscribers' answer				to avoid adverse selection.<br><br>The unit of subscription that is most convenient, and the decision on who should or should not be considered as members of a household should be discussed with the target population<br><br>**Perception of scheme**<br>Poor quality of care at the health centre is the main criticism that people have of the scheme. It is a fact that, when quality of care is perceived as unsatisfactory, people will not be motivated to join the scheme<br><br>**Facility-related factors**<br>The absence of recovery from illness is often linked to ineffective drugs dispensed at the centre. The quality of the products is not good; patients receive drugs that do not make them	convincing information campaigns<br><br>**Financial sustainability**<br>People reported adhering to the scheme because they believe the insurance helps preserve health and provides financial accessibility

Item	Objective	Methods	Population	Results: Description of package or scheme	Results: Socio-demographic factors	Results: Barriers	Results: Facilitators
						better; patients always receive the same drugs even for different illnesses<br><br>**Stakeholder involvement**<br>In Guinea-Conakry, poor involvement of health professionals in scheme design contributed to low support of scheme implementation	

Item	Objective	Methods	Population	Results: Description of package or scheme	Results: Socio demographic factors	Results: Barriers	Results: Facilitators
Author & year<br>Noubiap , 2013<br><br>**Funding**<br>Not reported<br>Study design<br>quantitative descriptive cross-sectional study<br><br>**Study design**<br>Qualitative	Evaluating the informal sectors workers' knowledge, concern and preferences on CBHI schemes and their financial plan to cover health costs	**Sampling size and method**<br>Convenient sampling of 160 workers<br><br>**Time frame**<br>January 2010<br><br>**Data collection**<br>interviews using a structured pretested questionnaire containing both coded and open-ended questions	**Sample population**<br><br>informal sectors workers<br><br>**Setting**<br>Bonassama health district (BHD) of Douala – Cameroon	**Type:**<br>Community-based health insurance (CBHI)<br>**Content:**<br>Not reported<br>**population covered:**<br>not reported<br>**Enrolment rate:** 1.2% of the sample size had CBHI<br>**Unit of Enrollment:**<br>not reported<br>**Source**<br>**Fund:** beneficiaries		**Marketing and promotion strategies**<br>Poor awareness about the scheme from the worker's point of view.<br>This low awareness of CBHI schemes is probably due to inadequate public sensitization through the mass media<br><br>**Membership criteria**<br>Decreased enrolment rate (effective social	**Consumer awareness of scheme** Awareness of CBHI schemes was significantly associated with a high level of education<br><br>**Consumer understanding of concept of health insurance**<br>One hundred and thirty-eight (86.2%) respondents thought that belonging to a

Item	Objective	Methods	Population	Results: Description of package or scheme	Results: Socio demographic factors	Results: Barriers	Results: Facilitators
		**Data analysis**<br>descriptive analysis		**Premium:** yes 704 CFA francs (1.39 USD) per person<br>**Cost-sharing:** not clear<br>**Role of government:** not clear<br>**Provider-payment method**<br>Not reported		marketing strategies must be implemented through information-education and communication on CBHI schemes)	CBHI scheme could facilitate their access to adequate health care, and were thus willing to be involved in CBHI schemes<br><br>**Management/administrative structure**<br>53.3% preferred management by missionaries, our respondents were significantly in favor of this management by missionaries<br><br>**Amount and timing of premium**<br>75.4% of respondents preferred monthly premiums. Those who preferred monthly premiums declared it will be a lot easier to pay monthly premiums than to pay a bulk sum once a year<br><br>**Social solidarity**<br>A hundred and ten (68.7%) respondents

Item	Objective	Methods	Population	Results: Description of package or scheme	Results: Socio demographic factors	Results: Barriers	Results: Facilitators
							belonged to a solidarity based community association. The majority of these people (86.9%) said they would accept to fuse their individual associations to create a CBHI scheme. Belonging to a solidarity based community association could reflects one's willingness to join a CBHI.
**Author & year**<br>Nsiah-Boateng and Aikins, 2011<br><br>**Funding**<br>Self-finance by the authors<br><br>**Study design**<br>Cross sectional survey (quantitative)	To assess the performance of the Ga District Mutual Health Insurance Scheme over the period 2007-2009.	**Sampling size and method**<br>376 households using a multi- stage sampling method<br><br>**Time frame**<br>2007-2009<br><br>**Data collection desk review**<br>Household survey (self-administered semi-structured questionnaire**)**<br><br>**Data analysis**<br>Descriptive analysis	**Sample population**<br>Membership data of Ga DMHIS and selected heads of surveyed households in the Madina Township**.**<br><br>**Setting**<br>Ghana	**Type:** Mandatory National health insurance scheme - District Mutual Health Insurance Scheme (DMHIS**)**<br>**Content:** not reported<br>**population covered:** everyone<br>**Enrolment rate:** 21.8%<br>**Unit of Enrollment:** not clear<br>**Source of fund:** national health insurance authority, donors, internal funds		**Amount and timing of premium**<br>The premium is very expensive, this effects the enrolment negatively<br><br>**Package Content**<br>Content of health benefit package Population did not register since the package does not provide the services needed<br><br>**Consumer understanding of concept of health insurance**	

Item	Objective	Methods	Population	Results: Description of package or scheme	Results: Socio demographic factors	Results: Barriers	Results: Facilitators
				**Premium: yes GH¢ 10.00 to GH¢24.00**<br>**Cost-sharing**: no<br>**Role of government:** not clear<br>**Provider-payment method:** diagnostic related groupings		Community members do not understand the importance of an insurance scheme which decreases enrolment rates<br><br>**Financial sustainability**<br>Decreased contributions of the informal sector can affect the sustainability of the insurance scheme<br><br>**Cost-sharing**<br>No co-payment causes an increase in medical bills expenditure which in turn could pose problems in the sustainability of the scheme<br><br>**Accessibility of facility**<br>Hospital is too far<br><br>**Attitude factors**<br>*Satisfaction with services*<br>Members who are not satisfied from the scheme are less likely to renew their membership	

Item	Objective	Methods	Population	Results: Description of package or scheme	Results: Socio demographic factors	Results: Barriers	Results: Facilitators
**Author & year**<br>Onwujekwe, 2009<br><br>**Funding**<br>the UK Department for International Development (DFID)<br><br>**Study design**<br>Quantitative	To determine how equitable enrolment and utilization of community-based `health insurance is in two communities with varying levels of success in implementing the scheme.	**Sampling size and method**<br>971 respondents<br>Simple random sampling<br><br>**Time frame**<br><br>Not reported<br><br>**Data collection**<br><br>pre-tested interviewer administered questionnaire<br><br>**Data analysis**<br>descriptive analysis	**Sample population**<br><br>The heads of households or their representatives<br><br>**Setting**<br>Anambra state, southeast Nigeria	**Type:** community based health insurance<br>**Content**: not reported<br><br>**population covered:** not reported<br>**Enrolment rate:** not reported<br>**Unit of Enrollment:** household<br>**Source of fund:** beneficiaries<br>**Premium:** yes - 100 Naira per adult per month and 50 Naira per child per month<br>**Cost-sharing:** not reported<br>**Role of government:** A publicly owned primary Healthcare (PHC) center in each community served as the focal health facility for the scheme. The state government was expected to refurbish and equip the health facilities involved in the scheme, as well as make matching contributions to the		**Membership criteria**<br>Members can only enroll if they are part of a 500 population community which affects success in implementing the scheme.<br><br>**Financial sustainability**<br>The low level of premium as well as the low level of fund pools found in the study will not improve equity in financing and may not lead to sustainability of the scheme<br><br>**Attitude factors**<br>*Satisfaction of enrollees*<br>Not being satisfied with skill of staff was mentioned by respondents in both communities as what they did not like about the scheme<br><br>**Accessibility of facility**<br>*Geographical coverage*<br>Facilities were very far so population did not register<br><br>**Facility-related factors**	**Consumer understanding of concept of health insurance**<br>Awareness of the scheme offered financial risk protection. Most respondents who registered in both communities did so because they perceived that the scheme offered financial risk protection<br><br>**Personal pre-disposition**<br>*Affordability of care*<br>Affordability of care in the healthcare facilities with the development of the scheme. The most important reason indicated for the increase use of facilities was that cost of care through the scheme was affordable.<br>An important reasons given for willingness to renew registration in Igboukwu was

Item	Objective	Methods	Population	Results: Description of package or scheme	Results: Socio demographic factors	Results: Barriers	Results: Facilitators
				premiums paid by the householders to the scheme. In addition, the state government paid the salaries of the health care providers.<br>**Provider-payment method**<br>Not reported		Facilities were not equipped to provide health care.<br>Lack of drugs, delay in services, long waiting hours, inconvenient facility environment, payment for treatment in some cases and not being satisfied with skill of staff.<br>6.3% of respondents from Igboukwu and 14.8% from Neni stated poor staff attitude as a reason for not registering with scheme<br>**Human resource planning and management**<br>Staff incompetence in the facilities.<br>Poor staff attitude<br><br>Unavailability of doctors was mentioned by most respondents in both communities as what they did not like about the scheme. 22.9% of those respondents unwilling to renew from Igboukwu	affordability of health care services<br>**Attitude factors**<br>*Perception of scheme*<br>An important reasons given for willingness to renew registration in Igboukwu was good quality care offered<br><br>**Accessibility of facility**<br>*Geographical coverage*<br>Nearness of health facility, adequacy of facilities as reasons for their increased facility utilization<br><br>**Human resource planning and management**<br>Availability of health personnel as reasons for their increased facility utilization<br><br>**Package content**<br>The most reported benefit of CBHI was ready availability of outpatient services in both communities

Item	Objective	Methods	Population	Results: Description of package or scheme	Results: Socio demographic factors	Results: Barriers	Results: Facilitators
						indicated the absence of a doctor at the facility as a reason<br><br>**Attitude factors**<br>*Trust*<br>Some people did not register because they did not trust the insurer in regulating the funds<br><br>**Amount and timing of premium**<br>Cost of registration was high<br>Premium is retrogressive meaning the poor is affected by the premium amount leading to inequity<br><br>**Accessibility of facility**<br>*Geographic coverage*<br>Those who did not register because they felt the provider facility was too far were 20 (14.1%) in Igboukwu<br><br>**Personal predisposition**<br>19.6% and 16.3% of respondents who knew	

Item	Objective	Methods	Population	Results: Description of package or scheme	Results: Socio demographic factors	Results: Barriers	Results: Facilitators
						about CBHI in Neni and Igboukwu did not register because they claimed they were registered with private and government-run insurance schemes, respectively.	
**Author & year**<br>Ozawa and Walker, 2009<br><br>**Funding**<br>UK Department for International Development (DFID)<br><br>**Study design**<br>Mixed method	To understand the role and influence of villagers' trust for the health insurer on enrollment in a community-based health insurance (CBHI) scheme in Cambodia.	**Sampling size and method**<br>74 participants through snowballing sampling for the qualitative part of the study<br>Cluster random sampling (n=560) for the quantitative part of the study on household surveying. Which combined a stratified sampling of 360 participants on insurance status and a population-proportional-to-size sampling of 200 participants.<br><br>**Time frame**<br>Not reported<br><br>**Data collection**<br>Focus groups and household surveys	**Sample population**<br>community members in Thmar Pouk operational district in Banteay Meanchey province above 18 years of age, without a health care provider or an employee of the health insurance organization in the family.<br><br>**Setting**<br><br>Northwest Cambodia	**Type:** voluntary community based health insurance<br>**Content:** all primary health care and hospital costs at public facilities<br>**population covered:** all community members<br>**Enrolment rate:** 25–30 percent<br>**Unit of Enrollment:** family-based<br>**Source of fund:** beneficiaries<br>**Premium: yes -** premium of US$2.00 per person per year and up to US$12.00 per family per year<br>**Cost-sharing:** none<br>**Role of government:** unclear<br>**Provider-payment method**		**Amount and timing of premium**<br>Timing of collecting the contributions (e.g., monthly, quarterly, annually) was important for the community members to get their CBHI cards before they pay for any premium	**Attitude factors**<br>*Trust*<br>Trust in the 5 domains (organizational trust, financial trust, honesty, competence, and personal interactions) had increased the enrolment rate for the CBHI. Villagers who renewed the insurance scheme were found to have statistically significantly higher trust levels compared to those who were new to the scheme

Item	Objective	Methods	Population	Results: Description of package or scheme	Results: Socio demographic factors	Results: Barriers	Results: Facilitators
		**Data analysis**<br>inductive and deductive methods.<br>Multinomial Logistic Regression<br>Factor analysis		Not reported			
**Author & year**<br>Rao et al. 2009<br><br>**Funding**<br>NR<br><br>**Study design**<br>Quantitative	To provide an evaluation of the Community health fund (one kind of community based health insurance) program's performance on certain key parameters: enrolment, cost recovery, financial protection, service utilization and community perceptions of the program	**Sampling size and method**<br>Random sampling (baseline and follow up surveys)<br>166 households in the baseline study<br>320 households in the follow up study<br><br>**Time frame**<br><br>2004-2006<br><br>**Data collection**<br>reports from routine project monitoring, the health management information system (HMIS), and household surveys of facility catchment	**Sample population**<br>household<br><br>**Setting**<br>Afghanistan (Parwan, Saripul, Wardak and Nimroz)	**Type:** voluntary community based health insurance (community health fund)<br>**Content:** covered all services offered at the designated health facility in addition to inpatient care at the nearest district hospital.<br>**population covered:** not reported<br>**Enrolment rate:** 1%-38%<br>**Unit of Enrollment:** household<br>**Source of fund:** beneficiaries (premium plus users fees)<br>**Premium:** yes – 6$ but varies according to the household's economic status and number of members.		**Consumer awareness of scheme**<br>unaware of the program (house visits and active campaigns for enrolment awareness)<br><br>**Amount and timing of premium**<br>High premiums decreased enrolment rate. Moreover, annual premium did not present an adequate financial incentive to enroll. (To encourage enrolment, future CHF programs would do well to set the premium at levels which offer substantial financial incentives to households.)<br>The inconvenient timing of the enrolment<br><br>**Facility-related factors**	**Stakeholder involvement**<br>Active and continuous engagement of program implementers with community members

Item	Objective	Methods	Population	Results: Description of package or scheme	Results: Socio demographic factors	Results: Barriers	Results: Facilitators
		**Data analysis**<br><br>Descriptive analysis		Poor households were enrolled free of charge<br>**Cost-sharing:** yes co – payment (only for funding members) which is 0.02$<br>**Role of government:** Governance<br>**Provider-payment method**<br>Not reported		Perceived low quality of services at the CHF clinics in terms of lack of drugs<br><br>**Attitude factors**<br>*Trust*<br>Lack of faith in the resident doctor was among the top three reasons why households did not enroll.<br><br>*Perception of scheme*<br>Generally bad service quality<br><br>**Financial allocation**<br>Preference for pay-for-service when sick<br><br>**Membership criteria**<br>enrolling households at the clinic instead of in their villages	
**Author & year**<br>Schneider, 2005<br><br>**Funding**<br>Not reported<br><br>**Study design**	This study examines trust-building structures and practices in MHI in Rwanda. It aims at to identifying whether interviewees raise trust and trust-related issues in	**Sampling size and method**<br>24 focus groups<br><br>**Time frame**<br><br>August 2000	**Sample population**<br>Healthcare providers, MHI managers, MHI members, non-members	**Type:** Micro-health insurance (MHI)<br>**Content:** preventive and curative care in health centers and ambulance transport to the district hospital		**Stakeholder involvement and relations**<br>That MHI are co-managed by representatives from providers and the population was generally appreciated. However,	**Community involvement in decision-making**<br>Community member involvement in the development of the MHI scheme enhances

Item	Objective	Methods	Population	Results: Description of package or scheme	Results: Socio demographic factors	Results: Barriers	Results: Facilitators
qualitative study	their views of the MHI system; second, factors in the consumer–provider–MHI relationship that affect consumer trust in MHI; and third, whether consumer trust in MHI affects the decision to enroll MHI.	**Data collection**<br>focus groups<br><br>**Data analysis**<br><br>Descriptive analysis	**Setting**<br><br>Rwanda rural district	**population covered:** everyone<br>**Enrolment rate:** 19%<br>**Unit of Enrollment:** not reported<br>**Source of fund:** beneficiaries<br>**Premium:** yes<br>**Cost-sharing:** yes- co-payment per episode of illness, and user fees for hospital care not covered by MHI (i.e. drugs, surgery).<br>**Role of government:** governance and delivery<br>**Provider-payment method**<br>Capitation		two provider groups in low-enrolment areas proposed that "MHI should be managed by providers because the population trusts providers"<br><br>**Management/administrative structure**<br>To respond to consumer needs and patient concerns, members and non-members suggested ''MHI managers must be close to the population, learn about people's problems, and inform people about MHI; and they must defend members' interest when negotiating with providers for better quality care''<br><br>**Human resource planning and management**<br>Insured and uninsured respondents in both areas complained about technical incompetence:	consumer trust in MHI which in turn affects the decision to enroll MHI.

Item	Objective	Methods	Population	Results: Description of package or scheme	Results: Socio demographic factors	Results: Barriers	Results: Facilitators
						''Health facilities are dirty, lack qualified personnel, drugs, ambulances, clean bedding and electricity''<br>According to non-members: ''Health centres need to be adequately equipped in order for people to trust that MHI improves access to care''	
**Author & year**<br>Derriennic, 2005<br>**Funding**<br><br>US Agency for International Development (USAID)<br><br>**Study design**<br><br>Qualitative study	To examine good practices/models and key obstacles to sustainability in terms of governance and management, financial management and viability, risk management, marketing and membership incentives, community buy-in, and impact on quality of life of members.	**Sampling size and method**<br>All 12 currently functioning schemes in Uganda and one recently dissolved scheme<br><br>**Time frame**<br><br>**27 September to 7** October 2004<br><br>**Data collection**<br>interviews and focus group supplemented by documentary analysis<br><br>**Data analysis**	**Sample population**<br><br>CBHF scheme managers, current scheme members, and former members<br><br>**Setting**<br><br>Uganda	**Type:** community-based health financing (CBHF) schemes (all CBHF schemes are facility based, i.e., health care facilities both administer the schemes and provide the health care offered through the scheme)<br>**Content:** a range of benefits offered by the different CBHF schemes. Most schemes exclude chronic conditions, self-inflicted injury, optical care, and dental care and delivery services from the benefits package.		**Accessibility of facility**<br>*Geographical coverage*<br>Distance to the facility as one of the main hindrances to benefiting from the scheme<br><br>**Financial sustainability**<br>Facilities hosting poorly performing CBHF schemes currently in debt bear the burden of absorbing scheme deficits which could be a potential barrier to future scheme expansion<br><br>**Stakeholder involvement**<br>The perceived value of the health schemes can be shaken when there is low	**Facility-related factors**<br>Expanding the pool of affiliated providers so that members can obtain outpatient care at clinics closer to their homes saving them both time and transport costs<br><br>**Financial sustainability**<br>Can be achieved through successfully identifying a target population and set appropriate premiums and co-payments to attract members, contributing to cost recovery and overall financial viability

Item	Objective	Methods	Population	Results: Description of package or scheme	Results: Socio demographic factors	Results: Barriers	Results: Facilitators
		Content analysis		population covered: members as part of an already formed group (social groups: community groups, employer groups, and school groups<br>**Enrolment rate:**<br>**Unit of Enrollment:** household<br>**Source of fund:**<br>**Premium:** premiums must be paid for a minimum of four household members<br>**Cost-sharing:** Yes<br>**Role of government:** receives a grant from the Ministry of Health		community participation in decision making resulting in decreased support of the scheme a consequent decrease in scheme membership and cost recovery<br><br>**Consumer understanding of concept of health insurance**<br>Member dropout did not understand the purpose of the co-pay. Members of a now dissolved scheme expected to have their premiums returned if they did not access health services in a given quarter<br><br>**Management/administrative structure**<br>Lack of adequate financial accounting systems to provide proper separation of scheme accounts from the hospital's accounts prevents effective decision making by scheme managers	**Management/administrative structure**<br>CBHF scheme is treated as a cost center within the hospital. This allows the scheme to effectively examine its financial situation, including accounting for administrative cost in cost recovery calculations<br><br>**Marketing and promotion strategies**<br>A sensitized member base can make educated decisions to protect the financial viability of the CBHF scheme. This can help establish a strong membership base<br><br>**Cost sharing**<br>The community group's decision to raise the co-pay to combat overutilization of health services is a positive example of how a sensitized member base

Item	Objective	Methods	Population	Results: Description of package or scheme	Results: Socio demographic factors	Results: Barriers	Results: Facilitators
						**Marketing and promotion strategies**<br>No marketing research to show which marketing strategies can be successfully employed to increase interest in CBHF schemes and promote membership.	can make educated decisions to protect the financial viability of the scheme
**Author & year**<br>De Allegri , 2006<br><br>**Funding**<br>collaborative research grant SFB 544 of the German Research Society<br><br>**Study design**<br>Qualitative study	"To generate a comprehensive understanding of consumers' preferences for the specific elements of the scheme and their impact on decision to enroll"	**Sampling size and method**<br>32 stratified purposeful sampling design<br><br>**Time frame**<br>May and June 2004<br><br>**Data collection**<br>semi-structured interview<br><br>**Data analysis**<br><br>Thematic analysis	**Sample population**<br><br>Households heads<br><br>**Setting** Burkina Faso	**Type:** CBI scheme (voluntary enrolment)<br>**Content:** first-line and second-line medical services<br>**population covered:** household (adults and children**)**<br>**Enrolment rate: 4%**<br>**Unit of Enrollment:** household<br>**Source of fund:** community<br>**Premium:** Yes (set on an individual basis) plus an additional flat quota<br>**Cost-sharing: No**<br>**Role of government:** Government is the only provider of Western medical care in the area		**Membership criteria**<br>Definition of household does not adequately reflect all decision-making processes in individuals' everyday life. They pointed out that several adults often live with their wives and children within the same household, but can decide independently over the allocation of some economic resources available specifically to themselves and their direct dependent<br><br>**Amount and timing of premium**<br>Amount to be paid remains unaffordable for very poor households.	**Membership criteria**<br>The vast majority of respondents acknowledged the value of insuring the whole household as they recognized the possible limitations of a system which allows individual enrolment. They conceptualized these limitations not in terms of a possible adverse selection effect, but rather in relation to the equity and the completeness of financial protection that only household enrolment can secure.<br><br>**Amount and timing of premium**

Item	Objective	Methods	Population	Results: Description of package or scheme	Results: Socio demographic factors	Results: Barriers	Results: Facilitators
				**Provider payment methods:** Gate-keeping system<br>Providers paid by capitation		Current payment modalities, requiring that the premium is paid all at once for the entire household, constitute an important barrier to enrolment<br><br>**Facility-related factors**<br>Entire villages assigned to one specific local first-line facility and restricts access to services accordingly<br>They justified their reluctance to accept the facility imposed by CBI both in terms of the quality of care available and in terms of the overall social relations between their village and the village where the facility is located	Lower premium level for children compared to adults<br><br>**Management/administrative structure**<br>Setting a CBI management committee in each village was perceived as a guarantee for the well-functioning of the scheme and was judged to be socially and culturally appropriate<br>Decision to manage all funds in the District capital because they did not trust members of their own community to carry out the work adequately<br>Suggestions: provisions be made to grant households the possibility of diluting payment over a longer period of time in several instalments.
**Author & year**<br>Robyn, 2014	Understand how health workers perceive the current CBI provider	**Sampling size and method**	**Sample population**	**Type:** community-based health insurance		**Stakeholder involvement**	

Item	Objective	Methods	Population	Results: Description of package or scheme	Results: Socio demographic factors	Results: Barriers	Results: Facilitators
**Funding:** collaborative research grant SFB 544 of the German Research Society<br><br>**Study design**<br>Mixed methods	payment methods, the meaning health workers bring to the payment methods, and how they payment methods affect health worker motivation	23 for interview and 98 for survey<br>Stratified purposive sampling for qualitative and<br>Representative sampling for survey (All health workers employed at the 14 facilities that were contracted by the CBHI scheme)<br><br>**Time frame**<br>April 2010<br><br>**Data collection**<br>mixed-methods approach (in-depth interviews and a quantitative survey)<br><br>**Data analysis**<br>Thematic analysis for qualitative<br>Descriptive statistics	Health workers (nurses, midwives, and pharmacy managers) and the District Health Management Team<br><br>**Setting**<br>Burkina Faso	**Content:** The capitation paid to facilities covers only the cost of drugs prescribed to enrollees<br>**population covered:** children and adult<br>**Enrolment rate:**<br>**Unit of Enrollment:**<br>**Source of fund:**<br>**Premium:** Annual enrolment premium ($1 USD for children and $ 3 USD for adults)<br>**Cost-sharing:**<br>**Role of government:**<br>**Provider-payment methods:** Capitation		Conflict in physicians' roles to patient and facility they work in<br><br>**Financial sustainability**<br>Increased financial volatility of health facilities: The majority of head nurses raised the concern of facility bankruptcy, and the quality sacrifices they had to make to avoid bankruptcy<br><br>**Payment arrangement for services**<br>Capitation payment schedule generated substantial challenges for facilities, both due to the number of times payments that were made, as well as the month in which they were paid<br><br>**Human resource planning and management**<br>Method of provider payment used by the CBHI scheme caused	

Item	Objective	Methods	Population	Results: Description of package or scheme	Results: Socio demographic factors	Results: Barriers	Results: Facilitators
						health workers to feel that they could no longer fulfill their professional roles and responsibilities (role strain). As a consequence, health worker satisfaction, work-related motivation, and support for the CBHI were low.<br>The lack of an existing payment mechanism linked to CBHI enrollment was seen as a missed opportunity to align health worker incentives with the insurance scheme's objective of increasing CBHI coverage	
**Author & year**<br>Kyomugisha, 2009<br><br>**Funding**<br>Study design<br><br>**Study design**<br>Descriptive cross-sectional design employing qualitative techniques	To assess community perceptions of equity and sustainability in CHI schemes<br><br>To look at people's perceptions of equity when joining and accessing health care services in schemes and their perceptions of sustainability with regard	**Sampling size and method**<br>158 participants (from 15 focus groups) and 12 key informant interviews<br>Purposive sampling of categories of FGDs, random sampling of individuals within each FGD	**Sample population**<br>**FGDs:** CHI scheme members and non-members<br><br>**Interviews:** scheme managers, officials from Ministry of Health and one health financing organization	**Type:** community health insurance schemes<br>**Content:** Where treatment for diabetes, hypertension or major surgeries are not covered,<br>members are allowed to seek care from another source but payment will		**Package content**<br>Schemes refused to cover illnesses like diabetes and hypertension.<br><br>**Membership criteria**<br>Not allowing individuals without families to join.<br><br>For members to enroll, they had to be members of an already existing	**Membership criteria**<br>Non-discriminatory and voluntary joining of the schemes, allowing people to join irrespective of family background<br><br>**Stakeholder involvement**<br>Key informants observed that members'

Item	Objective	Methods	Population	Results: Description of package or scheme	Results: Socio demographic factors	Results: Barriers	Results: Facilitators
	to the role of CHI schemes	**Time frame**<br><br>**Data collection**<br>focus group and key informant interviews<br><br>**Data analysis**<br>Thematic analysis	**Setting**<br>Uganda	entirely be met by the patient<br>**population covered:** community members<br>**Enrolment rate:** low (no exact percentage)<br>**Unit of Enrollment:** enrolment was limited to four household members<br>**Source of fund:** NGO , member contributions, private hospital<br>**Premium:** flat premium and no exemptions<br>**Cost-sharing:** not reported<br>**Role of government:** not reported<br>**Provider-payment methods:** not reported		community-based organization and at least 60% of the organization's members had to join before they could start accessing health services.<br><br>Limit imposed on families, who may register no more than four members<br><br>**Amount and timing of premium**<br>The most vulnerable and needy in society such as orphans, the elderly and the disabled, are not exempt from payment, even though they usually have greater health needs than the rest of the population<br><br>**Financial sustainability**<br>They operate on small budgets, and lack government support<br><br>**Government support**<br>More support from both NGOs and government in	involvement in planning and decision making was crucial in sustaining CHI schemes.<br>Members are usually informed about already made decisions by the top management of the schemes, which was also revealed during FGDs.<br><br>**Sense of ownership of scheme**<br><br>Members' perceptions of sustainability covered sense of ownership of their health programs without it being forced on them

Item	Objective	Methods	Population	Results: Description of package or scheme	Results: Socio demographic factors	Results: Barriers	Results: Facilitators
						order to slowly move towards sustainability and sufficiently meet the health needs of the communities.	
**Author & year**<br>Parmar, 2012<br><br>**Funding**<br>Study design<br><br>**Study design**<br>Quantitative	To examine the change in adverse selection over time and second, evaluate the effect of targeted subsidies on adverse selection	**Sampling size and method**<br>**n = 6713**<br>Cluster randomized<br><br>**Time frame**<br>2004–07<br><br>**Data collection**<br>Focus group, survey, key informant interview<br><br>**Data analysis**<br><br>fixed effect models	**Sample population beneficiaries, households**<br><br>**Setting**<br>Burkina Faso	**Type: community-based health insurance (CBHI) schemes**<br>**Content:** first- and secondline medical services available within the NHD population **covered:**<br>**Enrolment rate:** 4–6.3%,<br>**Unit of Enrolment:** household<br>**Source of fund:** not reported<br>**Premium:** Annual premium is set on an individual basis (different for children and adults)<br>Premium subsidies were offered to the poor households<br>premium paid in one single installment, at the beginning of the year, after the harvest	**Socio-demographic factor-Economic status**<br>Individuals from low SES households were less likely to enroll.	**Health status**<br>The introduction of premium subsidies led to the insured group having significantly higher percentage of sick individuals, providing strong evidence for adverse selection, which put greater strain on financial viability of the scheme	**Amount and timing of premium**<br>Sick individuals who were offered subsidy had a higher probability to enroll compared to sick individuals who were not offered subsidy.

Item	Objective	Methods	Population	Results: Description of package or scheme	Results: Socio demographic factors	Results: Barriers	Results: Facilitators
				**Cost-sharing:** no copayments, deductibles or ceiling on the benefits.<br>**Role of government:**<br>**Provider-payment methods: Nor reported**			
**Author & year**<br>Hao, 2010<br><br>**Funding**<br>NR<br><br>**Study design**<br>Quantitative (cross-sectional survey)	To compare difference in services utilization and exploring major influencing factors on health service use of poor MFA enrollers between original benefit package and new package MFA project areas.	**Sampling method**<br>stratified cluster sampling method<br><br>**Time frame**<br>2004<br><br>**Data collection**<br>Interview<br><br>**Data analysis**<br><br>Two-level linear multilevel model and binomial regressions with a log link	**Sample size population**<br>625 and 869 respondents were included (age≥15) from two kinds of project towns.<br>Poor families who have been enrolled in MFA scheme in rural areas of ChongQing<br><br>**Setting**<br><br>China	**Type:** New benefit package under Medical Financial Assistance Scheme<br>**Content:**<br>inpatient service, some designated preventive and curative health services<br>vulnerable MFA cardholders<br>**Cost sharing**: Co-payment<br>**Role of government:**<br>Governance, delivery<br>**Provider-payment methods:** fee for service<br>**Rationale for Package:** Explore setting up health security system which directly targeting at the		**Cost-sharing**<br>Reimbursement rate of hospitalization increased from 40~70% to 60 ~ 80%, and for some special cases, services were free. But for most ordinary MFA cardholders, 20~40% co-payments for hospitalization costs still brought a huge economic burden to these poor families<br><br>**Payment arrangement for services**<br>*Re-imbursement policy*<br>Regulations of setting ceiling for reimbursement had limited poor families to benefit more from MFA	**Membership criteria**<br>"Compared with the original benefit package in H8 towns, the new benefit package of H8SP aimed at further improving the target population's accessibility to health services and overcoming the barriers existed in the pilot project areas through extending coverage of target population, covering out-patient services and reducing the copayment rate"<br><br>**Payment arrangement for services**<br>Adding out- patient reimbursement to the

Item	Objective	Methods	Population	Results: Description of package or scheme	Results: Socio demographic factors	Results: Barriers	Results: Facilitators
				poorest, find out an effective way of improving their accessibility and overall health status of poor populations, facilitate poverty reduction and promote sustain- able development in rural areas		**Package content**<br>Setting limitations on disease eligibility of MFA had limited poor families to benefit more from MFA<br><br>**Accessibility of facility**<br>*Geographical coverage*<br>Poor transportation and remote distance limited their health services utilization to some degree<br><br>**Moral hazard**<br>MFA package had significant association with hospitalizations.	benefit package of H8SP towns significantly increased MFA enrollee's accessibility to the basic health services<br><br>**Package Content**<br>The most important factor that influencing frequency of MFA use was type of benefit package, which suggested that poor families in H8SP towns had much higher frequencies of MFA use than those in H8 towns.
**Author & year**<br>Polonsky, 2008<br><br>**Funding**<br>DFID<br><br>**Study design**<br>Quantitative	To assess equity in access to health care within the scheme, compare the distribution of the subsidy between members and nonmembers in villages operating an insurance scheme, and to examine the probability of consulting in villages with and without a scheme.	**Sampling size and method**<br>9 scheme villages; 506 households<br>Random walk sampling<br><br>**Time frame**<br>July 2001<br><br>**Data collection**<br>Structured questionnaire	**Sample population**<br>Households<br><br>**Setting**<br>Rural Armenia	**Type:** CHI<br>**Content:** Basic drugs - PHC services at the local health post center, reproductive and maternal health care, and care for chronically ill patients<br>**Population covered:** May include vulnerable groups such as young children and elderly	*Age*<br>Utilization increases with increasing age, reflecting greater health needs among older individuals, with the odds of reporting an episode of ill-health increasing with age<br><br>Among women, the most frequent users are those over the age of 60, rather		

Item	Objective	Methods	Population	Results: Description of package or scheme	Results: Socio demographic factors	Results: Barriers	Results: Facilitators
		**Data analysis**<br>Mixed: Descriptive s and regression		**Enrolment rate:** Not reported<br>**Unit of Enrollment:** Household<br>**Source of fund:** Beneficiaries<br>**Premium:** Quarterly insurance premium of 1500 AMD (US$4.6)<br>**Cost-sharing:** Not reported<br>**Role of government:** Not reported	than those of reproductive age<br><br>*Gender*<br>Fifty-two per cent of women visited HPs compared with 45% of men, but among those primary respondents that did visit HPs at least once, men visited more frequently than women		

Item	Objective	Methods	Population	Results: Description of package or scheme	Socio-demographic Factors	Results: Barriers	Results: Facilitators
**Author & year**<br>Cofie, 2013<br><br>**Funding**<br>Heidelberg University, Graduate School of International Public health from the Research grant SFB 544 of the German Research Society (DFG).<br><br>**Study design**<br>Mixed: quantitative and qualitative	The study addressed three questions: To what extent did the IEC campaign enhance households' understanding of the CBHI scheme? To what extent did the IEC campaign influence households' enrolment in the CBHI scheme? Which IEC campaign components were important in enhancing knowledge and enrolment?	**Sampling size and method (9)**<br>250 households: Systematic random<br>22 key informants in decision-making positions: Purposive sampling<br>4 members of the CBHI project management team: purposive sampling<br><br>**Time frame**<br>July 2004<br><br>**Data collection**<br>Survey- in-depth interview - meeting<br><br>**Data analysis**<br>Mixed: Descriptive- regression	**Sample population**<br>250 households and 22 key informants in decision-making positions and 4 members of the CBHI project management team<br><br>**Setting**<br>Nouna, Burkina Faso	**Type:** CBHI<br>**Content:** NR<br>**Population covered:** Households<br>**Enrolment rate:** NR<br>**Unit of Enrollment:** Household<br>**Source of fund:** Premium<br>**Premium:** Yes<br>**Cost-sharing:** Not reported<br><br>**Role of government:** Not reported	*Education*<br>A positive association was found between educational status and level of knowledge about the CHI scheme, and between educational status and enrolment<br><br>*Occupational setting*<br>Residents in urban areas were 2.4 times less likely to have knowledge of the CHI scheme. Residing in the urban setting did not increase the odds of having knowledge of the scheme.<br><br>*Age*<br>Respondents aged between 36 and 54 years were positive	***Attitude Factors***<br>*Trust*<br>Lack of trust related to previous negative experiences with collective financial arrangements may affect CBHI enrolment<br><br>*Consumer perception of scheme*<br>Factors such as perceived poor quality of care and providers' resistance may affect decision to enroll in CHI	**Consumer awareness of scheme**<br>The relationship between awareness and enrolment was observed to be statistically significant. Of those who were exposed to the campaign, 79% (152) had adequate knowledge about the scheme, but just a little more than a third (35.3%) enrolled in the scheme<br><br>**Community involvement in decision-making**<br>Community leaders and CBHI Management asserted that the involvement of community heads and religious leaders was vital in the scheme promotion strategy. Of particular importance was the sense of ownership resulting from active engagement of community heads in the campaign.<br>Community participation was positive and significant determinants of enrolment

					and significant determinants of enrolment		***Marketing and Promotion Strategies***<br>*Campaign channel*<br>Exposure to multiple channels was the only significant and positive determinant of respondents' knowledge. Intensity of exposure to campaign channels was positive and significant determinants of enrolment.<br><br>***Relative Relations***<br>As indicated by community leaders, rural inhabitants tended to validate information from their relatives in urban areas. This means that a clear understanding of the CBHI concept by urban dwellers would be paramount to decision making and adoption of the initiative by many rural residents
**Author & year**<br>Jütting, 2004<br><br>**Funding**<br>ILO-STEP project<br>Study design<br><br>**Mixed**<br>(Quantitative and Case Study)	This chapter analyzes whether mutual health insurance schemes improve access to health care in rural Senegal. We tackle two principal questions: What are the important socioeconomic	**Sampling size and method**<br>346 (2900 people); two-stage sampling method<br><br>**Time frame**<br>March to May 2000	**Sample population**<br>Household<br><br>**Setting**<br>Villages (Fandène, Sanghé, Ngaye Ngaye, and Mont Rolland); Dakar, Senegal	**Type:** Mutual community health insurance scheme<br>**Content:** All except Ngaye Ngaye cover hospitalization; Ngaye Ngaye: primary health care	*Religion/Ethnicity*<br>At the household level, religion and ethnic identity also play an important role. The higher participation by Christians—the probability increases by nearly 40 percentage	**Management/administrative structure**<br>Management of the mutual, seemed to play a role. The mutual of Sanghé has faced several financial and managerial difficulties that led to a suspension of operations for some time. Consequently, several people left the mutual.	**Personal Pre-disposition**<br>Membership in other organizations, however, is a positive factor. People who have already experienced the advantages and disadvantages of being associated with local groups are obviously more disposed toward membership in a health insurance scheme

	determinants that explain membership in a voluntary health insurance scheme?	**Data collection** Survey, interview<br><br>**Data analysis** Regression analysis		**Population covered** Rural for the poor **Enrolment rate:** Fandène: 90.3% Mont Rolland: 62.6% Ngaye Ngaye: 81.5**%** Sanghé: 37.4% **Unit of Enrollment:** Household **Source of fund:** Beneficiaries, St. Jeande Dieu Hospital **Premium:** 100-200 F CFA; one-time membership card: 1000 F CFA (household head) **Cost-sharing:** Yes **Role of government:** None **Provider-payment method:** None	points compared with that for non-Christians<br><br>*Economic status* Lower income groups in the villages are significantly less represented in the mutuals. That means that the wealthy people in the communities are more likely to participate in the insurance schemes.<br><br>***Area of residence*** With respect to village effects, people living in Fandène have a higher effective demand for hospitalization than people in the other three communities	***Health status*** The probability for women and older people participating in scheme is higher than for men and younger persons in the household. It is reasonable to assume that women of child-bearing age and older people do need hospitalization care more often than other household members	**Social Solidarity** We assume that people acknowledging a high value of solidarity in their village tend to participate more<br><br>**Accessibility of facility** *Geographical Coverage* People living in Fandène have a higher effective demand for hospitalization than people in the other three communities. It is also the closest mutual to St. Jean de Dieu Hospital.
**Author & year** Schneider, 2004	The objective of this chapter is to respond to two	**Sampling size and method**	**Sample population** Households	**Type:** Community health insurance	*Gender* Insured households are more likely to	**Accessibility of facility** *Geographical Coverage*	**Membership criteria** The possibility of signing up in a CBHI plan as a family of

**Funding**<br>United States Agency for International Development (USAID)<br><br>**Study design**<br>Mixed (quantitative and case study)	questions about the pre- payment schemes' impact: What are the population groups that enroll in com-munity-based health insurance schemes? Does health insurance membership improve financial accessibility to care without increasing the burden of out-of-pocket health expenditures?	2518 households (11583 individuals); random<br><br>**Time frame**<br>October-November 2000<br><br>**Data collection**<br>Structured questionnaire; interview<br><br>**Data analysis**<br>Regression analysis	**Setting**<br>(Byumba, Kabgayi, Kabutare), Rwanda	scheme (mutual health association)<br>**Content:** All services and drugs provided in their preferred health center, including ambulance transfer to the district public or church-owned hospital, where a limited package is covered.<br><br>**Population covered**<br>Not reported<br>**Enrolment rate:** All three districts: 7.9%; Byumba: 10.6%<br>Kabgayi: 6.0%<br>Kabutare: 6.1%<br>**Unit of Enrollment:**<br>Household<br>**Source of fund:**<br>Beneficiaries, federation<br>**Premium:**<br>2500 francs (US$ 7.50)	be headed by a male individual<br><br>*Education*<br>Insured households are more likely to be headed by a male who has attended some schooling.<br><br>*Economic status*<br>Proportionally more CBHI member households are likely to come from higher income quartiles; other economic attributes, such as household cattle ownership and different income quartiles were not significant in the demand for health insurance	Distance to the health facility also seems to be an important criterion as almost 50 percent of the member households live within 15 minutes of the health facility. Membership begins to taper off as the distance to the health facility increases<br><br>**Cost-sharing**<br>In addition, out-of-pocket spending per episode of illness is significantly influenced negatively if patients live in the health center's vicinity and if they own cattle	up to seven members for the same annual premium might have been an incentive for larger households to enroll with all their family members. Proportionally more CBHI member households are likely to come from larger households<br><br>**Household Dynamics**<br>Households with five and more members are 60 percent more likely to buy insurance than smaller households<br><br>**Accessibility of facility**<br>*Geographical Coverage*<br>Households who live within 30 minutes of their health facility have a 296 percent higher probability of joining than those who live farther away. This latter result might have been influenced by health centers' and prepayment schemes' awareness campaigns, which could have been more intense in the vicinity of a health facility<br><br>***Marketing and Promotion Strategies***<br>*Campaign channel*

				**Cost-sharing:** Yes (US$ 0.30)<br>**Role of government:** Delivery, financing<br>**Provider-payment method** Capitation payment			Households who own a radio are 47 percent more likely to enroll than those without a radio, another result that might have been caused by the regular awareness campaigns transmitted by radio.<br><br>**Package content**<br>The health insurance schemes were “tailored” as desired by, and in response to, the needs of the local people<br><br>***Attitude Factors***<br>*Trust*<br>The main determinant of PPS participation is trust, which might be captured by the time variable. People living near the facilities are more likely to enroll because they know the health center personnel, as well as the prepayment scheme management team, and have been exposed to regular information campaigns on prepayment.<br><br>*Sense of ownership of scheme*<br>The participatory approach and the democratic management of PPS lead to sentiments of “ownership”

							and increased trust among the poor, which are basic conditions for poor households to engage in any investment<br><br>**Social Solidarity**<br>Local initiatives (churches and members who attended the PPS general assemblies) have helped to pay enrollment fees for indigents, widows, orphans, and poor high-risk patients such as HIV-positive individuals<br><br>***Personal Pre-disposition***<br>*Affordability of care*<br>Findings show that health insurance has tremendously improved the financial accessibility of its members to the modern health care system, particularly for women, children, and the poor. Access to care is determined by prepayment membership, patient age, pregnancy, patients' health status, distance to the health facility, and households' income group
**Author & year**<br>Ranson, 2004	This chapter assesses the impact of the Self-	**Sampling size and method**	**Sample population**<br>Household	**Type:**<br>Community	***Socio-economic status***	**Payment arrangement for services**	***Personal Pre-disposition***<br>*Affordability of care*

**Funding**<br>British Department for International Development (DfID)<br>**Study design**<br>Mixed (quantitative – cross-sectional cohort study and case study)	Employed Women's Association's (SEWA's) Medical Insurance Fund, Gujarat, in terms of inclusion of the poor, hospital utilization, and expenditure.	700 households; two-stage random cluster sampling<br>**Time frame**<br>February 14 – May 6, 2000<br>**Data collection**<br>Survey<br>**Data analysis**<br>Regression analysis	**Setting**<br>Gujarat, India	health insurance scheme<br>**Content:** Life insurance, medical insurance, and asset insurance Excluded from coverage under the Fund are certain chronic diseases (for example, chronic tuberculosis, certain cancers, diabetes, hypertension, piles) and "disease caused by addiction"<br>**Population covered**<br>Poor self-employed women between ages 18 and 58<br>**Enrolment rate:** NR<br>**Unit of Enrollment:** Household<br>**Source of fund:** Beneficiaries<br>**Premium:** 72.5 rupees	Controlling for other sociodemographic variables, older age was significantly associated with membership	Relatively few of those who were members of the Fund and were hospitalized were reimbursed through the scheme. This suggests that either women are not submitting claims even when they might be eligible for reimbursement or that the claims are not eligible for reimbursement<br><br>**Health status**<br>Controlling for other socio-demographic factors, higher frequency of illness episodes within the past month was significantly associated with membership in the Fund.	Hospital expenditures were significantly lower for pregnancy, delivery, or family planning than for other causes There were significant (but not consistently so) associations between SEWA membership and lower costs of hospitalization.

				**Cost-sharing:** Yes (% not reported) **Role of government:** None **Provider-payment method** NR			
**Author & year** Gumber, 2004<br><br>**Funding** Ford Foundation<br><br>**Study design** Quantitative	This chapter attempts to review existing community-based and self-financing health insurance schemes in India that serve the general population and address the needs of the poor and vulnerable. It discusses some critical issues concerning accessibility and use of health care services, out-of-pocket expenditure on health care, and the need for health insurance for poor rural and urban households pursuing varied occupations. The	**Sampling size and method** 1080; purposive sampling<br><br>**Time frame** 1998-1999<br><br>**Data collection** Survey<br><br>**Data analysis**<br><br>Descriptive statistics, regression analysis	**Sample population** Households<br><br>**Setting** Ahmedabad District, Gujarat. India	**Type:** Community health insurance scheme **Content:** SEWA includes inpatient medical care and coverage for gynecological ailments and occupational health-related diseases; maternity benefits; provides life and asset insurance for the woman and for her husband or, in the case of widowhood or separation, for other household members. **Population covered**	*Education* With level of education, the mean enrollment rate tended to decline. Education level turned out to be an insignificant predictor<br><br>*Occupational setting* The enrolment rate was found to be much lower among non-workers or subsidiary status workers than among home-based production or salaried workers<br><br>*Age* Among personal characteristics, the	***Household Dynamics*** *Household size* The rate tended to decline with the size of household. Household size showed an inverse relationship with enrollment, and the odds ratios tended to decline significantly in medium-size and large households<br><br>***Health status*** The enrolment rate was not higher among women who had reported delivery during the past two years.	**Accessibility of facility** *Geographical Coverage* The enrollment rate was higher among urban women than rural women, mainly due to better access to information as well as to the SEWA Bank, which manages the scheme.<br><br>***Health status*** There was no adverse selection in terms of whether the member had been suffering from any chronic ailment or had been hospitalized before. However, maternity, a predictable event, had increased the likelihood of enrollment to take advantage of a benefit allowance of Rs. 300 and coverage of the high risk of hospitalization. The enrolment rate was higher among women who had reported suffering from any

	chapter examines in detail the determinants of enrollment in the community-based financing scheme, using household-level data from the pilot study. It also investigates the issue of how much health insurance mitigates the households' burden of health care expenditure.			Poor self-employed women **Enrolment rate:** Not reported **Unit of Enrollment:** Household **Source of fund:** Beneficiaries, NGOs, government **Premium:** Rs. 60 **Cost-sharing:** Yes **Role of government:** Not reported **Provider-payment method** NR	mean enrollment rate was found to be higher in the middle age groups, 36–45 years and 46–55 years, than the other age groups. Among the personal attributes, the odds of being enrolled were five to seven times higher among middle-age groups than in the 16–25 years age group<br><br>*Marital status* The enrolment rate was also higher among currently married women.<br><br>*Economic status* The enrolment rate did not vary much across income quintiles, except in the top quintile, where it was marginally higher. Income was not found to be a significant predictor		chronic ailment or had been hospitalized in the previous 365 days.

**Author & year**<br>Supakankunti, 2004<br><br>**Funding**<br>The Health Insurance Office (MOPH)<br><br>**Study design**<br>Quantitative	"The objective of this chapter is to assess the application of voluntary health insurance, in this case the Health Card Program (HCP) of Thailand, and provide greater understanding of how a voluntary health insurance pro- gram performs and how to improve and sustain it more efficiently"	**Sampling size and method**<br>1000; not reported<br><br>**Time frame**<br>1994-1995<br><br>**Data collection**<br>Interview questionnaire; documentary analysis<br><br>**Data analysis**<br><br>Descriptive statistics, regression analysis	**Sample population**<br>Households; subdistrict and village leaders and volunteer health workers<br><br>**Setting**<br>Khon Kaen Province, Thailand	**Type:** Voluntary health insurance<br>**Content:** Outpatient care for illness and injuries, inpatient care, and mother and child health services<br>**Population covered**<br>Near-poor and middle-income class in rural areas or those who can afford the premium<br>**Enrolment rate:** 20%<br>**Unit of Enrollment:** Household<br>**Source of fund:** Beneficiaries, government (MOPH)<br>**Premium:** 1,000 baht (US$40)<br>**Cost-sharing:** Yes (Health service unit 80% Incentive & adm. 20% of total monetary amount of card sales revenue (the	*Economic status*<br>Income was not shown to be a strong determinant of card purchase.<br><br>*Employment*<br>The households that had a higher proportion of employed persons tended to purchase more cards than the households with a lower proportion.<br><br>*Education*<br>Those with lower levels of education tended to purchase cards, since lower education means lower income and thus usually not covered by any of the health insurance schemes	***Health status***<br>The research results show that the presence of illness was one of the significant factors related to card purchase and card utilization patterns<br><br>**Financial Sustainability**<br>The problem of card overutilization, confirmed in this study, has implications for the sustainability and efficiency of the program.<br>The results show that among card users 41.6 percent tended to visit health facilities more than before having a card, 48.4 percent the same as before, only 7.2 percent less than before, and 2.8 percent do not remember.<br><br>**Payment arrangement for services**<br>The existing program formula is set at 80 percent of the total monetary amount of card sales revenue. If no subsidy from the government was allocated to the providers, the expense would be borne by providers. There is a problem of equity since only the health cardholders are better off if community hospitals must	***Relative Relations***<br>The findings indicate that the continuity of card purchase in the study was associated with persuasion by a neighbor to buy a card<br><br>**Consumer awareness of scheme**<br>Cardholders had greater prior knowledge about the health card program before health officers explained it to them than did non-cardholders, and they had more satisfaction with the explanations from health officers.<br><br>*Satisfaction of enrollees*<br>The cardholder group had greater satisfaction with the health card than the non-cardholder group, and the cardholders tended to be satisfied with the price of the card more than were the noncardholders. This strongly explains the role of attitude.<br><br>**Cost sharing**<br><br>The introduction of cost-containment measures was highlighted as necessary to reduce escalating cost of

				formula differs slightly from year to year)<br>**Role of government:** Financing<br>**Provider-payment method** Not reported		subsidize cardholders by the former's own revenues.<br><br>**Accessibility of facility**<br>*Referral system*<br>Ineffective referral system gave rise to the problem of bypassing the health centers which in turn influenced program performance	medical claims and decrease overutilization of services, which in turn could pose threats to the sustainability of CBHI schemes
**Author & year**<br>Criel and Kegels, 1997<br><br>**Funding**<br>Not reported<br><br>**Study design**<br>Case study	To illustrate the feasibility of health insurance at the rural district to exemplify its managerial complexities and difficulties encountered in it evaluation.to focus on the evaluation of this scheme, on the conditions for reproducibility, and on avenues for future research.	**Sampling size and method**<br>Not reported<br><br>**Time frame**<br>Not reported<br><br>**Data collection**<br><br>Not reported<br><br>**Data analysis**<br><br>Not reported	**Sample population**<br>Not reported<br><br>**Setting**<br>Bwamanda district in North West Zaire	**Type:** Voluntary insurance scheme for hospital care<br>**Content:** Hospitalization<br>**Population covered:** All community members<br>**Enrolment rate:** 41% in 1994<br>**Unit of Enrollment:** Household<br>**Source of fund:** Beneficiaries and CDI (Centre de Développement Intégral)<br>**Premium:** Yes; 20 Zaires<br>**Cost-sharing:** Yes 20% copayment of hospital fee		**Amount and timing of premium**<br>Ready money is not present considering the region as a rural area (the collection of premiums took place during the time of year when the CDI buys the coffee and soy bean crop in the Bwamanda area)<br><br>***Moral Hazard***<br>Moral hazard whereby beneficiaries would indwell in activities with increased risk (co-payment).<br><br>***Political Economy Context***<br>Socio-economical unrest causes a fall in enrolment and funding	**Facility-related factors**<br>Having one hospital in the district allowed it to become a monopoly hospital business so people wanted to take part in the hospital insurance scheme<br><br>**Accessibility of facility**<br>*Referral system*<br>Referral and counter –referral systems between the different levels of care contributed to the effective and efficient functioning of the health services<br><br>**Attitude Factors**<br>*Trust*<br>Relationship of trust between the CDI and the population increased the enrolment rate of the population in the scheme<br><br>***Financial Sustainability***

				except for maternity services<br>**Role of government:** Unclear<br>**Provider-payment method** NR			The pooling of funds at the district level allows better risk sharing.
**Author & year** Supakankunti, 2000<br><br>**Funding** Not reported<br><br>**Study design** Quantitative	To examine the differing characteristics of card users and non-users, and also of card drop-outs and continuing card users and to examine the attitudes towards the (Health card program)HCP of card users and non-users at health centers and community hospitals (to assess the future potential of voluntary health insurance (the HCP) in Thailand by utilizing data collected in Khon Kaen Province, where the program	**Sampling size and method** 1000 households; not reported<br><br>**Time frame** 1994-1995<br><br>**Data collection**<br><br>Questionnaires and secondary data reports from each district<br><br>**Data analysis** Regression analysis Descriptive analysis	**Sample population** 1) Sub-district and village leaders, and volunteer health workers; (2) Households in the sample areas; (3) Health-care seekers, both card users and non-users<br><br>**Setting** Thailand - Khon Kaen	**Type:** Voluntary health insurance prepayment scheme<br>**Content:** Outpatient care for illness and injuries, inpatient care, and mother and child health services<br>**Population covered:** The near poor and middle-income class in rural areas or those who can afford the premium<br>**Enrolment rate:** 50.7%<br>**Unit of Enrollment:** Household	*Education* Educational level is a significant factor<br><br>*Economic status* Average household income per year is a significant factor<br><br>*Employment* Households with a higher proportion of employed persons tended to purchase more cards than households with a lower proportion.	**Health status** Presence of illness is a significant factor. Families with illness tend to purchase and repurchase the card The presence of illness was also significant between the health card dropout and continued card purchase groups. As expected, it was not significant between the health card non-purchase and the health card dropout groups.<br><br>**Accessibility of facility** *Geographical Coverage* Convenient access to a health center.Most employed people in rural areas are not covered by any health insurance scheme	***Attitude Factors*** *Satisfaction of enrollees* High level of satisfaction of enrollees implied higher levels of card purchase.<br><br>**Accessibility of facility** Health-care- seeking pattern among card users and non-card users strongly supports the importance of accessibility to health care among the card user group<br><br>**Package Content** Under the new criteria for card use (no limit on episodes and the first contact at either a health center or community hospital depending on convenient location), the rate of usage is increasing, especially at community hospitals.

	was recently implemented.)			**Source of fund:** Beneficiaries and general tax<br>**Premium:** Yes - 1000 baht<br>**Cost-sharing:** No<br>**Role of government:** Financing<br>**Provider-payment method** NR			
**Author & year**<br>Wang et al. 2006<br><br>**Funding**<br>Not reported<br><br>**Study design**<br>Quantitative	To examine adverse selection in a subsidized voluntary health insurance scheme, the Rural Mutual Health Care (RMHC) scheme, in a poor rural area of China.<br>"To examine if a subsidized community-based rural health insurance is a viable health care financing strategy in rural China since many efforts undertaken in the 1990s to	**Sampling size and method**<br>Follow up survey: 3492 rural residents in 1020 households; multistage random sampling<br><br>**Time frame**<br>2002-2004<br><br>**Data collection**<br>Data set from a baseline survey collected prior to the implementation of the study. Additionally a first year	**Sample population**<br>High risk population in rural areas (single elderly, the disabled, those with dementia, women who were pregnant in the previous year or at the time of interview, those admitted to the hospital in the previous year, or those with a severe health condition as diagnosed by the village doctor and	**Type:** Voluntary community health insurance scheme<br>**Content:** Not reported<br>**Population covered:** Everyone<br>**Enrolment rate:** 82% households<br>**Unit of Enrollment:** Household<br>**Source of fund:** Government and beneficiaries<br>**Premium:** Yes – 33-47 yuan<br>**Cost-sharing:** No	*Gender*<br>OR for gender is significantly smaller than 1, which implies that female residents are more likely to enroll in RMHC than male residents in partially enrolled households<br><br>*Age*<br>In the sub-sample model of individuals in partially enrolled households, the ORs for those aged 45–54 and aged 55+ are all significantly larger	**Membership criteria**<br>If enrolment unit is household-based, then we would lose healthy individuals in those partially enrolled households and the enrollment rate would drop.	**Amount and timing of premium**<br>Each participant is subsidized 18-22 yuan from the RMHC (rural mutual healthcare) study to participate in the scheme<br><br>***Government Support***<br>Government paid the full premium for those who cannot afford the premium<br><br>***Household Dynamics***<br>*Household size*<br>The OR for household size is significantly larger than 1. Residents with a large family are more likely to be enrolled in RMHC than the residents with a small family.

	reestablish CBHI failed."	evaluation survey took place.<br>**Data analysis**<br>Regression analysis	non-high risk individuals.<br><br>**Setting**<br>China	**Role of government:**<br>Financing and governance<br>**Provider-payment method**<br>Not reported	than 1, which implies that the older residents are more likely to enroll in RMHC than in young residents<br><br>*Education*<br>The OR for primary education is significantly smaller than 1, which implies that the residents with relatively higher education are more likely to enroll in RMHC than the residents with lower education		
**Author & year**<br>Kamuzora and Gilson, 2007<br><br>**Funding**<br>Regional Network for Equity in Health in Southern Africa (EQUINET)	To report the findings of a study that examined the factors influencing low enrolment in Tanzania's health prepayment schemes (Community Health Fund).	**Sampling size and method**<br>(National Level)<br>Interviews: four; not reported<br>(District Level)<br>Case studies: two districts; stratified purposeful sampling<br>Interviews: Council Health Service Board (four)	**Sample population**<br><br>National Level: Officials from the central Ministry of Health and the World Bank country office who were specifically responsible for CHF implementation.	**Type:** District-level voluntary prepayment insurance Scheme (CHF)<br>**Content:** Not reported<br>**Population covered:** Not reported<br>**Enrolment rate:** 10 %		**Financial Sustainability**<br>Since the number of households qualifying for exemption was large, exemption provision would erode the CHF's financial base; all blamed the central government for not addressing the financial sustainability of the CHF.<br><br>***Attitude Factors***<br>*Trust*	**Amount and timing of premium**<br>Households unable to pay premium fee are exempted from the premium

**Study design**<br>Qualitative and case study		Ward (four in each of three wards)<br>Community-level interviews in each of three villages (one from each ward): two FGDs; five interviews<br>→total of 13 FGDs (six in one district, seven in the other); 28 interviews (up to 5 randomly selected from each of three villages)<br><br>**Time frame**<br>Not reported<br><br>**Data collection**<br><br>- Documents (policy guidelines and evaluation reports) were collected and interviews conducted at	District Level: Council Health Service Board members (included Chairpersons and Secretaries)<br>Ward: chairperson and secretary of the Ward Health Committee (WHC) and two members of the Ward Development Committee (WDC)<br>FGDs with community residents<br>The 28 interviews with poor households<br><br>**Setting**<br>Tanzania	**Unit of Enrollment:** Household<br>**Source of fund:** Beneficiaries and Council Health Service Board<br>**Premium:** Yes<br>**Cost-sharing:** No<br>**Role of government:** Governance and delivery<br>**Provider-payment method**<br>Not reported		Lack of trust (managers lack transparency and are corrupt) in CHF managers influenced low enrolment<br><br>**Amount and timing of premium**<br>Inability to pay annual contributions is identified as an important barrier preventing poor households from joining the CHF<br><br>**Facility-related factors**<br><br>Problems with the quality of services identified included shortage of drugs and essential medical supplies and inappropriate diagnosis due to lack of diagnostic equipment. This was an important reason for low enrolment in the scheme<br><br>Limited range of services provided , coupled with referral problems; ward-level interviews in both districts identified instances where the district managers turned down community requests for funds to procure drugs and medical equipment or allow rehabilitation of health	

		national level.<br>- Focus group discussions<br>- Interviews<br><br>**Data analysis**<br>Content analysis and data triangulation				facilities, and took a long time to respond to such requests<br><br>Lack of possibility to use health facilities of members' choice<br><br>**Human resource planning and management**<br>District managers did not ensure supervision of health staff to support delivery of quality services. FGDs with villagers in both districts raised concerns about the improper provision of services by health workers, including corruption, pilferage of drugs, absenteeism during working hours and discrimination against CHF members<br><br>**Stakeholder involvement**<br>In Uganda, the introduction of the scheme policy at the central level with little input from district managers resulted in the managers perceiving the implementation process as imposed and rushed with little time to prepare<br><br>**Referral systems**<br>Lack of comprehensive services coupled with lack of referral systems was reported	

						by district and community respondent groups to contributed to low perceived quality of care and, consequently, sclow enrolment in the scheme	
**Author & year**<br>Zhang and Wang, 2008<br><br>**Funding**<br>Not reported<br><br>**Study design**<br>Quantitative	To examine whether adverse selection persisted in the subsequent enrollments of the subsidized, voluntary-based CHI scheme and whether adverse selection would be more or less severe over time.	**Sampling size and method**<br>1169 households with 4160 – multistage random sampling<br><br>**Time frame**<br>2002-2006<br><br>**Data collection**<br>Questionnaires administered through interviews<br><br>**Data analysis**<br>Descriptive analysis<br>Regression analysis	**Sample population**<br>Household members of the insurance scheme<br><br>**Setting**<br>Fengsan Township, Guizhou Province of China.	**Type:** Voluntary-based Community Health Insurance (CHI) scheme<br>**Content:** Not reported<br>**Population covered:** Everyone including farmers and poor people<br>**Enrolment rate:** 71%<br>**Unit of Enrollment:** Household<br>**Source of fund:** Beneficiary and government<br>**Premium:** Yes; 10–15 RMB (1.4–2.0 US dollars) with annual subsidy of 20 RMB<br>**Cost-sharing:** Yes - co-payment 50-60%	*Economic status*<br>People who are less wealthy were less likely to enroll in the scheme. People who live in the poor quality houses are less likely to participate in the scheme than those live in the brick houses.<br><br>*Migration*<br>Rural-to-urban migrant workers are less likely to be enrollees than those who stayed home<br><br>*Age*<br>The elderly, middle age, and preschool children are more likely to enroll in the scheme than those people with	**Health status**<br>People who were less health were more likely to enroll in the scheme. Adverse selection took place even when premiums were set forth.<br><br>**Cost-sharing**<br>High copayment rate<br><br>**Amount and timing of premium**<br>High premium rate	

				**Role of government:** Financing and governance<br>**Provider-payment method** Not reported	age between 16 and 35 years old<br><br>*Marital status*<br>Married and those who are divorced or widowed are more likely to join the scheme than those who are single<br><br>*Gender*<br>Females were more likely to enroll.		
**Author & year**<br>Gnawali et al. 2009<br><br>**Funding**<br>The German Research Foundation (DFG)<br><br>**Study design**<br>Quantitative	To quantify the impact of community-based health insurance (CBI) on utilization of health care services in rural Burkina Faso.	**Sampling size and method**<br>1309 households–step-wedge cluster randomization sampling<br><br>**Time frame**<br>May/June 2006<br><br>**Data collection**<br>Household survey<br><br>**Data analysis**<br>Descriptive analysis<br>Regression analysis	**Sample population**<br>Insured and uninsured members of the population<br><br>**Setting**<br>Burkina Faso	**Type:** Community-based health insurance (CBI)<br>**Content:** General and specialized consultation; Essential and generic drugs (if prescribed); Laboratory tests (also for antenatal care); Inpatient hospital stays (up to 15 days per episode of care); X-rays; Emergency surgery, Ambulance	*Ethnicity*<br>The Bwaba ethnic group was less likely to enroll in the scheme<br><br>*Education*<br>High education increased enrolment in the CBI.<br><br>*Per capita expenditure*<br>In the rich quartile, high per capita expenditure implied increased enrolment rates.	***Household Dynamics***<br>*Household size*<br>Larger households and households with younger heads were less likely to enroll in CBI<br><br>**Amount and timing of premium**<br>High premium rate (subsidize 50% of the premium rate for the very poor)<br><br>***Attitude Factors***<br>*Willingness to enroll*<br>Low enrolment rate (5–6%), which is much lower than expected enrolment rate of 50.37%. The expected enrolment rate was estimated	***Household Dynamics***<br>*Household size*<br>Households with a higher proportion of children under 5 were more likely to enroll in the scheme<br><br>***Attitude Factors***<br>*Consumer perception of scheme*<br>The overall perceived quality of care turned out to be significant; The use of curative care at least once in the previous 12 months appeared to be significant.

				transport (when authorized by provider population covered: all population excluding private providers such as traditional healers **Enrolment rate:** 5.2% **Unit of Enrollment:** Household **Source of fund:** Beneficiaries and government **Premium:** Yes - At the individual level - 1500 CFA per adult per annum and 500 CFA per child per annum in a household (1D = 655 CFA).Also each household has to pay 200 CFA as a membership fee. **Cost-sharing:** No **Role of government:** Governance and financing		using a willingness-to-pay (WTP) study. A subsequent study has pointed that the substantial gap between the actual and expected enrolment rate could be due to the weaknesses of the WTP technique, the difference between the WTP scenario used and the actual benefit package of the CBI, and marketing methods as well<br><br>**Accessibility of facility** Additional barriers to access, which are not directly addressed by CBI, are transport costs and opportunity costs of seeking care, and could be the custom of informal payments even if insured	

				**Provider-payment method** Capitation payment			
**Author & year** Haddad et al. 2011<br><br>**Funding** International Development Research Centre<br><br>**Study design** Case study	Design and implement a community- based health insurance (CBHI) scheme to reduce financial barriers to health care, especially among the poor, Strengthen local governance capacities in monitoring and promote a culture of evidence-based decision making, Develop an evidence base for appropriate interventions to improve the health of the most vulnerable Paniya tribe (a previously enslaved tribe) using appropriate ethical and methodological approaches	**Sampling size and method**<br>- 3352 Households/ 16110 individuals<br>- Tribal colonies (n=38)<br><br>**Time frame**<br><br>Phase I: 2002-2005<br>Phase II: 2006-2010<br><br>**Data collection** Survey<br>Focused group discussions<br>Review of previous reports<br><br>**Data analysis** Thematic analysis	**Sample population** Households, women, tribal colonies<br><br>**Setting** South Indian state of Kerala	**Type:** Community based health insurance (CBHI)<br>**Content:** Not reported<br>**Population covered:** Women, indigenous groups<br>**Enrolment rate:** Not reported<br>**Unit of Enrollment:** Not reported<br>**Source of fund:** Beneficiaries<br>**Premium:** Not reported<br>**Cost-sharing:** Not reported<br>**Role of government:** Governance (formerly)<br>**Provider-payment method** Not reported		**Amount and of premium criteria** Major inefficiencies in the structure and implementation of policies targeting the STs (certain indigenous groups (known as Scheduled Tribes) due mainly to the application of uniform policies to all STs, which are heterogeneous groups	**Consumer understanding of concept of health insurance** Women office bearers of SNEHA have gained a profound knowledge of health insurance and of inclusion issues<br><br>**Management/administrative structure** Women office bearers of SNEHA have shown leadership and have overcome political pressure (SNEHA at present is not ruled by its governing body but by informal leadership).

**Author & year**<br>Kiwara, 2007<br><br>**Funding**<br>NR<br><br>**Study design**<br><br>Quantitative and qualitative	To assess how group premiums can help poor people in the informal economy prepay for health care services(in an attempt to find a method which retains beneficiaries for a longer time with minimal attrition/dropout)	**Sampling size and method**<br>Purposive sampling of 4 groups with a total of 1416 individuals:<br>2 groups with a total of 714 prepaying through group premiums<br>2 groups with a total of 702 paying through individual premiums<br><br>**Time frame**<br>3 years<br><br>**Data collection**<br>Questionnaire and focus group discussions<br><br>**Data analysis**<br>Not reported	**Sample population**<br>Informal economy operators (cobblers, carpenters, welders and small scale market retailers)<br><br>**Setting**<br>Tanzania	**Type:** Mutual health scheme (Micro-health insurance schemes)<br>**Content:** Members receive all needed outpatient care, specified Laboratory tests and generic prescriptions. Dentures, artificial limbs and hearing aids are excluded.<br>**Population covered:** Everyone in the community<br>**Enrolment rate:** Not reported<br>**Unit of Enrollment:** Individual or group<br>**Source of fund:** Beneficiaries<br>**Premium:** Yes (1.3$)<br>**Cost-sharing:** Yes (0.5$)<br>**Role of government:** Support (Such as		**Amount and timing of premium**<br>Drop out in individual-based premiums is higher than that in group-based premiums.	**Amount and timing of premium**<br>Mutual cells were established to make it easy for beneficiaries to encourage each other to pay premiums or act as pressure groups for group leaders to pay premiums.<br>76% of the members from the two groups who chose group premium payment were still members of the prepayment health scheme and were receiving health care.<br><br>***Attitude Factors***<br>*Willingness to enroll*<br>The population is willing to register in a health insurance scheme studied in both groups indicated willingness to join the mutual on a prepayment basis, at a rate Tshs 1,500 (US $ 1.5) per month per a family of six and payment Tshs 500 (US $0.5) per episode].<br><br>**Consumer understanding of concept of health insurance**<br>Training workshops for beneficiaries to allow them to understand the schemes better

				tax exemption for the UMASIDA - Umoja wa Matibabu sekta Isiyo rasmi Dar-es-Salaam)<br>**Provider-payment method**<br>Not reported			
**Author & year**<br>Dong, 2009<br><br>**Funding**<br>This work was supported by the collaborative research grant 'SFB 544' of the German Research Society (DFG).<br><br>**Study design**<br>Quantitative study	To identify the reasons why previously enrolled people decide not to renew their membership in following years (to understand the specific reasons motivating people to drop-out of schemes)	**Sampling size and method (9)**<br>756 households from rural area and 553 households from Nouna; random two-stage cluster sampling<br><br>**Time frame**<br>May 2006<br><br>**Data collection**<br>Survey + data from the management unit of the CBI scheme (databank)<br><br>**Data analysis**<br>Mixed (descriptive and regression)	**Sample population**<br>Households<br><br>**Setting**<br>Nouna Health District, rural Burkina Faso	**Type:**<br>Community-based health insurance<br><br>**Content:**<br>Not reported<br><br>**Population covered:**<br>Households in the catchment area of the demographic surveillance system (DSS) of the Nouna Health District, rural Burkina Faso.<br>**Enrolment rate:**<br>5.2-6.3%<br>**Unit of Enrollment:**<br>Household<br>**Source of fund:**<br>Not reported<br>**Premium:**	*Education*<br>The household heads in the drop-out group had a significantly lower education than in the non-drop-out group<br><br>*Age*<br>Higher household head's age<br><br>*Religion*<br>The households in the drop-out group were more likely not to be Muslim<br><br>*Per capita expenditure*<br>Higher household expenditure	***Household Dynamics***<br>*Household size*<br>The households in the drop-out group also had a significantly higher household size<br><br>**Amount and timing of premium**<br>Main reasons of respondents to motivate their decision to discontinue membership in CBI were (%): Could afford no longer 28.4.<br><br>**Health status**<br>The following factors all had a positive effect on drop-out, meaning that they increased the probability that a household did not renew its membership in CBI<br>Lower number of illness episodes in the past 3 months (OR=0.87; p=0.76), fewer children or elderly in a household (OR=0.29; p=0.49)	

				NR<br>**Cost-sharing:**<br>NR<br>**Role of government:**<br>NR		***Attitude factor***<br>*Perception of scheme*<br>poor perceived quality of care had a positive effect on drop-out, meaning that they increased the probability that a household did not renew its membership in CBI<br><br>**Accessibility to facility**<br>*Geographical Coverage*<br>Positively influenced dropout: shorter distance to the contracted health facility	
**Author & year**<br>Alkenbrack,2013<br><br>**Funding**<br>World bank<br><br>**Study design**<br>Mixed (qualitative, quantitative and model)	To examine the relative with a particular interest in knowing whether health status and socioeconomic status are significant determinants of enrolment, to explore the likelihood that CBHI can be further expanded geographically to new districts by comparing characteristics of	**Sampling size and method (9)**<br>3000 households (2-stage random cluster sampling) using a case-comparison study design (enrolled/un-enrolled households; 1:2)<br><br>6 groups: 3: enrolled; 3: un-enrolled (55 participants)" purposive sampling	**Sample population**<br>Beneficiaries and un-enrolled households<br><br>**Setting**<br>Lao PDR	**Type:** Voluntary community-based health insurance<br>**Content:** outpatient and inpatient services and drugs purchased at hospitals<br>**Population covered:** Households who are self-employed or working in the informal sector and are not covered by other social health protection schemes	*Marital status*<br>Those enrolled were more likely to be married<br><br>*Education*<br>The probability of enrolment is significantly higher for those with vocational training or post-secondary education; than uninsured households<br><br>*Economic status*<br>Households that are better off financially are	**Amount and timing of premium**<br>The most frequent reason for never enrolling in CBHI was the inability to afford the premiums<br><br>**Facility-related factors**<br>Participants complained that CBHI members usually receive low quality drugs, while non-members are prescribed a variety of more expensive drugs.<br><br>**Attitude factors**<br>*Satisfaction of enrollee*<br>FGDs results contradict the quantitative findings, which indicated that CBHI members	**Consumer Awareness of Scheme**<br>CBHI members are also more likely than the uninsured to have attended a CBHI campaign<br><br>***Attitude Factors***<br>*Consumer perception of scheme*<br>CBHI households report a higher perception of quality of health care at the district hospital<br><br>*Trust in scheme*<br>CBHI members more likely to place higher trust in the scheme

	districts with and without CBHI. The objective is to use the findings from the household and district level to shed light on the prospects for expanding CBHI nationally.	**Time frame** February to April 2009<br><br>**Data collection** 1) Survey 2) Focus group discussion 3) District level secondary data, compared to characteristics of districts with and without insurance to assess the likelihood of scaling-up geographically, using univariate and multivariate (probit model analyses)<br><br>**Data analysis** Mixed (quantitative: descriptive and regression: probit model; qualitative: thematic analysis)		**Enrolment rate:** 2.2-2.3% **Unit of Enrollment:** Households **Source of fund:** Government, Donors and premiums **Premium:** Yes (2.5 to 3% of average household income of the country) **Cost-sharing:** Not reported **Role of government:** Governance	significantly more likely to enroll in CBHI	have higher perceptions of quality than non-CBHI members. It is therefore more likely that members who have positive experiences with CBHI maintain enrolment in CBHI, and less likely that perceptions of good quality of care at district hospitals are enticing households to enroll in the scheme<br><br>**Human Resource planning and Management**<br><br>Both members and non-members reported that health care staff members do not have the skills to diagnose health problems and that productivity is low<br><br>**Facility-related factors** Lack of equipment is a problem in the district hospitals.<br><br>**Health status** The majority of the respondents in the FGDs reported that enrolling in CBHI allows people to minimize their risk, some felt that enrolling in CBHI is a risky venture and that enrolment actually increases risk, because one can't be sure that benefits will	***Relative Relations*** CBHI members are more likely to have more close relatives and friends in the scheme<br><br>***District-level Factors*** Relative to non-CBHI districts, CBHI districts have a significantly higher population density, lower poverty rates, higher literacy rates, and a higher proportion of the population working in the non-agricultural sector, more likely to have electricity<br><br>**Accessibility of facility** *Geographical Coverage* Closer proximity to nearest health center. Residents of non-CBHI districts are located three times further from a health facility than CBHI districts<br><br>**Household Dynamics** *Household size* CBHI households are larger (5.3 vs. 4.7; p<0.001), The results show that CBHI households are larger than uninsured households.

						be delivered when they are needed.<br>Households in which a family member has either a chronic illness or had difficulty performing regular activities in the past three months were significantly more likely to enroll in CBHI than households with no signs of illness.	
