# Supplementary material for: Barriers and facilitators to implementation, uptake and sustainability of community-based health insurance schemes in low- and middle-income countries: a systematic review
Source: Int J Equity Health. 2018 Jan 29;17:13. doi: 10.1186/s12939-018-0721-4 (PMC5789675; doi:10.1186/s12939-018-0721-4)
Supplement: Supplementary file 5 — Quality appraisal of included studies (PDF 697 kb) [file 12939_2018_721_MOESM5_ESM.pdf]

Supplementary file 5: Quality appraisal of included studies

Table 1: Risk of bias table reflecting review authors' detailed judgments for quantitative data

Author's last name, publication year*	Developing and applying appropriate eligibility criteria	Measurement of exposure	Measurement of outcome	Controlling for confounding	Completeness of data
1. (Mladovsky, 2014)	Low risk<br><br>Sampling method and criteria explained: "Since overall population enrolment rates were low, disproportionate stratified sampling was used. In each case study, a list of households which had ever purchased a CBHI policy was used as a sampling frame for the random selection of members. "	Low risk<br><br>"The dependent variable is membership of CBHI.<br><br>"Because this study is concerned with the decision to ever enroll in CBH, both households with active and expired policies are referred to as "members" and are included in the analysis."	High risk<br><br>Self-reported household questionnaire was used	Low risk<br><br>Logistic regression model was conducted, controlling for potential confounding variables	Unclear risk<br><br>Authors did not comment on completeness of data
2. (Kamau and Njiru, 2014)	Low risk<br><br>Sampling method and criteria clearly explained	Unclear risk<br><br>It was not clear how insurance status was measured	High risk<br><br>"Data were collected using a structured questionnaire	High risk<br><br>Authors did not control for potential confounding variables	Unclear risk<br><br>Authors did not comment on

	"To identify the individual households to participate in the survey, the census listing of households was used as a sampling frame. The first household was identified randomly; thereafter systematic random sampling was used to identify the subsequent households until the desired sample size was achieved."		administered to randomly selected heads of households. The questionnaire was designed to capture data on the respondents' health seeking behaviour, experience with the scheme, their knowledge and perceptions about the scheme, health care financing and general health care experiences."		completeness of data
3. Dong et al., 2009)	Low risk<br><br>Clear eligibility criteria "756 households from the rural area and 553 households from the town of Nouna were randomly selected by using a two-stage cluster sampling procedure"	Low risk<br><br>"Information from CBI agency databank is used to describe the general situation of enrolment and drop-out"	High risk<br><br>"Household survey is used to collect information on the factors influencing dropping out from CBHI schemes"	Low risk<br><br>Multivariate analysis was conducted to account for potential confounders	Unclear<br><br>Authors did not comment on completeness of data

4.	(Alkenbrack et al., 2013)	Low risk<br><br>"Sample consisted of 3000 households, selected from 87 villages across 6 districts."<br><br>"A two-stage cluster sample was randomly selected: first, villages were selected with probability proportional to population; then households were randomly selected in one of two ways. CBHI member households were randomly selected from member lists in villages, while comparison households were randomly selected from the village registry."<br><br>The response rates for the CBHI and non-CBHI strata were 99.7% and 96.9%, respectively.	Low risk<br><br>CBHI households were eligible for the study if they had been enrolled for at least one year.	High risk<br><br>Household survey was used to collect information on variables	Low risk<br><br>Multivariate analysis was conducted using a probit model, which models the factors associated with roll-out of CBHI to the districts.	Low risk<br><br>"Response rates for the CBHI and non-CBHI strata were 99.7% and 96.9%, respectively."
5.	Bending et al 2011	Low risk<br><br>Clear sampling frame and eligibility criteria<br><br>Study site covered 30 villages in all 14 districts of all regions.	Unclear risk<br><br>It was not clear how insurance status was measured<br><br>"The choice of the household is related to	High risk<br><br>The data for this study comes from a household survey conducted in Sri Lanka in 2008.<br><br>"The survey	Low risk<br><br>Multivariate probit regressions were employed to analyze factors affecting the participation in different types of insurance	Unclear risk<br><br>Authors did not comment on completeness of data

	The survey sampling frame is a census of households randomly selected from the client bases of the five respective MFIs and allocated among the districts in which the MFIs are operating. From each district, two or three of the respective MFIs have been chosen except from Vavunia and Batticaloa, which are in the Northern and Eastern provinces, where only SEEDS is operating. The selected number of households from each district differs from 15 to 50 households. Two or three villages from each district and one Community Based Organization (CBO) from each village are randomly picked representing the selected MFIs from each district. A total of 30 CBOs	the decision to buy or not buy any insurance. Second, if the household decides to buy insurance, then the second choice is to buy which type of insurance."	questionnaire contained detailed sections on demographic and socioeconomic household characteristics, household assets, the occurrence of shocks, risk management strategies, evaluation of household's risk self-assessment and situation"		

	are included in the survey with 10 to 15 clients each selected randomly from the client base"				
6. Bhat, 2006	High risk<br><br>Insufficient data on sampling frame and eligibility criteria	Unclear risk<br><br>It was not clear how insurance status was measured<br><br>"The choice of the household is related to the decision to buy or not buy any insurance. Second, if the household decides to buy insurance, then the second choice is to buy which type of insurance."	High risk<br><br>"Data was collected through a questionnaire"	Unclear risk<br><br>Econometric analysis was used to find factors affecting health insurance purchase decisions	Unclear risk<br><br>Authors did not comment on completeness of data
7. Ito et al 2010	Low risk<br><br>209 households are randomly selected from 3 villages of rural Bangalore, Karnataka in September, 2008	Unclear risk<br><br>Authors did not mention how insurance status was determined	High risk<br><br>Take-up decision using household data<br><br>"The questionnaire consists of two parts, one on household background information and perceptions on insurance, and another on results from experiments"	Unclear risk<br><br>Prospect theory presumes that people behave in a risk averse way in evaluating gains but in a risk loving way in evaluating losses<br><br>"links prospect theory and hyperbolic discounting to household decision on purchasing insurance with using household survey data"	Unclear risk<br><br>Authors did not comment on completeness of data

8.	Lammers 2010	Low risk<br><br>Clear sampling frame and eligibility criteria;<br><br>"A representative sample of household members from small entrepreneurs in Lagos who recently gained access to a subsidized insurance program."<br><br>"Out of 59 markets, 16 markets were randomly selected stratified by area and selected with probability proportional to size."	Unclear risk<br><br>It was not clear how insurance status was measured	High risk<br><br>Self-reported household survey	Low risk<br><br>Logistic regression model were conducted to control for potential confounding variables	Unclear risk<br><br>Authors did not comment on completeness of data
9.	Polonsky, 2008	Low risk<br><br>Clear sampling frame and eligibility criteria; high response rate<br><br>"A random sample of 506 households in villages operating insurance schemes in rural Armenia<br><br>Sampling took place in nine villages randomly selected from a list of 36 villages operating an insurance scheme in Vayots Dzor district"<br><br>"Households were selected by random walk technique. A calculation based on the need to detect differences in	Low risk<br><br>Exposure was scheme membership status which was easy to identify<br><br>"Three comparable non-scheme villages (in terms of size, sources of income and geographical accessibility) were included as controls in the analysis, in order to correct for the advantages that the scheme introduces, both for the insured and uninsured, in villages operating it"	High risk<br><br>"household survey data collected in July 2001 on health status, service utilization and health care expenditure"	Low risk<br><br>Univariate and multivariate (Poisson and logistic regression) analyses were undertaken to identify the determinants of health facility utilization, and equity of access across socio-economic strata.	Low risk<br><br>All households consented to involvement in the study.

	payments between scheme members and non-members yielded a sample size requirement of 500 households.”				
10. Jütting2004	Low risk<br><br>Clear sampling method and eligibility criteria; response rate was high<br><br>“First, we selected 4 villages out of the 16 villages in which mutuals operate. Each of the selected villages has only one mutual. In a second step, we randomly selected households for the interviews. In all four villages, members and nonmembers were interviewed. To get a random sample from the four villages, we used household lists of all inhabitants of the four villages to calculate the percentage distribution	Unclear risk<br><br>“In each of these cases, the evaluation of a policy intervention or institutional innovation involves the problem of assigning individuals randomly to non-program control groups and others to program treatment groups. Thus the identification of an adequate control group is the first, and even the most important, step in trying to control for self-selection.”	High risk<br><br>A household survey was carried out. The survey began with a pretest in March 2000;	Low risk<br><br>Regression models were used to examine correlations between social capital and other study variables.<br><br>“The main potential confounders that are commonly included in quantitative studies on CBHI enrolment and on social capital and health are included in this study.”	Low risk<br><br>Participation rate was over 95%

	between members and nonmembers and their respective weight in the sample. We interviewed 346 households, 70% members and 30 % nonmembers. The data set contains information on roughly 2,900 persons, 60 percent members and 40 percent nonmembers."				
11. Schneider 2004	Low risk<br><br>Clear eligibility criteria and sampling frame<br>"The household survey includes 2,518 households that were successfully interviewed in the three pilot districts. The sample was based on the same sampling frame as the Rwandan Demographic and Health	Unclear risk<br><br>The household questionnaire collected information on households' participation in CBHI.<br>"For the analysis, the sample population is divided into two groups: CBHI members in pilot districts, and CBHI	Low risk<br><br>The prepayment household survey used three structured questionnaires for data collection: a socioeconomic household questionnaire, a curative questionnaire, and a	Low risk<br><br>"A logit regression model is used to determine households' CBHI enrollment probability and the extent to which this decision is influenced by specific sociodemographic and economic characteristics"	Unclear risk<br><br>Authors did not comment on the completeness of the data

	Survey (DHS) 2000, covering 11 health regions in Rwanda. Households were sampled at random from a list of primary households from sample cells identified in the national DHS sample, rendering the household survey sample representative to the district level.”	nonmembers in pilot districts.”	preventive care questionnaire. “The impact of prepayment schemes on insurance and providers’ utilization, cost, and finances has been analyzed from monthly routine data collected from providers and health insurance schemes over a two-year period in the three districts”		
12. Ranson 2004	Low risk<br><br>Sampling method and criteria explained.	Unclear risk<br><br>Age-matched insured and uninsured women	High risk<br><br>Household questionnaire was used to collect	Low risk<br><br>“A number of individual-level, demand-side variables as well as	Unclear risk<br><br>Authors mentioned missing variables

	"Two-stage, random cluster sampling was used. The primary sampling units (PSUs) were villages. Twenty villages were selected randomly (using random-number tables); the probability of selection was equal for all villages regardless of size. The secondary sampling units were households. Within each village, the insured were randomly selected from lists compiled by SEWA, and the uninsured were randomly selected from census or voting lists. In 10 villages, 14 SEWA households and 14 uninsured households were sampled, and in 10 villages, 14 SEWA households and 28 uninsured households were sampled; therefore, 700 households	were compared using survey data	information on the different variables	characteristics for hospitalizations were controlled for."	that need to be addressed, including presence of chronic illnesses, and insufficient controlling for wealth.

	are included in this analysis)."				
13. Gumber 2004	Unclear risk<br><br>Eligibility criteria was clear but sampling was purposive with no prior house listing<br><br>1,200 households from rural and urban areas. The households were stratified into four categories according to health insurance status.<br>The survey was conducted in eight slum-dominated localities in the city of Ahmedabad and six neighboring villages. On average, 60 households per village and 90 households per urban locality were selected. The criterion for selecting a village or an urban locality was that the settlement should have a	Unclear risk<br><br>It was not clear how health insurance status was determined	High risk<br><br>Household-level data from the pilot study was used to examine determinants of enrollment in the community-based financing scheme	Low risk<br><br>"A multinomial logit model is used to identify various determinants of being enrolled in the SEWA health insurance plan among members of SEWA."<br><br>These variables include income, gender, age, marker on chronic illness, and disability. β is a vector of coefficient estimates and ε is the error term. "	Unclear<br><br>Authors did not comment on completeness of data

	cluster of households covered by the SEWA and ESIS plans.. The sample canvassed from each settlement included about equal numbers of households from the ESIS, SEWA, and uninsured categories (20 each from a village and 30 each from an urban locality). The sample was purposive, and no house listing prior to the survey was carried out.”				
14. Supakankunti 2004	High risk<br><br>Sampling method and criteria not explained clearly. “The target population was identified by the research team and the provincial health office. The provincial and district health officers and the research team went to the six districts to explain the program to the communities and to	Low risk<br><br>Secondary data was used to determine providers, and the number of insured and uninsured in the province before and after the implementation of the program	Low risk<br><br>Authors used a mix of primary data (questionnaire) and secondary data (statistics) to collect information on different variables<br><br>-Cost data obtained from the health center	Low risk<br><br>“Logistic regression model was then used to identify significant predictors of health card purchase and non-purchase patterns as well as the continuation of card purchase.”	Unclear risk<br><br>Authors did not comment on completeness of data

	investigate the readiness of the communities. A sample of 1,000 households from the target population were selected by health officers”<br><br>“There are four groups of households in the sample:<br>(1) individuals who had not purchased a health card during the period 1993–1995, or card non-purchase;<br>(2) individuals who had purchased a health card for the first time in 1995, or new card purchase;<br>(3) individuals who had repurchased a health card, or continued card purchase; and<br>(4) individuals who had not repurchased a health card, or health card dropouts.”		and community hospital		
15. (Noubiap et al., 2013)	High risk	Unclear risk	Unclear risk	High risk	Unclear risk

	Sampling of the participants was according to convenience, there is a high possibility of selection biases.	Non-random sampling of the participants may leave out some of the exposures relevant to the research question.	Questionnaire was pretested but not validated.	The questionnaire only tested the knowledge of the respondents that were aware of the CBHI and failed to test the reasons for those who were unaware of the CBHI.	There is imprecision in the information due to the convenient sampling.
16. (Onwujekwe et al., 2009)	Low risk<br><br>Eligibility criteria and sampling method was clearly stated. Response rate was high<br><br>971 respondents in two communities selected by simple random sampling. The participants were selected by simple random	Low risk<br><br>"One successful site and one non-successful site were purposively chosen. The level of CBHI scheme success was determined by examining enrolment data in the scheme as well as views of the state	High risk<br><br>Data was collected using a questionnaire that was administered to 971 respondents in two communities selected by simple random sampling<br><br>Study was based reported utilization of services and not utilization based on facility records was assessed	Unclear risk<br><br>Authors did not report effort to control for potential confounding variables	Low risk<br><br>Few missing data Total of 455 and 516 completed questionnaires were available for analysis.

	sampling from a sample frame of PHC house numbering system.<br>Response rate was 88%.	ministry of health program officers”			
17. (Nsiah-Boateng and Aikins, 2013)	Low risk.<br>Study population consisted of membership data of Ga DMHIS and selected heads of surveyed households<br>“A multistage sampling method was used to select the study subjects. In all, 376 household heads were sampled on the basis of an estimated prevalence rate of 43% membership coverage, a confidence level of 95%, and 5% margin of error.”	Low risk<br>“The registration files were reviewed in terms of the number of people registered, number of membership cards issued, and number of renewals for each year under review”	Low risk<br>Data was obtained from both documentation review and surveys<br><br>“Documents on membership, operational reports, audited reports, financial statements, and claims payment books of the scheme were reviewed”	Unclear risk<br>Confounding variables were not reported.	Unclear risk<br>365 out of 367<br>Of 376 sampled household heads, 365 participated in the survey

			"A community household survey was conducted in the Madina Township to determine the community coverage rate"		
18. (Gnawali et al., 2009)	Low risk<br><br>Clear eligibility criteria and sampling frame<br>"All the households registered in the DSS were used as a sampling frame for the household survey. The sample size calculation was based on the assumptions that there would be 90% power to detect a difference	Unclear risk<br><br>It was not clear how insurance status was measured	High risk<br><br>Outcome measured via self-administered questionnaire	Low risk<br><br>Authors conducted logistic model for enrolment decisions	Low risk<br><br>Baseline measurements were taken prior to the study then the results were compared.

	between insured and uninsured households of one visit to the health services per year and that enrolment rate would be at least 50%. This resulted in a sample of 378 households (189 per group). In order to allow for intra-cluster correlation due to cluster randomisation, a design factor of 2.16 was applied. Thus, the minimum sample size agreed was 990 households distributed across 33 clusters."				
19. (Zhang and Wang, 2008)	Low risk	Low risk<br><br>"Data from the 2004, 2005 and 2006 surveys	Unclear risk<br><br>"Data from 2002, 2004 and 2005	Low risk<br><br>"A random effect Linear Probability Model (LPM) is	Low risk<br><br>Dropout rates were weighted

	Sampling method well explained with random sampling taking place.<br>"The sample population was chosen through a multistage sampling approach"<br><br>Follow up rate was 83% with the characteristics of non-followed up individuals identified.	were used to obtain the enrollment choice of the farmers."	surveys were used to obtain lagged value of health status variables and SES variables which would have influences on the enrollment choice of the next year."	used to test whether adverse selection persisted over the three waves of the CHI scheme and whether there was significant difference in the extent of adverse selection over time, controlling for unobserved individual level heterogeneity."	for their possibility in affecting the outcome measured, whereby results showed that it does not affect the outcome.
20. (Wang et al., 2006)	Low risk<br><br>Sampling method an frame clearly explained<br>"The study population was chosen through a multistage sampling process. In the first stage, random sampling was	Low risk<br><br>"In the context of our study, all rural residents in the study area can be categorized into two groups based on their RMHC enrollment	Unclear risk<br><br>While the study relied on longitudinal data set, independent variables were measured using follow-up surveys	Low risk<br><br>Three logistic regression analyses were conducted	Low risk<br><br>Pre and posttest of the same sampling design was used with an 83% follow up rate.

	used to select 6 villages in the study area. In a second stage, all households with family members in the high-risk population, as well as about one out of three other households chosen at random, were selected. The entire sample includes 1173 households with 4160 residents from 6 sampled villages."<br><br>Follow up rate was 83% with the characteristics of non-followed up individuals identified.	status: enrolled in and non-enrolled"			
21. (Supakankunti, 2000)	High risk	Low risk	Low risk	Low risk	Unclear risk

	No clear eligibility criteria; Sampling frame and method not explained<br>"The target population was identified by the research team and the provincial health office. The provincial and district health officers and research team visited the six districts to explain the program to the communities and also to investigate the communities' readiness. A sample of 1000 households from the target population was selected by health officers."	Different sources of primary and secondary data were used to collect information<br><br>Surveying method was valid.	Different sources of primary and secondary data were used to collect information<br><br>Surveying method was valid.	The logistic regression model was then used to identify significant predictors of health card purchase and non-purchase as well as the continuation of card purchase.	Although it was initially stated that a sample of 1000 households from the target population was selected by health officers, the total number of response households was 1005."
22. Rao et al. (2009)	Low risk	Low risk	Low risk	High risk	Low risk

	Eligibility criteria and sampling method well explained.<br>“Between one and two villages were randomly sampled from villages having 100 or more households and within 90 minutes walking distance of the pilot facility. Each village was divided into four segments and one segment was randomly chosen. At the central point of the chosen segment one direction was randomly selected. All households lying in the selected direction were	“Health facilities implementing the CHF pilots submitted various monthly reports on the performance of the pilots.”	Data for this study were taken from three sources: reports from routine project monitoring, the health management information system (HMIS), and household surveys of facility catchment areas.<br><br>Surveying method was valid.	Controlling for potential confounding variables were not reported	Pre and post-test of the same sampling design was used.

	numbered and a starting point randomly picked." This yielded a total of 320 households				
23. (Hao et al., 2010)	Low risk<br><br>Stratified cluster sampling method to select poor families who have been enrolled in MFA scheme in rural areas of ChongQing. All family members of the enrolled households were interviewed.<br>748 and 1129 respondents from two kinds of project towns participated in the survey. Among them, 625 and 869 respondents were	Unclear risk<br><br>Subjective measurement of exposure using self-administered surveys<br><br>"Independent variables were selected based on Anderson Behaviour Model of health service unitization [20-22]. This model has been extensively employed to explain health care access and utilization"	High risk<br><br>Subjective measurement of outcomes using self-administered survey	Low risk<br><br>Two-level linear multilevel model and binomial regressions with a log link were used to assess influencing factors on different response variables measuring service utilization.	Low risk<br><br>This survey response rate was 94%.

	included (age≥15) in the analysis of this study.				
24. (Parmar et al., 2012)	Low risk<br><br>Clear eligibility criteria and sampling frame<br>"The study area, covering 41 villages and 1 town, was divided into 33 clusters and CBHI was randomly offered to these clusters. 990 households i.e. 30 households per cluster were randomly included in the the Nouna Health District Household Survey (NHDHS), approximately 7900 individuals or 10% of the population.	Unclear risk<br><br>The analysis included only those individuals who were offered CBHI in a particular year<br>"We created a binary choice dependent variable that depicted the insurance status of the individual for every year (1 = individual enrolled in the scheme; 0 = individual not enrolled in the scheme)."	Unclear risk<br><br>The data was collected by a household panel survey 2004–2007 from randomly selected households in these 33 clusters (n = 6795).<br><br>"Every year, the NHDHS field team interviews the household members of these 990 households and	Unclear risk<br><br>"To study adverse selection, we wanted to estimate the influence of health status on insurance status, after controlling for all other variables. A fixed effects (FE) linear probability model, that took advantage of the panel nature of the sample i.e. repeated observations, was used. A linear probability model was preferred as it can be used to estimate fixed effects without losing	Unclear risk<br><br>"The random sample originally consisted of 990 households comprising of approximately 7900 individuals. Our study was based on 6713 individuals and all these individuals were not present all years."

	The DSS provided the sampling frame”		collects data on demographic and socio-economic indicators, self-reported morbidity, health care seeking behavior, insurance membership, and perceptions about the quality of health services”	a lot of sample, as would be the case with a fixed effects logit model.”<br><br>Small sample could have biased the regression results.”	
25. Mladovsky, 2014 (social)	Low risk<br><br>Sampling method and criteria explained:<br>“Since overall population enrolment rates were low, disproportionate stratified sampling was used. In each case study, a list of households which had ever purchased a CBHI policy	Low risk<br><br>“The dependent variable is membership of CBHI. There is no reason to believe there is bias in enrolment status.<br>“Because this study is concerned with the decision to ever enroll in CBH, both households	High risk<br><br>Self-reported household questionnaire was used to collect information on independent variables	Low risk<br><br>Logistic regression model was conducted, controlling for potential confounding variables	Unclear risk<br><br>Authors did not comment on completeness of data

	was used as a sampling frame for the random selection of members. "	with active and expired policies are referred to as "members" and are included in the analysis."			
26. Ozawa 2009	Low risk<br><br>"Cluster random household survey with a 28-cluster, 20-person per cluster sample (n=560). Stratified sampling on insurance status (n=360) was combined with population –proportional-to-size sampling (n=200) to ensure both statistical power and generalizability of findings"	Unclear risk<br><br>It was unclear how the insurance status was measured.<br>Four insurance status "renew", "new", "drop-outs" and never"<br><br>"Household who have had CBHI for more than one year and were enrolled at a time of the survey were classified as "renew" whereas household who joined CBI schemes for the first time in the past 12 months were classified as "new". Household who used to have	Low risk<br><br>The trust scale used to measure the outcome of interest had good construct validity and reliability	Low risk<br><br>Multinomial logistic regression models were used to control for potential confounding variables	Unclear risk<br><br>25 individuals were dropped out from analysis

		insurance but were not enrolled at the time of the survey were classified as "Drop-outs" whereas those who had never had CBHI were group as "never"			
27. Ouimet 2007	Low risk<br><br>Clear sampling frame and eligibility criteria<br>"All regions of Senegal having CBHI. A random sample of 394 subscribers was selected from 46 community CBHIs"	High risk<br><br>"Absence of comparison to an external group of non-subscribers"	High risk<br><br>"A survey was used to collect information about experience with the organization, and questions about six hypothetical situations to which one had to answer "fair" or "unfair"	Unclear risk<br><br>"Multilevel logistical analysis was conducted of the links between characteristics of subscribers and organizations and composite indicators representing values"<br><br>"Despite this, we were unable to identify CBHI level predictors. This may	Unclear risk<br><br>Author did not comment on completeness of data

				have been caused by the conjunction of small sample size and limited variance in predictors belonging to CBHI level”	
28. Ranson 2006	Low risk<br><br>Clear sampling frame<br>“All claimants in the 8 pilot sub-districts who were discharged from hospital during a 9-month period (1 April-31 December 2006).”	Low risk<br><br>“We also examined how the proportion of claimants using the 16 hospitals selected for PPS changed between 2003 and 2005. Since there are no comparable survey data for 2003, we extracted subdistrict specific data from Vimo SEWA’s computerized claims database.”	Low risk<br><br>Data on the uptake and socioeconomic status of users of the PPS system have been collected from a household survey	High risk<br><br>Authors did not control for potential confounding variables	Unclear risk<br><br>Author did not comment on completeness of data
29. Cofie 2013	Low risk<br><br>Clear sampling frame and eligibility criteria	High risk<br><br>“The survey assessed household heads or their	High risk	Low risk<br><br>“Bivariate analysis and multivariate logistic	Unclear risk<br><br>Author did not comment on

	“A survey was conducted with 250 randomly selected household heads<br>The HDSS database provided the sampling frame of 3,125 households from 15 communities. A systematic random sampling method was used to select a representative household sample from the 15 communities. The sample size was based on Cochran's formula for categorical data: with (α)= 0.05, thus (95% confidence level), margin of error (d) = 10%.”<br>96% response rate.	representatives’ exposure to the campaign, and its relationship to knowledge and enrolment.”	Subjective measurement of outcome using survey<br>“The survey assessed household heads or their representatives’ exposure to the campaign, and its relationship to knowledge and enrolment”	regression models were used to assess the association between household exposure to campaign and acquisition of knowledge as well as household exposure to campaign and enrolment.”	completeness of data

30. Mulupi 2013	Low risk<br><br>Clear sampling frame and eligibility criteria<br>"Survey households were selected through two stages. First two districts were selected from a list of districts with high CBHI coverage, following discussions with the KCBHFA. A list of villages where CBHIs operate was made, and 3 villages (clusters) were selected per district. All households in the selected villages were mapped and given a unique identification number. A	Unclear risk<br><br>"Data were collected on self-reported illness, health care utilization patterns, health care payments, knowledge of health insurance in general, the NHIF and preferred designs for a future NHIS using questionnaires"	High risk<br><br>". Data were collected on self-reported illness, health care utilization patterns, health care payments, knowledge of health insurance in general, the NHIF and preferred designs for a future NHIS using questionnaires"	High risk<br><br>Authors did not report controlling for confounding variables<br>"Study was conducted in two settings with a strong presence of CBHIs.. It is possible that this exposure contributed significantly to their perceptions on health insurance and that these are likely to be different in other settings"	Unclear risk<br><br>Authors did not comment on completeness of data

	total of 100 households per village were then randomly selected from a complete list of households. All selected households participated in the survey regardless of whether they belonged to a health insurance scheme or not."				

*Of the 31 studies reporting quantitative data, 23 were quantitative studies and 8 were mixed methods studies

Table 2: Methodological quality of studies reporting qualitative data

Author's last name, publication year (SN)*	Was there a clear statement of the aims of the research?	Is a qualitative methodology appropriate?	Was the research design appropriate to address the aims of the research?	Was the recruitment strategy appropriate to the aims of the research?	Was the data collected in a way that addressed the research issue?	Has the relationship between researcher and participants been adequately considered?	Have ethical issues been taken into consideration?	Was the data analysis sufficiently rigorous?	Is there a clear statement of findings?	How valuable is the research?
1. (Ranson et al., 2006)	Y	Y	Y	Y	Y	N	?	Y	?	Y
2. (Mladovsky et al., 2014)	Y	Y	Y	Y	Y	N	Y	Y	Y	Y
3. Mulupi et al, 2013	Y	Y	Y	Y	Y	N	Y	Y	Y	Y
4. Jutting 2004	Y	Y	Y	?	Y	N	N	N	Y	Y
5. Schneider (2005)	Y	Y	Y	?	Y	?	?	Y	Y	Y
6. (Basaza et al., 2010)	Y	Y	Y	Y	Y	N	Y	Y	Y	Y
7. (Basaza et al., 2008)	Y	Y	Y	Y	Y	N	N	?	Y	Y
8. (Ouimet et al., 2007)	Y	Y	Y	Y	Y	N	Y	Y	Y	Y
9. (Criel and Waelkens, 2003)	Y	Y	Y	Y	Y	N	N	Y	Y	Y
10. (De Allegri et al., 2006)	Y	Y	Y	Y	Y	N	N	Y	Y	Y

11. (Derriennic et al., 2005)	Y	Y	Y	Y	Y	N	N	Y	Y	Y
12. Kyomugisha et al 2009	Y	Y	Y	Y	Y	N	Y	Y	Y	Y
13. Kamuzora and Glison, 2007	Y	Y	Y	Y	Y	N	N	Y	Y	Y
14. Cofie 2013	y	y	y	y	y	N	N	?	Y	Y
15. Alkenbrack	Y	Y	Y	Y	Y	N	Y	N	?	Y
16. Ozawa	Y	Y	Y	Y	Y	N	Y	Y	Y	Y

*Of the 16 studies reporting qualitative data, 8 were qualitative studies and 8 were mixed methods studies

Table 3: Methodological quality of mixed methods studies (that did not differentiate between qualitative and quantitative data)

Study ID	Theoretical framework / literature review described?	Aims, objective, research questions clearly described	Context clearly described	Sample and recruitment described	Sample appropriate to research question	Method of data collection and analysis is clearly described	Method of data collection and analysis appropriate to research question	Attempts made to establish reliability or validity of data analysis	Are data, interpretations and conclusions clearly integrated	Pilot work conducted and described	Participation respondents (process/consent)	Useful contribution
1. (Basaza et al., 2007)	Y	Y	Y	Y	Y	Y	Y	Y	Y	N	Y	Y
2. (Shaw, 2002)	Y	Y	Y	N	?	N	N	?	Y	?	?	
3. (Kiwara, 2007)	Y	Y	Y	Y	Y	N	?	?	y	N	?	
4. (Robyn et al., 2014)	Y	Y	Y	Y	Y	Y	Y	?	Y	?	N	Y
5. (Rao et al., 2012)	N	Y	Y	Y	Y	Y	Y	?	Y	N	N	Y
6. (Uzochukwu et al., 2009)	N	Y	Y	Y	Y	Y	Y	Y	Y	N	N	Y

Table 4: Risk of bias in the included randomized controlled trial

Study name	Sequence generation	Allocation concealment	Blinding (participants, data collectors, outcome adjudicators)	Completeness of outcome data	Completeness of outcome reporting
Panda, 2014 (Panda et al., 2014)	Unclear risk<br>Not reported	Unclear risk<br>Not reported	Unclear risk<br>Not reported	Unclear risk<br>Not reported	Unclear risk<br>Not reported

References:

BASAZA, R., CRIEL, B. & VAN DER STUYFT, P. 2008. Community health insurance in Uganda: why does enrolment remain low? A view from beneath. *Health Policy,* 87**,** 172-84.

BASAZA, R. K., CRIEL, B. & VAN DER STUYFT, P. 2010. Community health insurance amidst abolition of user fees in Uganda: the view from policy makers and health service managers. *BMC Health Serv Res,* 10**,** 33.

CRIEL, B. & WAELKENS, M. P. 2003. Declining subscriptions to the Maliando Mutual Health Organisation in Guinea-Conakry (West Africa): what is going wrong? *Soc Sci Med,* 57**,** 1205-19.

DE ALLEGRI, M., SANON, M., BRIDGES, J. & SAUERBORN, R. 2006. Understanding consumers' preferences and decision to enrol in community-based health insurance in rural West Africa. *Health Policy,* 76**,** 58-71.

MLADOVSKY, P., SOORS, W., NDIAYE, P., NDIAYE, A. & CRIEL, B. 2014. Can social capital help explain enrolment (or lack thereof) in community-based health insurance? Results of an exploratory mixed methods study from Senegal. *Soc Sci Med,* 101**,** 18-27.

OUIMET, M.-J., FOURNIER, P., DIOP, I. & HADDAD, S. 2007. Solidarity or financial sustainability: an analysis of the values of community-based health insurance subscribers and promoters in Senegal. *Canadian Journal of Public Health/Revue Canadienne de Sante'e Publique***,** 341-346.

RANSON, M. K., SINHA, T., GANDHI, F., JAYSWAL, R. & MILLS, A. J. 2006. Helping members of a community-based health insurance scheme access quality inpatient care through development of a preferred provider system in rural Gujarat. *Natl Med J India,* 19**,** 274-82.

BASAZA, R., CRIEL, B. & VAN DER STUYFT, P. 2008. Community health insurance in Uganda: why does enrolment remain low? A view from beneath. *Health Policy,* 87**,** 172-84.

BASAZA, R. K., CRIEL, B. & VAN DER STUYFT, P. 2010. Community health insurance amidst abolition of user fees in Uganda: the view from policy makers and health service managers. *BMC Health Serv Res,* 10**,** 33.

CRIEL, B. & WAELKENS, M. P. 2003. Declining subscriptions to the Maliando Mutual Health Organisation in Guinea-Conakry (West Africa): what is going wrong? *Soc Sci Med,* 57**,** 1205-19.

DE ALLEGRI, M., SANON, M., BRIDGES, J. & SAUERBORN, R. 2006. Understanding consumers' preferences and decision to enrol in community-based health insurance in rural West Africa. *Health Policy,* 76**,** 58-71.

MLADOVSKY, P., SOORS, W., NDIAYE, P., NDIAYE, A. & CRIEL, B. 2014. Can social capital help explain enrolment (or lack thereof) in community-based health insurance? Results of an exploratory mixed methods study from Senegal. *Soc Sci Med,* 101**,** 18-27.

OUIMET, M.-J., FOURNIER, P., DIOP, I. & HADDAD, S. 2007. Solidarity or financial sustainability: an analysis of the values of community-based health insurance subscribers and promoters in Senegal. *Canadian Journal of Public Health/Revue Canadienne de Sante'e Publique***,** 341-346.

RANSON, M. K., SINHA, T., GANDHI, F., JAYSWAL, R. & MILLS, A. J. 2006. Helping members of a community-based health insurance scheme access quality inpatient care through development of a preferred provider system in rural Gujarat. *Natl Med J India,* 19**,** 274-82.

ALKENBRACK, S., JACOBS, B. & LINDELOW, M. 2013. Achieving universal health coverage through voluntary insurance: what can we learn from the experience of Lao PDR? *BMC Health Serv Res,* 13**,** 521.

BASAZA, R., CRIEL, B. & VAN DER STUYFT, P. 2007. Low enrollment in Ugandan Community Health Insurance schemes: underlying causes and policy implications. *BMC Health Serv Res,* 7**,** 105.

BASAZA, R., CRIEL, B. & VAN DER STUYFT, P. 2008. Community health insurance in Uganda: why does enrolment remain low? A view from beneath. *Health Policy,* 87**,** 172-84.

BASAZA, R. K., CRIEL, B. & VAN DER STUYFT, P. 2010. Community health insurance amidst abolition of user fees in Uganda: the view from policy makers and health service managers. *BMC Health Serv Res,* 10**,** 33.

CRIEL, B. & WAELKENS, M. P. 2003. Declining subscriptions to the Maliando Mutual Health Organisation in Guinea-Conakry (West Africa): what is going wrong? *Soc Sci Med,* 57**,** 1205-19.

DE ALLEGRI, M., SANON, M., BRIDGES, J. & SAUERBORN, R. 2006. Understanding consumers' preferences and decision to enrol in community-based health insurance in rural West Africa. *Health Policy,* 76**,** 58-71.

DERRIENNIC, Y., WOLF, K. & KIWANUKA-MUKIIBI, P. 2005. An assessment of community-based health financing activities in Uganda.

GNAWALI, D. P., POKHREL, S., SIE, A., SANON, M., DE ALLEGRI, M., SOUARES, A., DONG, H. & SAUERBORN, R. 2009. The effect of community-based health insurance on the utilization of modern health care services: evidence from Burkina Faso. *Health Policy,* 90**,** 214-22.

HAO, Y., WU, Q., ZHANG, Z., GAO, L., NING, N., JIAO, M. & ZAKUS, D. 2010. The impact of different benefit packages of Medical Financial Assistance Scheme on health service utilization of poor population in Rural China. *BMC health services research,* 10**,** 170.

KAMAU, N. & NJIRU, H. 2014. Community based health insurance schemes: lessons from rural Kenya. *J Health Care Poor Underserved,* 25**,** 192-203.

KIWARA, A. D. 2007. Group premiums in micro health insurance experiences from Tanzania. *East Afr J Public Health,* 4**,** 28-32.

MLADOVSKY, P. 2014. Why do people drop out of community-based health insurance? Findings from an exploratory household survey in Senegal. *Soc Sci Med,* 107**,** 78-88.

MLADOVSKY, P., SOORS, W., NDIAYE, P., NDIAYE, A. & CRIEL, B. 2014. Can social capital help explain enrolment (or lack thereof) in community-based health insurance? Results of an exploratory mixed methods study from Senegal. *Soc Sci Med,* 101**,** 18-27.

NOUBIAP, J. J., JOKO, W. Y., OBAMA, J. M. & BIGNA, J. J. 2013. Community-based health insurance knowledge, concern, preferences, and financial planning for health care among informal sector workers in a health district of Douala, Cameroon. *Pan Afr Med J,* 16**,** 17.

NSIAH-BOATENG, E. & AIKINS, M. 2013. Performance Assessment of Ga District Mutual Health Insurance Scheme, Greater Accra Region, Ghana. *Value in Health Regional Issues,* 2**,** 300-305.

ONWUJEKWE, O., ONOKA, C., UZOCHUKWU, B., OKOLI, C., OBIKEZE, E. & EZE, S. 2009. Is community-based health insurance an equitable strategy for paying for healthcare? Experiences from southeast Nigeria. *Health Policy,* 92**,** 96-102.

OUIMET, M.-J., FOURNIER, P., DIOP, I. & HADDAD, S. 2007. Solidarity or financial sustainability: an analysis of the values of community-based health insurance subscribers and promoters in Senegal. *Canadian Journal of Public Health/Revue Canadienne de Sante'e Publique***,** 341-346.

PANDA, P., CHAKRABORTY, A., DROR, D. M. & BEDI, A. S. 2014. Enrolment in community-based health insurance schemes in rural Bihar and Uttar Pradesh, India. *Health Policy Plan,* 29**,** 960-74.

PARMAR, D., SOUARES, A., DE ALLEGRI, M., SAVADOGO, G. & SAUERBORN, R. 2012. Adverse selection in a community-based health insurance scheme in rural Africa: implications for introducing targeted subsidies. *BMC health services research,* 12**,** 181.

RANSON, M. K., SINHA, T., GANDHI, F., JAYSWAL, R. & MILLS, A. J. 2006. Helping members of a community-based health insurance scheme access quality inpatient care through development of a preferred provider system in rural Gujarat. *Natl Med J India,* 19**,** 274-82.

RAO, M., KADAM, S., SATHYANARAYANA, T., SHIDHAYE, R., SHUKLA, R., RAMACHANDRA, S. S., BANDYOPADHYAY, S., CHANDRAN, A., ANITHA, C., SITAMMA, M., GEORGE, M. S., SINGH, V., SIVASANKARAN, S. & SHATRUGNA, V. 2012. A rapid evaluation of the Rajiv Aarogyasri community health insurance scheme in Andhra Pradesh, India. *BMC Proceedings,* 6**,** O4.

ROBYN, P. J., BARNIGHAUSEN, T., SOUARES, A., TRAORE, A., BICABA, B., SIE, A. & SAUERBORN, R. 2014. Provider payment methods and health worker motivation in community-based health insurance: a mixed-methods study. *Soc Sci Med,* 108**,** 223-36.

SHAW, R. P. 2002. Tanzania's community health fund: prepayment as an alternative to user fees.

SURAKANKUNTI, S. 2000. Future prospects of voluntary health insurance in Thailand. *Health Policy Plan,* 15**,** 85-94.
