# Supplementary material for: Barriers and facilitators to implementation, uptake and sustainability of community-based health insurance schemes in low- and middle-income countries: a systematic review
Source: Int J Equity Health. 2018 Jan 29;17:13. doi: 10.1186/s12939-018-0721-4 (PMC5789675; doi:10.1186/s12939-018-0721-4)
Supplement: Supplementary file 6 — Detailed findings of studies at the individual, interpersonal, community and systems level (PDF 635 kb) [file 12939_2018_721_MOESM6_ESM.pdf]

Supplementary file 6: Detailed findings of studies at the individual, interpersonal, community and systems level

1-Individual level

Individual level	
**Consumer awareness and understanding**	
Consumer awareness of scheme	**Total N= 6 studies (3 cross-sectional studies; 3 mixed studies)**<br><br><u>Barriers (n=2)</u><br>- In Afghanistan, a major reason why many households did not enroll was a lack of awareness about the CBHI program (K. D. Rao et al., 2009).<br>- In India, lack of awareness about the scheme as well as its location negatively affected the use of the scheme designed for rural members of the scheme (Ranson, Sinha, Gandhi, Jayswal, & Mills, 2006).<br><br><u>Facilitators (n=4)</u><br>- In Lao PDR, CBHI members were more likely than uninsured to have attended a CBHI campaign (<0.001) (Alkenbrack, Jacobs, & Lindelow, 2013).<br>- In Burkina Faso, the relationship between awareness about scheme and enrolment was observed to be statistically significant (p<0.001) (Cofie, De Allegri, Kouyate, & Sauerborn, 2013a)<br>- In Cameroon, awareness of CBHI schemes was significantly associated with a high level of education (Noubiap, Joko, Obama, & Bigna, 2013) .<br>- In Thailand, knowledge of the health card was a strong determinant of card purchase (Supakankunti, 2004)
Consumer understanding of concept of health insurance	**Total N= 15 studies (2 qualitative studies; 5 cross-sectional studies; 5 mixed studies; 3 case studies**<br><br><u>Barriers (n=10)</u><br>- In Guatemala and Philippine, fluctuation in memberships possibly resulted from the lack of familiarity with the insurance concept (Ron, 1999)<br>- In Ghana, community members did not understand the importance of an insurance scheme, which decreased enrolment rates (Nsiah-Boateng & Aikins, 2013).<br>- In Uganda, member dropout did not understand the purpose of the co-pay. Members of a now dissolved scheme expected to have their premiums returned if they did not access health services in a given quarter (Y. Derriennic, Wolf, K. & Kiwanuka-Mukiibi, P., 2005).<br>- In Uganda, lack of information on and poor understanding of the notion of community health insurance contributed to low enrolment in scheme (R. Basaza, Criel, & Van der Stuyft, 2007)<br>- In Uganda, a large section of the communities poorly understand the concept of pooling contributions. Thus, one of the reasons provided for not joining is that participants do not see how to benefit if they do not fall sick (R. Basaza, Criel, & Van der Stuyft, 2008)

	- In Tanzania, there was a failure among communities to see the rationale for protecting against the risk of illness often linking this to little knowledge about the benefits of the scheme (Kamuzora & Gilson, 2007).<br>- In Kenya, limited understanding of health insurance prevented people from becoming members(Mulupi, Kirigia, & Chuma, 2013).<br>- In Kenya, the concept of fund pooling was found to be poorly understood with many of the respondents believing that a member should be refunded the funds not utilized at the end of the subscription period. (Kamau & Njiru, 2014).<br>- In Tanzania, potential clients were confused when informed they would not get their contribution back at the end of the year if they had not utilized health services, thus misunderstanding the concept of risk pooling (Shaw, 2002).<br>- In Ghana, people failed to understand the concept of risk sharing , thus tending to withdraw from the scheme after a few years of not benefiting from the package (C. Atim, 2001).<br><br>Facilitator (n=5)<br>- In India, knowledge about insurance was significant and positive determinant for buying health insurance (Bhat, 2006)<br>- In Cameroon, 86.2% of respondents thought that belonging to a CBHI scheme could facilitate their access to adequate health care, and were thus willing to be involved in CBHI schemes (Noubiap et al., 2013)<br>- In India, women office bearers of scheme had gained a profound knowledge of health insurance and of inclusion issues (Haddad, Narayana, & Mohindra, 2011)<br>- In Tanzania, training workshops for beneficiaries allowed them to understand the schemes better (Kiwara, 2007)<br>- In Southeast Nigeria, awareness of the scheme offered financial risk protection and encouraged individuals to register (Onwujekwe et al., 2009)
**Attitude factors**	
Consumer trust in insurer	**Total N= 12 studies (1 qualitative study; 6 cross-sectional studies; 4 mixed studies; 1 case study**<br><br>Barriers (n=6)<br>- In Southeast Nigeria, some people did not register because they did not trust the insurer in regulating the funds (Onwujekwe et al., 2009).<br>- In Afghanistan, lack of faith in the resident doctor was among the top three reasons why households did not enrol (K. D. Rao et al., 2009)<br>- In Tanzania, suspicions were strong that government and providers wouldn't perform their expected role (Shaw, 2002)<br>- In Tanzania, lack of trust in CHF managers (managers lack transparency and are corrupt) influenced low enrolment (Kamuzora & Gilson, 2007).<br>- In Kenya, current and former members feel that the scheme administrators are insensitive to community's financial situation, and felt that administrators were not entirely accountable to the members on how scheme funds were managed (Kamau & Njiru, 2014).<br>- In Senegal, the majority of people who dropped out of CBHI did not take up opportunities to actively participate, did not trust the scheme staff or leaders, felt they were not able to hold the CBHI scheme to account (Mladovsky, 2014)

	Facilitators (n=6)<br>- In Lao PDR, CBHI members were more likely to place higher trust in the scheme (<0.001) (Alkenbrack et al., 2013).<br>- In Senegal, members were more likely than ex-members to be satisfied with the trustworthiness of scheme management and/or president (OR=4.01); (Mladovsky, 2014)<br>- In Cambodia, villagers who renewed the insurance scheme were found to have statistically significantly higher trust levels compared to those who were new to the scheme (RRR ¼ 1.07, p<0.001) and those who dropped out of the scheme (RRR ¼ 1.08, p< 0.001) (Ozawa & Walker, 2009)<br>- In Rwanda, the main determinant of participation in scheme is trust; people living near the facilities are more likely to enroll because they know the health center personnel, as well as the prepayment scheme management team (P. Schneider, & Diop, F., 2004)<br>- In Zaire, relationship of trust between the scheme and the population increased enrolment rate of the population (Criel & Kegels, 1997).<br>- In Ghana, a great deal of trust has been put in the treasurers and it is believed by the community members that "only the devil can influence the treasurer negatively" (C. Atim, 2001).
Sense of ownership of scheme	**Total N=5 studies (3 cross-sectional studies; 1 mixed study; 1 qualitative study)**<br><br>Barriers (n=1)<br>- In Kenya, only 10% of household members had a sense of ownership of scheme (Kamau & Njiru, 2014)<br><br>Facilitators (n=4)<br>- In Senegal, members were more likely than ex-members to think they could influence scheme operation (OR=2.32) (Mladovsky, 2014)<br>- In Uganda, a majority of the scheme members interviewed were involved in the mobilization of scheme members (Basaza, 2007)<br>- In Rwanda, the participatory approach and the democratic management of scheme lead to sentiments of "ownership" and increased trust among the poor, which are basic conditions for poor households to engage in any investment (P. Schneider, & Diop, F., 2004).<br>- In Uganda, members' perceptions of sustainability covered sense of ownership of their health programs without it being forced on them (Kyomugisha, Buregyeya, Ekirapa, Mugisha, & Bazeyo, 2009)
Satisfaction with services	**Total N= 5 (5 cross-sectional studies)**<br><br>Barriers (n=3)<br>- In Southeast Nigeria, respondents mentioned not being satisfied with skill of staff as what they did not like about the scheme (Onwujekwe et al., 2009).<br>- In Ghana, Members who are not satisfied from the scheme are less likely to renew their membership (Nsiah-Boateng & Aikins, 2013)<br>- In Burkina Faso, main reasons of respondents to motivate their decision to discontinue membership in CBHI scheme was not satisfied with services received (Dong, De Allegri, Gnawali, Souares, & Sauerborn, 2009)<br>Facilitators (n=2)

	- In Thailand, the cardholder group had greater satisfaction with the scheme than the non-cardholder group, strongly explaining the role of attitude (Supakankunti, 2004)<br>- In Thailand, high level of satisfaction of enrollees implied higher levels of card purchase (Supakankunti, 2000).
Perceived quality of care	**Total N=10 (5 cross sectional; 4 mixed studies; 1 case study)**<br><br><u>Barriers (n=6)</u><br>- In Afghanistan, some community members didn't want to enroll because of the perceived bad service quality (K. D. Rao et al., 2009).<br>- In Burkina Faso, factors such as perceived poor quality of care and providers' resistance may affect decision to enroll in CBHI scheme (Cofie, De Allegri, Kouyate, & Sauerborn, 2013b).<br>- In Burkina Faso, poor perceived quality of care (good vs. poor; OR=0.29; p=0.05) increased the probability that a household did not renew its membership in CBHI scheme (Dong et al., 2009)<br>- In India, members' poor perception of a preferred provider system (PPS) facility, or lack of familiarity with it, were also reasons for members not using the PPS (Ranson et al., 2006).<br>- In Kenya, non-members did not see any incentive to subscribe, since they felt CBHIS members did not receive preferential treatment (at the CBHIS clinics) over non-members (Kamau & Njiru, 2014).<br>- In Ghana, perceptions of some members about the quality of care they receive as compared to others (C. Atim, 2001)<br><br><u>Facilitators (n=4)</u><br>- In Senegal, members had a much higher probability of reporting that the quality of health service providers was satisfactory OR=5.54 (Mladovsky, 2014).<br>- In Southeast Nigeria, an important reasons given for willingness to renew registration in Igboukwu was good quality care offered (Onwujekwe et al., 2009).<br>- In Burkina Faso the overall perceived quality of care in Burkina Faso turned out to be significant determinant of utilization of health care services (Gnawali et al., 2009).<br>- In Lao PDR, CBHI households report a higher perception of quality of health care and positive experiences with CBHI at the district hospital (<0.001) (Alkenbrack et al., 2013).
Perceived financial risk protection	**Total N=4 (2 qualitative studies; 2 mixed studies)**<br><br><u>Facilitators (n=4)</u><br>- In Kenya, health insurance schemes were perceived to have many advantages by both members and non-members, including offering financial protection to members (Mulupi et al., 2013)<br>- In Guinea-Conakry, people reported adhering to the scheme because they believe the insurance helps preserve health and provides financial accessibility (Criel & Waelkens, 2003)<br>- In Uganda, new members invited others to subscribe so they can be insured and live without worry, and be helped in case of illness (R. Basaza et al., 2007)

	- In Nigeria, most respondents who registered did so because they perceived that the scheme offered financial risk protection (Uzochukwu et al. 2009
**Personal pre-disposition**	
Previous experience with local groups	**Total N=5 studies (2 cross-sectional studies; 3 mixed studies)**<br><br><u>Barriers (n=3)</u><br>- In Uganda, lack of trust in local financial organizations after previous depressing experiences with similar institutions contributed to low enrolment in scheme (R. Basaza et al., 2007)<br>- In Rwanda, suspicion towards the honesty of scheme leaders and previous negative experiences are important sources of mistrust that negatively affect enrolment (P. Schneider, & Diop, F., 2004)<br>- In Burkina Faso, lack of trust related to previous negative experiences with collective financial arrangements may affect CHI enrolment in scheme (Cofie et al., 2013b).<br><br><u>Facilitators (n=2)</u><br>- In Senegal, membership in other organizations was a positive factor toward membership in a health insurance scheme(Jütting, 2004)<br>- In Senegal, households enrolled in CBHI were significantly more likely to be members of other associations compared to non-CBHI households (Mladovsky, Soors, Ndiaye, Ndiaye, & Criel, 2014)
Affordability of care	**Total N= 4 studies (2 cross-sectional studies; 1 qualitative study; 1 case study**<br><br><u>Barriers (n=2)</u><br>- In Uganda, inability to pay for membership was pointed out as the foremost reason for not joining the schemes (R. Basaza et al., 2008)<br>- In Philippine, fluctuation in memberships due to drop outs at the end of the year may result from a change in household expenditure priorities in the holiday season (Ron, 1999)<br><br><u>Facilitators (n=2)</u><br>- In Rwanda, health insurance has tremendously improved the financial accessibility of its members to the modern health care system, particularly for women, children, and the poor (P. Schneider, & Diop, F., 2004)<br>- In Southeast Nigeria, the most important reason indicated for increased use of facility as well as willingness to renew registration was affordability of health care services through the scheme (Onwujekwe et al., 2009)
Health status	**Total N= 18 studies (1 RCT; 12 cross-sectional studies; 5 mixed studies)**<br><br><u>Barriers (n=4)</u><br>- In Ghana, household members who were not sick did not see the need to register (Nsiah-Boateng & Aikins, 2013)<br>- In Burkina Faso, introduction of premium subsidies led to insured group having significantly higher percentage of sick individuals, providing strong evidence for adverse selection, which put greater strain on financial viability of the scheme (Parmar, Souares, de Allegri, Savadogo, & Sauerborn, 2012)

	- In Central Kenya, some members did not see the reason of making contributions towards health insurance when they were in good health (Mulupi et al., 2013)<br>- In Burkina Faso, lower number of illness episodes in the past 3 months (OR=0.87) increased the probability that a household did not renew its membership in CBHI scheme (Dong et al., 2009)<br><br><u>Facilitators (n=15)</u><br>- In China, people who were less health were more likely to enroll in the scheme (Zhang & Wang, 2008)<br>- In Senegal, members reported worse health for every indicator, possibly indicating adverse selection (Mladovsky et al., 2014)<br>- In Thailand, significant factor related to card use was the presence of illness (Supakankunti, 2004)<br>- In Senegal, member households were twice as likely to have had an illness, accident or injury (OR=2), pointing to adverse selection (Mladovsky, 2014)<br>- In Senegal, the probability for women and older people participating in scheme is higher than for men and younger persons in the household since women of child-bearing age and older people do need hospitalization care more often than other household members (Jütting, 2004)<br>- In India, each additional illness reported within the past month (acute illnesses as well as exacerbations of chronic disease) was associated with a 70-80% increase in the probability of joining the scheme (Ranson, 2004)<br>- In India, maternity had increased the likelihood of enrollment (Gumber, 2004)<br>- In Thailand, families with illness tend to purchase and repurchase the card (Supakankunti, 2000)<br>- In China, individuals with poor health status were almost 1.6 times as likely to enroll in scheme as an individual with good health status (Wang, Zhang, Yip, & Hsiao, 2006)<br>- In Lao PDR, households in which a family member has either a chronic illness (<0.001) or had difficulty performing regular activities in the past three months (a proxy measure for illness) (p=0.008) were significantly more likely to enroll (Alkenbrack et al., 2013)<br>- In Central Kenya, ill health was a motivation for belonging to a scheme (Mulupi et al., 2013)<br>- In India, households with a higher ratio of sick members are more likely to purchase insurance (Ito, 2010)<br>- In India, households with children seem to be more risk averse and/or expect a higher need for health care and subsequently more likely to want to join insurance (Panda, Chakraborty, Dror, & Bedi, 2014)<br>- In Senegal, individuals reporting a health problem over the 30 day period preceding the interview were more likely to be enrolled in CBHI scheme (M. J. Ouimet, P. Fournier, I. Diop, & S. Haddad, 2007)<br>- In Nigeria, individuals who were actually in need of healthcare had a larger propensity to be insured (Lammers J, 2010).
**Socio-demographic factors**	
Age (older)	**Total N=8 Studies (6 cross-sectional studies; 3 mixed studies)**<br><br><u>Facilitators (n=8)</u>

	- In Senegal, older people bought insurance more than younger people (Mladovsky, 2014; Mladovsky et al., 2014)<br>- In Rural Armenia, usage of the scheme was higher with increasing age, along with health-issue reporting and health needs (Polonsky et al., 2009)<br>- In India, older age was significantly associated with membership (Ranson, 2004)<br>- In India, middle-aged groups (36-55) had a high chance of being enrolled, and a 5-7 times higher chance than the 16-25 age group (Gumber, 2004)<br>- In China, older residents, specifically those in the age groups of 45-54 (OR= (3.2210) and 55+ (OR= 1.8527), have a significant OR of larger than 1, thus they are more likely to enroll in scheme than the younger population (Wang et al., 2006)<br>- In China, middle age and elderly population were found more likely to enroll in the scheme than those between ages 16-35 (Zhang & Wang, 2008)<br>- In Burkina Faso, the household head's age was higher in those enrolled in the scheme (Dong et al., 2009)<br>- In Burkina Faso, there was a positive relation between respondents aged 36-54 and enrollment rate (Cofie et al., 2013a)
Gender (female)	**Total N=4 studies (3 cross-sectional studies; 1 mixed study)**<br><br><u>Facilitators (n=4)</u><br>- In Rural Armenia, women (52%) visited scheme more than men (45%), but men visited more frequently (Polonsky et al., 2009)<br>- In China, females were found more likely to enroll than men (Zhang & Wang, 2008)<br>- In China, the odds ratio for gender is significantly smaller than 1, which implies that female residents are more likely to enroll in the scheme than male residents in partially enrolled households (Wang et al., 2006)<br>- In Lao DPR, households enrolled were found to have more females, especially those of reproductive age and pregnant females (Alkenbrack et al., 2013)
Economic status (higher level)	**Total N=14 Studies (9 cross-sectional studies; 5 mixed studies)**<br><br><u>Barriers (n= 2)</u><br>- n Senegal, people of lower SES are more likely to enroll (M. J. Ouimet et al., 2007)<br>- In Thailand, poorer households were more likely to purchase cards since they cannot afford other types of schemes (Supakankunti, 2000)<br><u>Facilitator (n= 12)</u><br>- In Senegal, CBHI member households were wealthier (Mladovsky et al., 2014)<br>- In Nigeria, the propensity to be enrolled is seven times higher for persons from the highest quintile (Lammers J, 2010)<br>- In Senegal, although members of the CBHI scheme were wealthier and had higher expenditure levels than ex-members, the difference was not statistically significant (Mladovsky, 2014)<br>- In Senegal, wealthy people in the communities were more likely to participate in the insurance schemes (Jütting, 2004)<br>- In Central Kenya, people of high SES were more likely to join schemes (Mulupi et al., 2013)

	- In Rwanda, CBHI members were more likely to come from the higher income quartiles (P. Schneider, & Diop, F., 2004)<br>- In India, people belonging to the highest quintiles were found more likely to enroll (Gumber, 2004)<br>- In Lao DPR, better off families were more likely to join CHBI (Alkenbrack et al., 2013)<br>- In Burkina Faso, the richest quartile was found to have profited the most from membership in the insurance scheme compared the lowest quartile (Gnawali et al., 2009)<br>- In Burkina Faso, individuals from low SES households were less likely to enroll (Parmar et al., 2012)<br>- In China, less wealthy families were less likely to enroll (Zhang & Wang, 2008)<br>- In India, middle class had a higher enrolment rate, but their results were statistically insignificant (Ranson, 2004)
Employment status (employed)	**Total N=4 Studies (3 cross-sectional studies; 1 mixed study)**<br><br>Barriers<br>- In India, enrollment rate was found to be much lower in non-workers than those with home- based production or salaried workers (Gumber, 2004)<br><br>- <u>Facilitators (n=4)</u>In Thailand, households with a higher proportion of employed persons tended to purchase more cards than households with a lower proportion (Supakankunti, 2000)<br>- In Thailand, employment was a significant factor influencing card purchase (Supakankunti, 2004)<br>- In Lao DPR, working a non-agricultural job is correlated with higher enrollment (Alkenbrack et al., 2013)
Occupational Setting (rural)	**Total N=2 Studies (1 cross-sectional study; 1 mixed study)**<br><br><u>Barriers (n=1)</u><br><br>- In Burkina Faso, living in rural area increased the probability that a household did not renew its membership in CBI (Dong et al., 2009)<br><br><u>Facilitators (n=1)</u><br><br>- In Burkina Faso, residents in urban areas were 2.4 times less likely to have knowledge of the CHI scheme (Cofie et al., 2013a)
Education (higher level)	**Total N=10 Studies (7 cross-sectional studies; 3 mixed studies)**<br><br><u>Barriers (n=3)</u><br>- In India, the mean enrollment rate tend to decline with higher education, but education was found to be an insignificant predictor (Gumber, 2004)<br>- In Thailand, two studies showed that a low education rate was associated with a higher purchase of health card since lower education meant lower income and thus not covered by any other scheme (Supakankunti, 2000, 2004)<br><br><u>Facilitators (n=7)</u>

	- In Senegal, members were likely to be better educated, but the results were not statistically significant (Mladovsky et al., 2014)<br>- In Senegal, respondents enrolled in CBHI had significantly more schooling and were more literate (M. J. Ouimet et al., 2007)<br>- In Burkina Faso, a positive association was found between educational status and enrolment (Cofie et al., 2013a)<br>- In Burkina Faso, high education increased enrolment in the CBI (Gnawali et al., 2009)<br>- In China, residents with relatively higher education are more likely to enroll (Wang et al., 2006)<br>- In Lao DPR, those with vocational training or post-secondary education were more likely to enroll (Alkenbrack et al., 2013)<br>- In Burkina Faso, drop-outs were found to have a significantly lower education level (Dong et al., 2009)
Religion (Christian)	**Total N= 4 Studies (2 cross-sectional studies; 2 mixed studies)**<br><br><u>Barriers (n=2)</u><br>- In Senegal, Muslims were more likely to enroll (M. J. Ouimet et al., 2007)<br>- In Nigeria, being Christian reduced the propensity to insure (Lammers J, 2010)<br><br><u>Facilitators (n=2)</u><br>- In Senegal, religion played an important role where there was higher participation by Christians (the probability increases by nearly 40 percentage points) compared with that for non-Christians (Jütting, 2004)<br>- In Burkina Faso, the dropouts were more likely to be non-muslims (Dong et al., 2009)
Ethnicity (minority)	**Total N=3 studies (1 cross-sectional study; 1 mixed study; 1 case study)**<br><br><u>Barriers (n=3)</u><br><br>- In Burkina Faso, belonging to the Bwaba ethnic group showed a lower enrollment rate (Gnawali et al., 2009)<br>- In India, Paniyas (a marginalized indigenous group) were the least likely to be included in the scheme (Haddad et al., 2011)<br>- In Lao DPR, residents of non-CBHI districts were significantly more likely to belong to an ethnic minority (Alkenbrack et al., 2013)
Migration	**Total N=1 study (1 cross-sectional study)**<br><br><u>Barrier (n=1)</u><br><br>- In china, it was shown that rural-to-urban migrant workers are less likely to be enrolled in a CBHI (Zhang & Wang, 2008)

Per capita expenditure (higher level)	**Total N=4 studies (3 cross-sectional studies; 1 mixed study)**<br><br>Barriers (n=1)<br>- In Burkina Faso, higher household expenditure increased drop-out rates (Dong et al., 2009)<br><br>Facilitators (n=3)<br>- In Senegal, CBHI households had significantly higher levels of expenditure than non-member households (Mladovsky, 2014)<br>- In Senegal, members of the CBHI scheme had higher expenditure levels than ex-members but the difference was not statistically significant (Mladovsky et al., 2014)<br>- In Burkina Faso, high per capita expenditure implied increased enrolment rates (Gnawali et al., 2009)
Marital status (married)	**Total N= 4 studies (2 cross-sectional studies; 2 mixed studies)**<br><br>Facilitators (n=4)<br>- In India, enrolment rate was higher among currently married women (Gumber, 2004)<br>- In China, married and those who are divorced or widowed are more likely to join the scheme than those who are single (Zhang & Wang, 2008)<br>- In Lao DPR, those enrolled were more likely to be married (Alkenbrack et al., 2013)<br>- In Senegal, respondents enrolled in CBHI were more likely to be married (M. J. Ouimet et al., 2007)

2-Interpersonal level

Interpersonal level	
**Household dynamic**	
Household size	**Total N= 10 studies (1 qualitative study; 6 cross-sectional studies; 2 mixed studies; 1 case study)**<br><br>Barriers (n=6)<br>- In Burkina Faso, larger households were less likely to enroll in CBI (Gnawali et al., 2009).<br>- In Burkina Faso, households in the drop-out group had a significantly higher household size (p=0.0015) (Dong et al., 2009)<br>- In Senegal, several non-members said they had not enrolled in CBHI because they could not afford to pay the premium for their extended kin (Mladovsky et al., 2014).<br>- In India, household size showed an inverse relationship with enrollment, and the odds ratios tended to decline significantly in medium-size and large households (Gumber, 2004)<br>- In Guinea Conakry, respondents with large families said that the household subscription is too large a burden for big families to bear (Criel & Waelkens, 2003)<br>- In Uganda, one of the reasons most mentioned for not joining the schemes is being unable to pay contributions for large families (R. Basaza et al., 2008)<br><br>Facilitators (n=4)<br>- In La PDR, CBHI households are larger (5.3 vs. 4.7; p<0.001) (Alkenbrack et al., 2013)<br>- In China, residents with a large family are more likely to be enrolled in the scheme than the residents with a small family (Wang et al., 2006).<br>- In Rwanda, households with five and more members are 60% more likely to buy insurance than smaller households. This was attributed to the possibility of signing up in a CBHI plan as a family of up to seven members for the same annual premium (P. Schneider, & Diop, F., 2004)<br>- In Nigeria, persons from larger families are more likely insured (Lammers J, 2010)
Household head characteristic	**Total N= 6 studies (1 RCT; 5 cross-sectional studies)**<br><br>Barriers (n=3)<br>- In Burkina Faso, having a female household head was found to be a barrier to enrolment. Similarly, household heads in the drop-out group had a significantly lower education than in the non-drop-out group (Dong et al., 2009)<br>- In Kenya, the scheme had very few members from female-headed households as well as youth, since these are more likely to be on the lower-income percentile in the study society due to its paternalistic orientation (Kamau & Njiru, 2014).

	- In Burkina Faso, households with younger heads were less likely to enroll in CBI (Gnawali et al., 2009).<br><br>Facilitators (n=3)<br>- In Sri-Lanka, female-headed households as well as household heads with some primary education, were more likely to be members of the scheme Bending 2011<br>- In Rwanda, insured households are more likely to be headed by a male (Schneider, 2004)<br>- In India, household heads with some primary education, as compared with those who are illiterate, are more likely to join a CBHI scheme (Panda
**Peer influence**	**Total N=4 studies (1 quantitative study; 3 mixed studies)**<br><br>Facilitators (n=4)<br>- In Senegal, members were more likely than ex-members to have heard of the scheme from a family member or friend compared to another source (Mladovsky, 2014).<br>- In Lao PDR, CBHI members were more likely to have more close relatives and friends in the scheme (<0.001) (Alkenbrack et al., 2013)<br>- In Burkina Faso, community leaders indicated that rural inhabitants tended to validate information from their relatives in urban areas, which meant that a clear understanding of CBHI concept by urban dwellers would be vital to decision making and adoption of scheme by rural residents (Cofie et al., 2013a)<br>- In Thailand, continuity of card purchase in the study was associated with persuasion by a neighbor to buy a card (Supakankunti, 2004)
**Social solidarity**	**Total N= 8 studies (3 cross-sectional; 4 mixed; 1 case study)**<br><br>Barriers (n=1)<br>- In Senegal, most of the individuals who dropped out of CBHI scheme did not know many other members and did not believe that CBHI promotes solidarity (Mladovsky, 2014).<br><br>Facilitators (n=7)<br>- In Cameroon, 68.7% of informal sector workers belonged to a solidarity based community association, the latter which could reflect one's willingness to join a CBHI scheme. (Noubiap et al., 2013)<br>- In Central Kenya, household members reported that a perceived advantage of the scheme was building on solidarity to help other community members (Mulupi et al., 2013).<br>- In Senegal, content analysis of qualitative data showed that subscribers consider solidarity as the most important aspect of CBHI scheme (M. J. Ouimet et al., 2007)<br>- In Senegal, members were more likely to have more solidarity than ex-members. Furthermore, believing that the community would cooperate in an emergency was significantly positively correlated with enrolment (Mladovsky et al., 2014)<br>- In Rwanda, local initiatives (via churches and members who attended the scheme general assemblies) have helped to pay enrollment fees for

	indigents, widows, orphans, and poor high-risk patients (P. Schneider, & Diop, F., 2004)<br>- In Nigeria, some individuals paid the premium for other members of the community, where about 77.9% of those registered showed the willingness to register for other members which contributed to increased enrolment in community (Uzochukwu et al., 2009)<br>- In Ghana, there is a common understanding that people should pay when they can do so, under their own free will since they all have no steady source of income. In this scheme, there is hardly any defaulting, for though people may not have been regular at meetings, they still send their contribution (C. Atim, 2001a)

3- Community level

Community level	
**Role of culture**	**Total N= 2 studies (1 cross-sectional study; 1 qualitative study)**<br><br>Barriers (n=2)<br>- In Kenya, most residents believe and practice herbal medicine; conventional medicine is seen as a last resort. The situation is made worse by the fact that money is not always the way of transacting herbal medicine since barter trade is still rampant in this society (Kamau & Njiru, 2014)<br>- In Uganda, a reason provided by household members or not joining the scheme was that joining is associated with inviting disease (R. Basaza et al., 2008)
**District-level characteristics**	**Total N= 1 study (1 mixed study)**<br><br><u>Facilitators (n=1)</u><br>- In Lao People's Democratic Republic (Lao PDR), relative to non-CBHI districts, CBHI districts have a significantly higher population density, lower poverty rates, higher literacy rates, and a higher proportion of the population working in the non-agricultural sector, more likely to have electricity (Alkenbrack et al., 2013)
**Community involvement in decision-making**	**Total N=11 studies (5 qualitative studies; 5 mixed studies; 1 case study)**<br><br><u>Barriers (n=5)</u><br>- In Uganda, low level of community involvement in the management of hospital-based CBHI schemes contributed to low enrolment in scheme (R. Basaza et al., 2007)<br>- In Uganda, members of the scheme were not involved in the decision-making process on the scheme which discouraged enrolment (R. Basaza et al., 2008)<br>- In Uganda, perceived value of the health schemes can be shaken when there is low community participation in decision making resulting in decreased support of the scheme and a consequent decrease in scheme membership and cost recovery (Y. Derriennic, Wolf, K. & Kiwanuka-Mukiibi, P., 2005)<br>- In Senegal, implementation has been slow and laborious, with more attention needed to increase member participation in the processes involved in implementing (M. J. Ouimet et al., 2007)<br>- In Nigeria, community participation was very poor in community B as a result of lack of proper mobilization of the community by the managers and health workers (Uzochukwu et al., 2009)<br><br><u>Facilitator (n=6)</u><br>- In Uganda, key informants observed that members' involvement in planning and decision making was crucial in sustaining CHI schemes, with members being informed about already made decisions by the top management of the schemes (Kyomugisha et al., 2009)<br>- In Philippine, cooperative framework with its built-in members' participation mechanisms, appeared to be a major factor in finding the optimal administrative base

	for such voluntary scheme than a one-sided decision by a health care financing scheme without member or community participation- (Ron, 1999)<br>- In Rwanda, a positive reported aspect was involvement of community members in the development of the MHI scheme (P. Schneider, 2005)<br>- In Senegal, CBHI members were more than two times as likely to report having control over decisions made in the community or by their neighbours which affected their daily life compared to non-members. Active community participation is negatively correlated with drop-out (Mladovsky, 2014)<br>- In Guinea-Conakry, the intense period of preparation and the genuine sense of community participation that was incorporated from the start have substantially contributed to strong impression of trust and also helped develop confidence in the capacities of local managers (Criel & Waelkens, 2003)<br>- In Burkina Faso, community participation was positive and significant determinants of enrolment (Cofie et al., 2013a)

4. Systems level: Governance arrangement

Governance arrangement level	
**Stakeholder involvement**	**Total N=4 studies (2 qualitative studies, 1 case study)**<br><br>Barriers (n= 3)<br>– In Guatemala, the slow and problematic development of the scheme was influenced by conflict over health care provision by church-affiliated institutions (Ron, 1999).<br>– In Tanzania, the introduction of the scheme policy at the central level with little input from district managers resulted in the managers perceiving the implementation process as imposed and rushed with little time to prepare (Kamuzora & Gilson, 2007).<br>– In Guinea-Conakry, poor involvement of health professionals in scheme design contributed to low support of scheme implementation (Criel & Waelkens, 2003)
**Political economy context**	**Total N= 6 studies (1 qualitative study; 3 mixed studies; 2 case studies)**<br><br>Barriers (n= 5)<br>– In Zaire, socio-economical unrest caused a fall in enrolment and funding (Criel & Kegels, 1997)<br>– In Tanzania, Slow uptake in membership to deteriorating agricultural performance between 1997-98, meant that success of the scheme is partially dependent on macro-economic performance (Shaw, 2002)<br>– In Uganda, the falling value of the 'Cedi' and the dollarization of the economy was a threat to the survival and growth of the fund (C. Atim, 2001c)<br>– In Nigeria, the Governor was removed as a result of political tensions in the state, and the Commissioner left with him. This was followed by a decline in state interest in and support for the CBHI scheme, with no subsequent expansion of the scheme (Uzochukwu et al., 2009)<br>– In Uganda, CBHI scheme was sometimes a controversial and politically sensitive issue, where user fees have been abolished in the public sector following a decision by the president (R. K. Basaza, Criel, & Van der Stuyft, 2010)<br><br>Facilitator (n= 1)<br>– In Senegal, members believed CBHI schemes were managed in a democratic manner, perhaps helping to explain why voting (60% of respondents reported voting in the last local elections) was correlated with enrolment (Mladovsky, 2014)
**Government support**	**Total N= 7 studies (4 qualitative studies, 2 mixed studies, 1 case study)**<br><br>Barriers (n=2)<br>– In Uganda, the government did not provide of policy, legislative, technical and regulative support, with no clear policy and implementation guidelines which resulted in low enrolment in scheme (R. Basaza et al., 2007).<br>– In Uganda, the absence of clear national guidelines on health care financing hindered scheme progress (R. K. Basaza et al., 2010)

	<u>Facilitators (n=5)</u><br>– In Tanzania, government was able to draw on funds from a World Bank loan for cost-sharing (Shaw, 2002)<br>– In China, government paid the full premium for those who cannot afford the premium (Wang et al., 2006)<br>– In Uganda, more support from both NGOs and government was reported as important by CHI scheme members and non-members in order to slowly move towards sustainability and sufficiently meet the health needs of the communities (Kyomugisha et al., 2009)<br>– In Guatemala and Philippine, it was suggested that the schemes could benefit from an "umbrella organization" to provide support in design, training and information services as well as involve government, non-government and academia, as an integral part of the development process (Ron, 1999)<br>– In Rwanda, the issuance of an officially stamped scheme membership card in return for paying annual premium was suggested as the only way to trust the scheme (P. Schneider, 2005)
**Management and administrative structure**	**Total N= 12 studies (3 qualitative studies, 2 cross-sectional studies, 4 mixed studies, 3 case studies)**<br><br><u>Barriers (n=8)</u><br>– In India, win leadership of CBHI have led to an increase in equity in managing the scheme (Haddad et al., 2011)<br>– In Guatemala the scheme was not sufficiently established as an administrative body at the conceptual stage; by the time the necessary action was taken, local conflicts hindered progress (Ron, 1999)<br>– In Uganda, lack of adequate financial accounting systems to provide proper separation of scheme accounts from the hospital's accounts prevented effective decision making by scheme managers (Y. Derriennic, Wolf, & Kiwanuka-Mukiibi, 2005)<br>– In Senegal, managerial difficulties led to a suspension of operations for some time; consequently, several people left the scheme (Jütting, 2004)<br>– In Tanzania, district managers did not allocate a budget for scheme administration activities. Policy guidelines prohibited district managers from using scheme funds for administration; district managers saw the scheme as an additional and separate activity from their routine work (Kamuzora & Gilson, 2007)<br>– In Uganda, the scheme lacked its own accountant, so all its finances were handled by the St. Theresa's Hospital accountant, thus it has lacked the needed autonomy it should have (C. Atim, 2001a)<br>– In Uganda, inexperience of the district medical scheme leading the project and lack of vital managerial skills for running an insurance scheme (C. Atim, 2001a)<br>– In Guinea-Conakry, participants mentioned the need for opportunities for appealing against a negative decision or event that are included in constitution of their associations (Criel & Waelkens, 2003)<br>– In India, household members pointed to a variety of administrative problems with the schemes which could hinder enrolment (Ranson et al., 2006)<br><br><u>Facilitators (n=5)</u><br>– In Philippine, the major success factor are sound administrative structure provided by a cooperative and controls in the delivery system and in expenditures (Ron, 1999)

	– In Tanzania, embedding the management of scheme fund into the existing district health management arrangements controlled by government (combining skilled government workers with strong community representation) made it possible to jointly sustain management functions of scheme and assure "public accountability" to the satisfaction of the Ministry of Health (Shaw, 2002)<br>– In Rwanda, members and non-members suggested that "scheme managers must be "close to the population, learn about people's problems, and inform people about the scheme; and they must defend members' interest when negotiating with providers for better quality care" in order to respond to consumer needs and patient concerns (P. Schneider, & Diop, F., 2004)<br>– In Senegal, accountability and being informed of mechanisms of controlling abuse/fraud are all correlated with remaining in the scheme (Mladovsky, 2014)<br>– In Cameroon, 53.3% preferred management by missionaries (Noubiap et al., 2013)
**Capacity of insurer promoters**	**Total N=3 studies (2 mixed study, 1 case study)**<br><br><u>Barriers (n=2)</u><br>– In Guatemala, the slow and problematic development of the scheme was compounded by lack of knowledge and experience of stakeholders in making decisions regarding a viable CBHI scheme (Ron, 1999)<br>– In Uganda, the limited expertise on CBHI within the Ministry of Health and amongst donors, with little or no practical experience in setting up CHI schemes were reported to affect low enrolment (R. Basaza et al., 2007)<br>Facilitator (n=1)<br>– Good leadership that can support schemes to start income generation activities and attract more members was reported to promote sustainability of scheme(Kyomugisha et al., 2009)
**Package content and membership criteria**	
Package content	**Total N=9 studies (2 qualitative studies, 4 cross-sectional studies, 2 mixed studies, 1 case study)**<br><br><u>Barriers (n= 6)</u><br>– In Guatemala and Philippine, periodic attention should be given to adjusting and extending benefits to deal with the changing needs and preferences of the insured population (Ron, 1999)<br>– In Ghana, the main disadvantage reported by current CBHI scheme members was lack of certain essential services at the clinic such as inpatient and X-ray components (Kamau & Njiru, 2014)<br>– In Tanazania, entitlements in the CBHI benefit package were limited to preventive and selected curative care services at health centers or out-patient departments at local hospitals (Shaw, 2002)<br>– In Uganda, CHI scheme members and non-members reported that the scheme did not illnesses like diabetes and hypertension which influenced their perceptions of unfairness with regard to the role of CHI schemes (Kyomugisha et al., 2009)<br>– In China, poor families who have been enrolled in scheme stated that setting limitations on disease eligibility of scheme had limited poor families to benefit more from scheme (Hao, 2010)<br>– In Uganda, the exclusion of treatment of chronic diseases in the benefits package of the schemes contributed to low enrolment (R. Basaza et al., 2008)<br><br><u>Facilitators (n=5)</u>

	– In Rwanda, the prepayment schemes' features, including benefit package, were designed, discussed, and agreed upon (by voting) in a series of about 30 workshops in the three districts attended by the local populations. As a result, the schemes were "tailored" to the needs of the local people (P. Schneider, & Diop, F., 2004)<br>– In Thailand, the absence of limit on episodes and the first contact at either a health center or community hospital increased the rate of service usage, especially at community hospitals (Supakankunti, 2000)<br>– In China, benefit packages that included chronic diseases had significant associations with frequency of scheme use (β = 0.01) (Hao, 2010)<br>– In Uganda, scheme treatment is provided for all diseases, which encouraged enrolment (R. Basaza et al., 2008)<br>– In Kenya, inadequate benefit packages hindered people from joining health insurance schemes and/or contributed to drop out rates. Household members preferred a comprehensive benefit package that included inpatient and outpatient care with no co-payments (Mulupi et al., 2013)
Membership size	**Total N= 3 studies (2 qualitative studies and 1 cross-sectional study)**<br><u>Barriers (n=2)</u><br>– In Uganda, schemes are not sustainable because they have low enrolment because some health workers are rude; some members keep dropping out of the scheme.(Kyomugisha et al., 2009)<br>– In Uganda, a scheme's financial viability and therefore its sustainability depend greatly on the size of its membership. A strong membership base requires scheme promotion and marketing to attract new members and membership policies to retain current members(Y. Derriennic, Wolf, K. & Kiwanuka-Mukiibi, P., 2005).<br><br><u>Facilitator (n=1)</u><br>– In Thailand, sustainability of the program depends on various factors, a very important one being is satisfaction of the card users to continue to buy. The findings indicate that the continuity of card purchase in the study was significantly associated with these factors: persuasion by a neighbor to buy a card, age, education, income, health center personnel explained clearly about card, and persuasion of a household to buy the card (Supakankunti, 2004)
Membership criteria	**Total N= 10 studies (4 qualitative studies, 3 cross-sectional studies and 3 mixed studies)**<br><u>Barriers (n=7)</u><br>– In China, adverse selection within partially enrolled households may affect the financial sustainability of the scheme (Wang et al., 2006)<br>– In Burkina Faso, the definition of household does not adequately reflect all decision-making processes in individuals' everyday life which impacts decision to enroll (De Allegri, Sanon, Bridges, & Sauerborn, 2006)<br>– In Nigeria, members can only enroll if they are part of a 500 population community which affected success in implementing the scheme (Onwujekwe et al., 2009)<br>– In Uganda, individuals could enrol if they are members of an already existing community-based organization and at least 60% of the organisation's members had to join before they could start accessing health services, which in turn afffected community memebers' perceptions of sustainability with regard to the CBHI scheme (Kyomugisha et al., 2009)<br>– In Uganda, one of the key issues affecting enrolment was th e difficulties to raise raise the required number in the group/village before enrolment (60% of a group in the

	Ishaka scheme or 100 families per village in Save for Health-Uganda (SHU) scheme (R. Basaza et al., 2008)<br>– In Uganda, difficulties for existing community groups to raise 60% of the membership or 100 families per village prior to enrolment (R. Basaza et al., 2007)<br>– In Guinea-Conakry, while all respondents seem to be aware that the household and not the individual is the subscription unit, this requirement is only perceived as a restriction of membership (Criel & Waelkens, 2003)<br><br><u>Facilitators (n=5)</u><br>– In Burkina Faso, the majority of household heads acknowledged the value of insuring the whole household as they recognized the possible limitation of individual enrolment in relation to equity and completeness of financial protection that only household enrolment can secure (De Allegri et al., 2006)<br>– In Uganda, non-discriminatory and voluntary nature of joining the scheme allowed people to join irrespective of family background (Kyomugisha et al., 2009)<br>– In Ghana, Generally, adverse selection, which is considered a common risk, associated with most MHOs is very minimal with this scheme, due to the idea of compulsory membership by every member of the community (C. Atim, 2001a)<br>– In Ghana, Registration is on family basis. This means that, once a person (often a key person in the family) decides to join, then all members of that family must register. This is to avoid the risk of adverse selection. A family card is issued to a family that has registered, with personal data of each member of the household provided on the form, as well as a photograph of each member (C. Atim, 2001a)<br>– In Senegal, registration is on family basis which meant that, once a person decides to join, then all members of that family must register. This is to avoid the risk of adverse selection *(M.-J. Ouimet, P. Fournier, I. Diop, & S. Haddad, 2007)*<br>– In Rwanda, the possibility of signing up in a CBHI plan as a family of up to seven members for the same annual premium might have been an incentive for larger households to enroll with all their family members (P. Schneider, & Diop, F., 2004)

5. Systems level: Financial arrangement

Financial arrangement level	
**Amount and timing of premium**	**Total N= 19 studies (4 qualitative studies, 4 cross-sectional studies, 8 mixed studies, 3 case studies)**<br><br>Barriers (n=18)<br>- In Guinea, a problem of affordability for many poor who cannot raise enough money to pay the subscription was reported (Criel & Waelkens, 2003).<br>- In Burkina Faso, the amount of premiums to be paid remains unaffordable for very poor households (De Allegri et al., 2006).<br>- In Ghana, respondents reported that premium level is very expensive which is affecting enrolment negatively(Nsiah-Boateng & Aikins, 2013).<br>- In Lao PDR, the most frequent reason for never enrolling in CBHI was the inability to afford the premiums (Alkenbrack et al., 2013).<br>- In Afghanistan: High premiums decreased enrolment rate (K. D. Rao et al., 2009).<br>- In Tanzania, Inability to pay annual contributions was identified as an important barrier preventing poor households from joining the CBHI (Kamuzora & Gilson, 2007).<br>- In Tanzania, Igunga and Singida Rural districts, user fees were not high enough to motivate people to save and risk pool; therefore prepayment was not sufficiently attractive (Shaw, 2002).<br>- In Samburu district in northern Kenya, people they felt that the cost of being a member of a scheme was way too high although there is an option of paying in instalments (Kamau & Njiru, 2014).<br>- In Northwest Cambodia, timing of collecting the contributions (e.g., monthly, quarterly, annually) was considered important for the community members to get their CBHI cards before they pay for any premium(Ozawa & Walker, 2009) .

	- In Nouna Health District, rural Burkina Faso, respondents reported that the main reasons of to motivate their decision to discontinue membership in CBI were 'could afford no longer' (Dong et al., 2009).<br>- In Fengsan Township, Guizhou Province of China, high premium rate were reported to negatively influence enrolment rates (Zhang & Wang, 2008).<br>- In Anambra state, southeast Nigeria, the cost of registration was high and premium is retrogressive meaning the poor were affected by the premium amount leading to inequity (Onwujekwe et al., 2009).<br>- In Central and Southern Uganda, problems in the ability to pay the premium influenced enrolment. Also, it was reported that schemes didn't have health care subsidies for the poorest sectors of the population (R. Basaza et al., 2007).<br>- In Uganda, the most vulnerable and needy in society such as orphans, the elderly and the disabled, are not exempt from payment, even though they usually have greater health needs than the rest of the population (Kyomugisha et al., 2009).<br>- In Burkina Faso, respondents reported that current payment modalities, requiring that the premium is paid all at once for the entire household, constituted an important barrier to enrolment(De Allegri et al., 2006).<br>- In Nyeri and Kirinyaga districts, Central Kenya, not being able to pay in instalments was another reason given that made it difficult for people to join CBHIs(Mulupi et al., 2013)<br>- In Tanzania, Drop out in individual-based premiums was higher than that in group-based premiums (Kiwara, 2007).<br>- In Zaire and Tanzania, variations in enrolment rates have been attributed to varying agricultural performance since pre-payment contributions were made once yearly at harvest time (Criel & Kegels, 1997; Shaw, 2002).<br>- In Guatemala, Philippine, and Kenya, setting affordable contribution rates adjusted at reasonable intervals to reflect changes in benefits, health costs and inflations facilitated enrolment (Mulupi et al., 2013; Ron, 1999).<br>- In India, the application of uniform enrollment policies for all enrollees resulted in inequity amongst the most vulnerable populations, further perpetuating their poverty and social marginalization (Haddad et al., 2011).<br><br><u>Facilitators (n=4)</u><br>- In Central and Southern Uganda, Payment by installment was an important enabling factor to enrolment (R. Basaza et al., 2007).<br>- In Nyeri and Kirinyaga districts, Central Kenya, one of the factors that made it easy for people to belong to health insurance schemes was affordable contribution rates. Another factor was favourable contribution mechanisms, where members were allowed to make their contributions in instalments or having them linked to agricultural produce (Mulupi et al., 2013).<br>- In Burkina Faso, lower premium level for children compared to adults seen as an enabling factor to enrolment (De Allegri et al., 2006).<br>- In Tanzania, mutual cells were established to make it easy for beneficiaries to encourage each other to pay premiums or act as pressure groups for group leaders to pay premiums (Kiwara, 2007).
**Cost-sharing**	**Total N= 8 studies (1 qualitative studies; 6 cross-sectional studies; 1 mixed study)**<br><br><u>Barriers (n= 8)</u><br>- In rural Gujarat, India, even under the preferred provider system, user fees continue to pose a financial barrier to the insured. They have to mobilize funds to cover the

	costs of medicines, supplies, registration fee, etc. before receipt of cash payment from scheme (Ranson et al., 2006).<br>- In Uganda , out-of-pocket expenditure remains an important feature of health care financing despite blanket abolition of user fees in government facilities (R. K. Basaza et al., 2010).<br>- In China, reimbursement rate of hospitalization increased from 40~70% to 60 ~ 80%, and for some special cases, services were free. But for most ordinary MFA cardholders, 20~40% co-payments for hospitalization costs still brought a huge economic burden to these poor families. Also, ceilings and deductibles for reimbursement of inpatient services served as obstacles for poor families' access to health care (Hao, 2010).<br>- In Central Kenya, high co-payments hindered people from joining health insurance schemes and contributed to drop out rates (Mulupi et al., 2013).<br>- In China, high copayment rate was reported to hinder people from joining health insurance schemes (Zhang & Wang, 2008).<br>- In Rwanda, out-of-pocket spending per episode of illness was significantly influenced negatively if patients lived in the health center's vicinity and if they owned cattle (P. Schneider, & Diop, F., 2004)<br>- In India, out of pocket expenditures were mainly attributed to transport, medicine and pre-diagnostic investigations, highlighting a scope for the scheme to improve strategic purchasing (M. Rao et al., 2012).<br>- In Ghana, no co-payment causes an increase in medical bills expenditure which in turn could pose problems in the sustainability of the scheme (Nsiah-Boateng & Aikins, 2013)<br><br><u>Facilitators (n=3)</u><br>- In Thailand, the introduction of cost-containment measures was highlighted as necessary to reduce escalating cost of medical claims and decrease overutilization of services, which in turn could pose threats to the sustainability of CBHI schemes (Supakankunti, 2004)<br>- In Uganda, the community group's decision to raise the co-pay to combat overutilization of health services is a positive example of how a sensitized member base can make educated decisions to protect the financial viability of the scheme(Y. Derriennic, Wolf, K. & Kiwanuka-Mukiibi, P., 2005)
**Payment arrangement for services**	**Total N= 5 studies (1 qualitative study; 1 cross-sectional study; 3 mixed studies)**<br><br><u>Barriers (n= 4)</u><br>- In Tanzania, public and private health providers viewed the capitation payment associated with CBHI scheme as a potentially appealing alternative to collecting user fees, often at times when people were unable to pay (Shaw, 2002).<br>- In Burkina Faso, providers perceived the insufficient levels of capitation payments, infrequent schedule of capitation payment, and lack of a mechanism for reimbursing service fees (as opposed to only drugs) as significant sources of dissatisfaction and loss of motivation (Robyn et al., 2014).<br>- In Ghana, delays in provider claims negatively influenced service delivery to insured members (Nsiah-Boateng & Aikins, 2013).<br>- In Khon Kaen Province, Thailand ,insufficient re-imbursement of expenses shifted the cost burden to providers creating inequities in service delivery (Supakankunti, 2004).<br><br><u>Facilitators (n=2)</u>

	- In Tanzania, public and private health providers viewed the capitation payment associated with CBHI scheme as a potentially appealing alternative to collecting user fees, often at times when people were unable to pay (Shaw, 2002).<br>- In Uganda, the abolition of user fees in public sector gave rise to the practice of "under-the-table" payments, potentially impeding improvements in service delivery (R. K. Basaza et al., 2010).
**Financial viability**	**Total N=9 studies (2 qualitative studies 2 cross-sectional studies; 4 mixed studies; 1 case study)**<br><br>Barriers (n= 7)<br>- In Burkina Faso, an increased financial volatility of health facilities was reported with the majority of head nurses raised the concern of facility bankruptcy, and the quality sacrifices they had to make to avoid bankruptcy(Robyn et al., 2014).<br>- In Ghana, Unforeseen financial implications which had not been budgeted for future rises in health care costs made it difficult for the association to meet the needs of its members in situations where people will present ill at the same time, or in serious cases of illness such as conditions requiring surgery (C. Atim, 2001a).<br>- In Senegal, irregularity of contributions was seen as the greatest threat to financial sustainability(M. J. Ouimet et al., 2007).<br>- In Ghana, respondents reported that decreased contributions of the informal sector can affect the sustainability of the insurance scheme (Nsiah-Boateng & Aikins, 2013).<br>- In Samburu district, northern Kenya, CBHI members' contribution could only fund 10% of the total running costs while the rest came from donor's subsidies. This means that without the subsidies, the premiums would not be able to run the scheme sustainably(Kamau & Njiru, 2014).<br>- In Khon Kaen Province, Thailand, the problem of card overutilization was reported to have implications for the sustainability and efficiency of the program. The results of the study showed that among card users 41.6 percent tended to visit health facilities more than before having a card, 48.4 percent the same as before, only 7.2 percent less than before, and 2.8 percent do not remember(Supakankunti, 2004).<br>- In Uganda, they operate on small budgets, and lack government support (Kyomugisha et al., 2009).<br><br>Facilitator (n=2)<br>- In Tanzania, additional monies and local purchasing power for health could potentially enhance sustainability (Shaw, 2002).<br><br>- In Uganda, financial sustainability can be achieved through successfully identifying a target population and setting appropriate premiums and co-payments to attract members, contributing to cost recovery and overall financial viability (Y. Derriennic, Wolf, K. & Kiwanuka-Mukiibi, P., 2005)

6. Systems level: Delivery arrangement

Delivery arrangement level	
**Human resource planning and management**	
Human resource planning	**Total N= 5 studies (2 qualitative studies, 2 mixed studies, 1 case study)**<br><br>Barriers (n=4)<br>– In Nigeria, 22.9% of those unwilling to renew from Igboukwu indicated the absence of a doctor at the facility as a reason (Onwujekwe et al., 2009)<br>– In Ghana, non- availability of permanent staff was reported by household member to contribute to the low enrolment rates (C. Atim, 2001b)<br>– In Tanzania, average and wealthy groups indicated that staff shortages affected their perception of quality of services, which influenced low enrolment (Kamuzora & Gilson, 2007)<br>– In Nigeria, absence of doctors in health centers constrained scheme implementation (Uzochukwu et al., 2009)<br><br>Facilitators (n=2)<br>– In Southeast Nigeria, availability of health personnel was reported by household as a reason for their increased facility utilization (Onwujekwe et al., 2009)<br>– In India, the beneficiary satisfaction survey elicited the highest scores for doctors and nurses (M. Rao et al., 2012)
Human resource management	**Total N= 7 studies (3 qualitative studies, 1 cross-sectional study, 2 mixed studies, 1 case study)**<br><br>Barriers (n=5)<br>– In Tanzania, insufficient supervision of health staff by district managers raised concerns among community members about the improper provision of services by staff including absenteeism during working hours (Kamuzora & Gilson, 2007)

	– In Burkina Faso, the lack of an existing payment mechanism linked to CBHI enrollment led to low health worker satisfaction, work-related motivation, and support for the CBHI (Robyn et al., 2014).<br>– In Lao PDR, both members and non-members reported that health care staff members do not have the skills to diagnose health problems and that productivity is low (Alkenbrack et al., 2013)<br>– In Southeast Nigeria, respondents reported staff incompetence in facilities as influencing enrolment and utilization of services (Onwujekwe et al., 2009)<br>– In Rwanda, insured and uninsured respondents complained that health facilities lack qualified personnel (P. Schneider, 2005)<br><br>Facilitators (n=2)<br>– In Philippines, health care workers became increasingly interested in achieving success in the scheme and understood the basic underlying objectives of enabling access to health care in the target population (Ron, 1999)<br>– In Zaire, the establishment of an incentive system for health workers was critical to enhance workers' commitment to scheme implementations (Criel & Waelkens, 2003).
**Facility-related factor**	
Facility environment	**Total N= 6 studies (2 qualitative studies, 1 cross-sectional study, 3 mixed studies)**<br><br>Barriers (n=4)<br>– In Central Kenya, household survey respondents indicated that corruption and conflict of interest hindered people from joining the scheme and contributed to drop out rates (Mulupi et al., 2013)<br>– In Nigeria, inconvenient facility environment affected household decisions to enroll (Onwujekwe et al., 2009)<br>– In Rwanda, respondents complained that ''health facilities are dirty, lack ambulances, clean bedding and electricity" (P. Schneider, 2005)<br>– In Uganda, members and non-members of CBHI schemes raised quality concerns about the hospital being dirty and long queue (R. Basaza et al., 2008)<br><br>Facilitators (n=2)<br>– In India, cleanliness received the highest score for beneficiary satisfaction (M. Rao et al., 2012)<br>– In Nigeria, availability of good quality treatment was the next most common reason for registering (Uzochukwu et al., 2009)
Supplies and materials	**N=11 studies (4 qualitative studies; 2 cross-sectional studies; 5 mixed studies)**<br><br>Barriers (n=11)<br>– In Central Kenya, poor service provision, exemplified by lack of laboratory equipment and x-ray machines, inadequate ward facilities and poor diet, hindered people from joining health insurance schemes and/or contributed to drop out rates. (Mulupi et al., 2013)<br>– In Tanzania, problems with the quality of services identified included shortage of drugs and essential medical supplies and inappropriate diagnosis due to lack of diagnostic equipment; consequently continuing to low enrollment (Kamuzora & Gilson, 2007)<br>– In Guinea-Conakry, household members reported that ineffective drugs dispensed at the center contributed to failure of insurance scheme to attract more subscribers (Criel & Waelkens, 2003)

	– In Lao PDR, inadequate equipment affected household enrolment in scheme (Alkenbrack et al., 2013)<br>– In Nigeria, lack of equipment affected facility utilization (Uzochukwu et al., 2009)<br>– In Rwanda rural district, lack of drugs contributed to low trust in scheme's ability to improve access to care (P. Schneider, 2005)<br>– In Afghanistan, lack of drug was associated with household perceived low quality of services provided by scheme (K. D. Rao et al., 2009)<br>– In Nigeria, lack of drugs affected success in implementing the scheme (Onwujekwe et al., 2009)<br>– In Uganda, policy makers and district health service managers think that basic medicines and other medical supplies are lacking in public facilities (R. K. Basaza et al., 2010).<br>– In Uganda, members and non-members of CBHI schemes stated that the lack of modern equipment and absence of some prescribed medicines caused low enrolment in the scheme (R. Basaza et al., 2008)<br>– In Burkino Faso, health workers felt that increased pharmacy stock-outs due to the provider payment methods introduced by the scheme impeded their ability to fulfill their professional roles and responsibilities (Robyn et al., 2014).
Interpersonal skills	**Total N= 7 studies (3 qualitative studies; 2 cross-sectional studies; 2 mixed studies)**<br><br>Barriers (n=7)<br>– In Tanzania, problems with the quality of services identified included: staff-related problems; unresponsiveness to patients' problems (wasting time in talking); maltreatment and bad language to patients; and corruption (asking for bribes from patients). This was an important reason for low enrolment in the scheme (Kamuzora & Gilson, 2007).<br>– In Nigeria, 6.3% of respondents from Igboukwu and 14.8% from Neni stated poor staff attitude as a reason for not registering with scheme (Onwujekwe et al., 2009)<br>– In rural Burkina Faso, 19% of respondents stated that a main reason to motivate their decision to discontinue scheme membership was that they didn't like medical staff's behavior (Dong et al., 2009)<br>– In Central Kenya, poor service provision, exemplified by discrimination of patients according to scheme membership or perceived socioeconomic status, hindered people from joining health insurance schemes and/or contributed to drop out rates. Other complaints included poor hospitality, including rude hospital staff (Mulupi et al., 2013)<br>– In Lao PDR, participants complained that CBHI members usually receive low quality drugs, while non-members are prescribed a variety of more expensive drugs.- (Alkenbrack et al., 2013)<br>– In Uganda, household members stated that if attitudes of health workers towards scheme members improved, more members would join and sustainability of the scheme would be achieved (Kyomugisha et al., 2009).<br>– In Rwanda, respondents complained that the providers are unfriendly, unskilled and incompetent and threatened to inform other citizens about their negative experience, which in turn contributes to decreased enrolment (P. Schneider, 2005)
Patient waiting time	**N=3 studies (1 cross-sectional study ; 2 mixed studies)**<br><br>Barriers (n=3)<br>– In Central Kenya, perceived poor service provision, exemplified by long waiting times, hindered people from joining health insurance schemes and/or contributed to drop out rates (Mulupi et al., 2013)

	– In Southeast Nigeria, household members stated delay in services and long waiting hours as a reason hindering enrolment in a CBHI scheme (Onwujekwe et al., 2009)<br>– In Lao PDR, long waiting hour contributed to drop out according to the following scenario: "When CBHI members go to the hospital and show their membership card, the hospital workers ignore them and make them wait for a very long time (Alkenbrack et al., 2013)
**Accessibility of facility**	
Distance to facility	**Total N= 17 studies (9 cross-sectional studies; 2 qualitative studies; 4 mixed studies; 2 case studies)**<br><br><u>Barriers (n=11)</u><br>– In India, utilization rate declined with increasing distance to major cities (M. Rao et al., 2012)<br>– In India, household members reported that selected hospitals in some sub districts were too far away for members to access easily (Ranson et al., 2006)<br>– In Ghana, long distance to facility affected enrolment in the CBHI scheme (Nsiah-Boateng & Aikins, 2013)<br>– In Ghana, the cost of hiring a means of transport in times of emergency was very high and was a major drain on resources in the fund (C. Atim, 2001c)<br>– In Ghana, difficulty in access to remote areas due to lack of transport (C. Atim, 2001b)<br>– In Kenya, almost 30% of respondents reported long distance to health facility as the reason for not seeking health care (Kamau & Njiru, 2014)<br>– In China, poor transportation and remote distance limited enrollees' health services utilization to some degree (Hao, 2010)<br>– In Rwanda, membership begins to taper off as the distance to the health facility increases (P. Schneider, & Diop, F., 2004)<br>– In Uganda, distance to the facility was highlighted by household members one of the main hindrances to benefiting from the scheme (Y. Derriennic, Wolf, K. & Kiwanuka-Mukiibi, P., 2005)<br>– In Southeast Nigeria, 14.1% of participants did not register because they felt the provider facility was too far (Onwujekwe et al., 2009)<br>– In Lao PDR, residents of non-CBHI districts are located three times further from a health facility than CBHI districts driving household enrolment (Alkenbrack et al., 2013).<br><br><u>Facilitator (n=7)</u><br>– In Rwanda, households who live within 30 minutes of their health facility have a 296% higher probability of joining than those who live farther away (P. Schneider, & Diop, F., 2004)<br>– In Senegal, members were more than twice as likely to be situated closer to a health service provider (OR=2.25) and were three times more likely to report that health care access is an advantage of membership (OR=3.05) (Mladovsky, 2014)<br>– In Nigeria, nearness of health facility and adequacy of facilities were indicated as reasons for increased facility utilization (Onwujekwe et al., 2009)<br>– In Burkina Faso, shorter distance to contracted health facility positively influenced dropout from CBHI scheme (OR=0.36; p=0.05) (Dong et al., 2009)<br>– In Thailand, healthcare-seeking pattern among card users and non-card users strongly supported the importance of accessibility to health care among the card user group in the scheme (Supakankunti, 2000)

	– In Senegal, people living in Fandène have a higher effective demand for health insurance than people in other communities as it is also the closest mutual to St. Jean de Dieu Hospital (Jütting, 2004)<br>– In India, urban women were three times more likely to enroll than rural women due to better outreach and accessibility factors (Gumber, 2004)
Choice of facility	**Total N= 3 studies (3 qualitative studies)**<br><br><u>Barriers (n=2)</u><br>– In Burkina Faso, entire villages were assigned to one specific local first-line facility which restricted household access to services (De Allegri et al., 2006)<br>– In Tanzania, lack of possibility to use health facilities of members' choice influencing low enrolment in the CBHI scheme (Kamuzora & Gilson, 2007)<br><br><u>Facilitator (n=1)</u><br>– In Uganda, expanding the pool of affiliated providers so that members can obtain outpatient care at clinics closer to their homes was highlighted as a good model for sustainability of the scheme (Y. Derriennic, Wolf, K. & Kiwanuka-Mukiibi, P., 2005).
Referral systems	**Total N= 5 studies (1 cross-sectional study; 2 qualitative studies; 2 case studies)**<br><br><u>Barriers (n=3)</u>:<br>– In Tanzania, lack of comprehensive service coupled with lack of referral system was reported by district and community respondent group contributed to low perceived quality of care and consequently, low enrollment (Kamuzora & Gilson, 2007).<br>– In Thailand, the absence of a referral system between the different levels of care gave rise to the problem of bypassing the health centers, which in turn influenced scheme performance (Supakankunti, 2004).<br>– In Ghana, the lack of a nearby health facility to give first line treatment to members before transferring to hospital only when necessary was a major concern and challenge (C. Atim, 2001c)<br><br><u>Facilitators (n=2)</u>:<br>– In Zaire, the presence of a strong referral system helped offset inappropriate hospital utilization (Criel & Kegels, 1997).<br>– In Philippine, the presence of referral process for hospital-based services was one of the factors contributing to the success of the scheme.(Ron, 1999)
**Marketing and Promotion Strategies**	
Adequacy of campaigns	**Total N= 13 studies (2 cross-sectional studies; 5 qualitative studies; 2 case studies; 3 mixed method studies)**<br><br><u>Barriers (n=8)</u><br>– In Senegal, diversified access to information was a determinant of enrolment, with interviewees complaining that information about CBHI schemes was scarce (Mladovsky, 2014)<br>– In India, household members reported lack of information on availability and location of services provided by scheme which affected accessibility (Ranson et al., 2006)<br>– In India, beneficiary satisfaction survey elicited the lowest scores for information provided about the scheme (M. Rao et al., 2012)<br>– In Uganda, sensitization has been adequately done and the content of sensitization needed to be tailored to the core principles of CBHI (R. Basaza et al., 2008)

	– In Cameroon, poor awareness about the scheme from the informal sectors workers' point of view was probably due to inadequate public sensitization through the mass media (Noubiap et al., 2013)<br>– In Uganda, the absence of any specific national conference, guidelines, or deliberate attempts by the MOH to promote community-health insurance contributed to the low level of knowledge of CHI by MOH staff, district managers, and health professionals, (R. K. Basaza et al., 2010).<br>– In Kenya, participants attributed the lack of awareness of health insurance to limited efforts to promote CBHIs (Mulupi et al., 2013)<br>– In Burkina Faso, the campaign was perceived as focusing heavily on rural areas to the disadvantage of urban residents, which created concerns since rural residents relied on their relatives in urban areas to act on information (Cofie et al., 2013a)<br><br><u>Facilitators (n=3)</u><br>– In Guinea-Conakry, people reported adhering to the scheme because of convincing information campaigns (Criel & Waelkens, 2003)<br>– In Philippine, registration increased following competitive promotion campaigns at community level and spreading of word of positive experience (Ron, 1999)<br>– In Uganda, a sensitized member base could make educated decisions to protect the financial viability of the scheme (Y. Derriennic, Wolf, K. & Kiwanuka-Mukiibi, P., 2005)
Marketing technique	**Total N= 4 studies (1 qualitative, 1 cross-sectional study, 1 mixed studies; 1 case study)**<br><br><u>Barriers (n=2)</u><br>– In Uganda, absence of marketing research to show which marketing strategies can be successfully employed to promote membership served as an obstacle to sustainability (Y. Derriennic, Wolf, K. & Kiwanuka-Mukiibi, P., 2005)<br>– In Ghana, inappropriate social marketing and community mobilization technique affected enrolment in CBHI scheme (C. Atim, 2001b)<br><br><u>Facilitator (n=2)</u><br>– In Burkina Faso, intensity of exposure to campaign channels was positive and significant determinants of enrolment (Cofie et al., 2013a)<br>– In Rwanda, households who owned a radio were 47% more likely to enroll in a CBHI scheme than those without a radio, which is likely related to the regular awareness campaigns transmitted by radio (P. Schneider, & Diop, F., 2004)

References

- Alkenbrack, S., Jacobs, B., & Lindelow, M. (2013). Achieving universal health coverage through voluntary insurance: what can we learn from the experience of Lao PDR? *BMC Health Serv Res, 13*, 521. doi:10.1186/1472-6963-13-521
- Atim, C. (2001). A survery of health financing schemes in Ghana. *PHRplus*.
- Atim, C. (2001a). A survery of health financing schemes in Ghana-Nkoranza Community Health Financing Scheme *PHRplus*.
- Atim, C. (2001b). A survery of health financing schemes in Ghana- Dodowa Community Health Insurance Scheme
- *PHRplus*.
- Atim, C. (2001c). A survery of health financing schemes in Ghana- Tiyumtaaba Community Health Financing Scheme *PHRplus*.
- Basaza, R., Criel, B., & Van der Stuyft, P. (2007). Low enrollment in Ugandan Community Health Insurance schemes: underlying causes and policy implications. *BMC Health Serv Res, 7*, 105. doi:10.1186/1472-6963-7-105
- Basaza, R., Criel, B., & Van der Stuyft, P. (2008). Community health insurance in Uganda: why does enrolment remain low? A view from beneath. *Health Policy, 87*(2), 172-184. doi:10.1016/j.healthpol.2007.12.008
- Basaza, R. K., Criel, B., & Van der Stuyft, P. (2010). Community health insurance amidst abolition of user fees in Uganda: the view from policy makers and health service managers. *BMC Health Serv Res, 10*, 33. doi:10.1186/1472-6963-10-33
- Bhat, R., & Jain, N. . (2006). Factoring affecting the demand for insurance in a micro health insurance scheme. IIMA, India.
- Cofie, P., De Allegri, M., Kouyate, B., & Sauerborn, R. (2013a). Effects of information, education, and communication campaign on a community-based health insurance scheme in Burkina Faso. *Glob Health Action, 6*, 20791. doi:10.3402/gha.v6i0.20791
- Cofie, P., De Allegri, M., Kouyate, B., & Sauerborn, R. (2013b). Effects of information, education, and communication campaign on a community-based health insurance scheme in Burkina Faso. *Glob Health Action, 6*(1), 20791. doi:10.3402/gha.v6i0.20791
- Criel, B., & Kegels, G. (1997). A health insurance scheme for hospital care in Bwamanda District, Zaire: lessons and questions after 10 years of functioning. *Trop Med Int Health, 2*(7), 654-672.
- Criel, B., & Waelkens, M. P. (2003). Declining subscriptions to the Maliando Mutual Health Organisation in Guinea-Conakry (West Africa): what is going wrong? *Soc Sci Med, 57*(7), 1205-1219.

- De Allegri, M., Sanon, M., Bridges, J., & Sauerborn, R. (2006). Understanding consumers' preferences and decision to enrol in community-based health insurance in rural West Africa. *Health Policy, 76*(1), 58-71. doi:10.1016/j.healthpol.2005.04.010
- Derriennic, Y., Wolf, K., & Kiwanuka-Mukiibi, P. (2005). An assessment of community-based health financing activities in Uganda.
- Derriennic, Y., Wolf, K. & Kiwanuka-Mukiibi, P. (2005). An assessment of community-based health financing activities in Uganda. *USAID*.
- Dong, H., De Allegri, M., Gnawali, D., Souares, A., & Sauerborn, R. (2009). Drop-out analysis of community-based health insurance membership at Nouna, Burkina Faso. *Health Policy, 92*(2-3), 174-179. doi:10.1016/j.healthpol.2009.03.013
- Gnawali, D. P., Pokhrel, S., Sie, A., Sanon, M., De Allegri, M., Souares, A., . . . Sauerborn, R. (2009). The effect of community-based health insurance on the utilization of modern health care services: evidence from Burkina Faso. *Health Policy, 90*(2-3), 214-222. doi:10.1016/j.healthpol.2008.09.015
- Gumber, A. (2004). The potential role of community financing in India. Health Financing for Poor People, 293.
- Haddad, S., Narayana, D., & Mohindra, K. (2011). Reducing inequalities in health and access to health care in a rural Indian community: an India-Canada collaborative action research project. *BMC Int Health Hum Rights, 11 Suppl 2*, S3. doi:10.1186/1472-698X-11-S2-S3
- Hao, Y., Wu, Q., Zhang, Z., Gao, L., Ning, N., Jiao, M., & Zakus, D. (2010). The impact of different benefit packages of Medical Financial Assistance Scheme on health service utilization of poor population in Rural China. . *BMC health services research, 10*(1), 170.
- Ito, S., & Kono, H. . (2010). WHY IS THE TAKE-UP OF MICROINSURANCE SO LOW? EVIDENCE FROM A HEALTH INSURANCE SCHEME IN INDIA. . *The Developing Economies, 48*(1), 74-101.
- Jütting, J. (2004). Financial Protection and Access to Health Care in Rural Areas of Senegal-Health financing for poor people—Resource mobilization and risk sharing, Washington DC: World Bank, 251-274.

- .
- Kamau, N., & Njiru, H. (2014). Community based health insurance schemes: lessons from rural Kenya. *J Health Care Poor Underserved, 25*(1), 192-203. doi:10.1353/hpu.2014.0023
- Kamuzora, P., & Gilson, L. (2007). Factors influencing implementation of the Community Health Fund in Tanzania. *Health Policy Plan, 22*(2), 95-102. doi:10.1093/heapol/czm001
- Kiwara, A. D. (2007). Group premiums in micro health insurance experiences from Tanzania. *East Afr J Public Health, 4*(1), 28-32.
- Kyomugisha, E. L., Buregyeya, E., Ekirapa, E., Mugisha, J. F., & Bazeyo, W. (2009). Strategies for sustainability and equity of prepayment health schemes in Uganda. *Afr Health Sci, 9 Suppl 2*, S59-65.
- Lammers J, W. S., Ecorys R. . (2010). Adverse selection in voluntary micro health insurance in Nigeria. . *AIDS Research Series.*(6).
- Mladovsky, P. (2014). Why do people drop out of community-based health insurance? Findings from an exploratory household survey in Senegal. *Soc Sci Med, 107*, 78-88. doi:10.1016/j.socscimed.2014.02.008
- Mladovsky, P., Soors, W., Ndiaye, P., Ndiaye, A., & Criel, B. (2014). Can social capital help explain enrolment (or lack thereof) in community-based health insurance? Results of an exploratory mixed methods study from Senegal. *Soc Sci Med, 101*, 18-27. doi:10.1016/j.socscimed.2013.11.016

- Mulupi, S., Kirigia, D., & Chuma, J. (2013). Community perceptions of health insurance and their preferred design features: implications for the design of universal health coverage reforms in Kenya. *BMC Health Serv Res, 13*, 474. doi:10.1186/1472-6963-13-474
- Noubiap, J. J., Joko, W. Y., Obama, J. M., & Bigna, J. J. (2013). Community-based health insurance knowledge, concern, preferences, and financial planning for health care among informal sector workers in a health district of Douala, Cameroon. *Pan Afr Med J, 16*, 17. doi:10.11604/pamj.2013.16.17.2279
- Nsiah-Boateng, E., & Aikins, M. (2013). Performance Assessment of Ga District Mutual Health Insurance Scheme, Greater Accra Region, Ghana. *Value in Health Regional Issues, 2*(2), 300-305. doi:http://dx.doi.org/10.1016/j.vhri.2013.06.005
- Onwujekwe, O., Onoka, C., Uzochukwu, B., Okoli, C., Obikeze, E., & Eze, S. (2009). Is community-based health insurance an equitable strategy for paying for healthcare? Experiences from southeast Nigeria. *Health Policy, 92*(1), 96-102. doi:10.1016/j.healthpol.2009.02.007
- Ouimet, M.-J., Fournier, P., Diop, I., & Haddad, S. (2007). Solidarity or financial sustainability: an analysis of the values of community-based health insurance subscribers and promoters in Senegal. *Canadian Journal of Public Health/Revue Canadienne de Sante'e Publique*, 341-346.
- Ouimet, M. J., Fournier, P., Diop, I., & Haddad, S. (2007). Solidarity or financial sustainability: an analysis of the values of community-based health insurance subscribers and promoters in Senegal. *Can J Public Health, 98*(4), 341-346.
- Ozawa, S., & Walker, D. G. (2009). Trust in the context of community-based health insurance schemes in Cambodia: villagers' trust in health insurers. *Adv Health Econ Health Serv Res, 21*, 107-132.
- Panda, P., Chakraborty, A., Dror, D. M., & Bedi, A. S. (2014). Enrolment in community-based health insurance schemes in rural Bihar and Uttar Pradesh, India. *Health Policy Plan, 29*(8), 960-974. doi:10.1093/heapol/czt077
- Parmar, D., Souares, A., de Allegri, M., Savadogo, G., & Sauerborn, R. (2012). Adverse selection in a community-based health insurance scheme in rural Africa: implications for introducing targeted subsidies. *BMC Health Serv Res, 12*, 181. doi:10.1186/1472-6963-12-181
- Polonsky, J., Balabanova, D., McPake, B., Poletti, T., Vyas, S., Ghazaryan, O., & Yanni, M. K. (2009). Equity in community health insurance schemes: evidence and lessons from Armenia. *Health Policy Plan, 24*(3), 209-216. doi:10.1093/heapol/czp001
- Ranson, M. K. (2004). The impact of sewa's medical insurance fund on hospital utilization and expenditure: a household survey. The World Bank.
- Ranson, M. K., Sinha, T., Gandhi, F., Jayswal, R., & Mills, A. J. (2006). Helping members of a community-based health insurance scheme access quality inpatient care through development of a preferred provider system in rural Gujarat. *Natl Med J India, 19*(5), 274-282.
- Rao, K. D., Waters, H., Steinhardt, L., Alam, S., Hansen, P., & Naeem, A. J. (2009). An experiment with community health funds in Afghanistan. *Health Policy Plan, 24*(4), 301-311. doi:10.1093/heapol/czp018
- Rao, M., Kadam, S., Sathyanarayana, T., Shidhaye, R., Shukla, R., Ramachandra, S. S., . . . Shatrugna, V. (2012). A rapid evaluation of the Rajiv Aarogyasri community health insurance scheme in Andhra Pradesh, India. *BMC Proceedings, 6*(1), O4. doi:10.1186/1753-6561-6-s1-o4
- Robyn, P. J., Barnighausen, T., Souares, A., Traore, A., Bicaba, B., Sie, A., & Sauerborn, R. (2014). Provider payment methods and health worker motivation in community-based health insurance: a mixed-methods study. *Soc Sci Med, 108*, 223-236. doi:10.1016/j.socscimed.2014.01.034
- Ron, A. (1999). NGOs in community health insurance schemes: examples from Guatemala and the Philippines. *Soc Sci Med, 48*(7), 939-950.

- Schneider, P. (2005). Trust in micro-health insurance: an exploratory study in Rwanda. *Soc Sci Med, 61*(7), 1430-1438. doi:10.1016/j.socscimed.2004.11.074
- Schneider, P., & Diop, F. (2004). Community-based health insurance in Rwanda. Health financing for poor people—Resource mobilization and risk sharing, Washington DC: World Bank, 251-274.
- Shaw, R. P. (2002). Tanzania's community health fund: prepayment as an alternative to user fees.
- Supakankunti, S. (2000). Future prospects of voluntary health insurance in Thailand. *Health Policy Plan, 15*(1), 85-94.
- Supakankunti, S. (2004). Impact of the Thailand Health Card. Health financing for poor people—Resource mobilization and risk sharing, Washington DC: World Bank, 251-274.
- .
- Uzochukwu, B., Onwujekwe, O., Eze, S., Ezuma, N., Obikeze, E., & Onoka, C. (2009). Community based health insurance scheme in Anambra State, Nigeria: an analysis of policy development, implementation and equity effects. *London: Consortium for Research on Equitable Health Systems, London School of Hygiene and Tropical Medicine*.
- Wang, H., Zhang, L., Yip, W., & Hsiao, W. (2006). Adverse selection in a voluntary Rural Mutual Health Care health insurance scheme in China. *Soc Sci Med, 63*(5), 1236-1245. doi:10.1016/j.socscimed.2006.03.008
- Zhang, L., & Wang, H. (2008). Dynamic process of adverse selection: evidence from a subsidized community-based health insurance in rural China. *Soc Sci Med, 67*(7), 1173-1182. doi:10.1016/j.socscimed.2008.06.024
